# Regio- and Stereoselective Electrochemical Alkylation of Morita–Baylis–Hillman Adducts

**DOI:** 10.1021/acs.orglett.2c01529

**Published:** 2022-06-14

**Authors:** Giulio Bertuzzi, Giada Ombrosi, Marco Bandini

**Affiliations:** † Dipartimento di Chimica “Giamician Ciamician”, Alma Mater Studiotum − Università di Bologna, Via Selmi 2, 40126 Bologna, Italy; ‡ Center for Chemical Catalysis -C3-, Alma Mater Studiotum − Università di Bologna, Via Selmi 2, 40126 Bologna, Italy

## Abstract

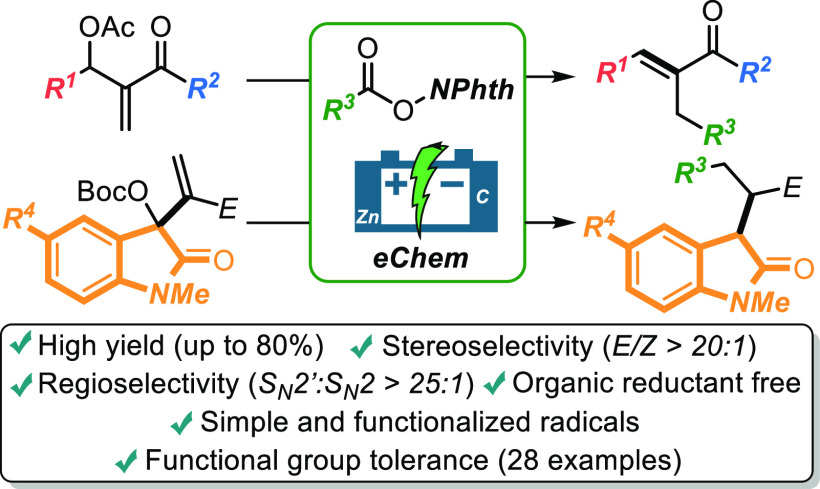

Electrosynthesis is effectively employed in a general regio- and stereoselective alkylation of Morita–Baylis–Hillman compounds. The exposition of *N*-acyloxyphthalimides (redox-active esters) to galvanostatic electroreductive conditions, following the sacrificial-anode strategy, is proved an efficient and practical method to access densely functionalized cinnamate and oxindole derivatives. High yields (up to 80%) and wide functional group tolerance characterized the methodology. A tentative mechanistic sketch is proposed based on dedicated control experiments.

Morita–Baylis–Hillman (MBH) adducts (**1**) are arguably referred to as “privileged scaffolds” within the synthetic community, due to their ease of preparation,^1^ ready diversification and faceted reactivity. In recent years, S_N_2 or S_N_2′ substitution reactions on MBH compounds has faced high interest with a variety of ionic-based nucleophiles,^2^ as well as their transformation in reactive dipolar species.^3^ These led to the development of a large number of uncatalyzed or Lewis-based catalyzed protocols, targeting, among others, functionalized methacrylates, cinnamates, azine heterocycles, and indole derivatives.^4^ On the contrary, the rapidly expanding radical chemistry scenario has scarcely permeated the functionalization of MBH adducts. Here, some elegant examples have recently been disclosed under metal- or photocatalytic regimes with a focus on specific classes of stabilized alkyl radicals (Scheme 1a).^5,6^ Protocols displaying a wide scope of simple and functionalized radicals are much more narrow in number, with notable shortcomings related to the employment of stoichiometric organic reductants^7^ or poor stereochemical outcomes.^8^ Proceeding under mild conditions, displaying exquisite functional group tolerance and complementary selectivity, electrochemical synthesis “**eChem**” has rapidly paralleled other enabling techniques, opening unforeseen opportunities in organic synthesis.

**Scheme 1:**
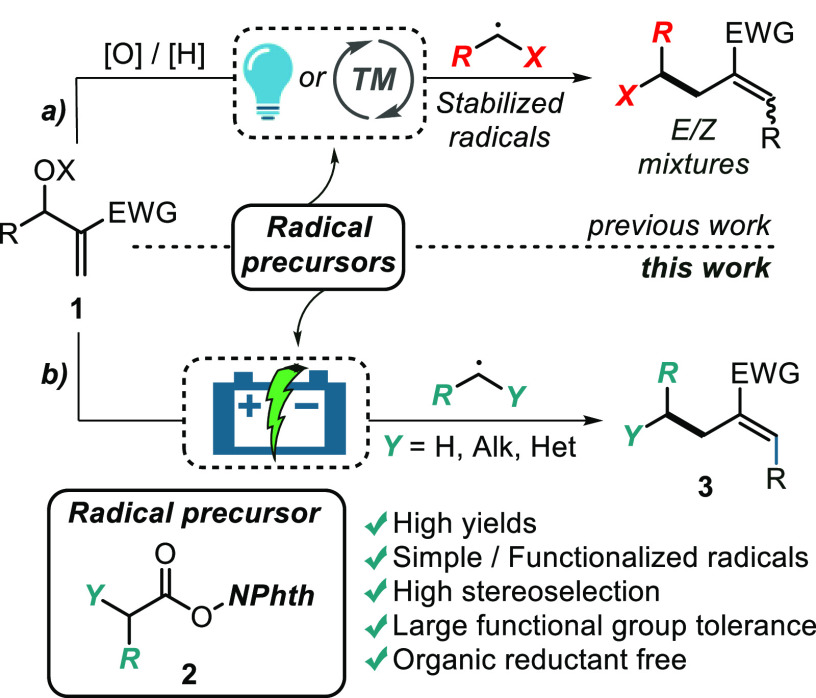
Previous Methodologies for the Radical Alkylation of MBH Acetates and Present **eChem** Protocol NPhth: phthalimide.

Our current research interest toward the implementation of site-selective radical-based transformations^9^ prompted us to envision **eChem** as a valuable direct toolbox for the functionalization of MBH adducts **1** with “green electrons”.^10^

To realize such a strategy, we deemed a general, cheap, easy to prepare, and broadly applicable class of radical precursors to be necessarily employed. *N-*Acyloxyphthalimides (redox-active esters, RAE, **2**), prepared in one step from inexpensive *N*-hydroxyphthalimide and ubiquitous carboxylic acids, represent a class of benchmark radical precursors.^11^ RAEs have been extensively employed in metal- or photoassisted^12^ and (to a lower extent) electrochemical generation of radicals under *reductive* conditions.^13^ Among others, C–H functionalizations and cross-coupling processes were primary targeted,^14^ whereas the use of RAEs on **eChem** derivatizations of olefins have faced less attention.^15^ In particular, the condensation of RAEs **2** and MBH acetates **1** is unprecedented (Scheme 1b), posing some important questions toward the overall chemo- and regioselectivity of the process. In this report, we disclose a highly stereoselective (the *E*-isomers were exclusively isolated), electroreductive strategy for the regioselective S_N_2′ radical alkylation of MBH adducts **1** with RAEs **2**. The protocol targets the formation of α-substituted α,β-unsaturated esters or ketones **3**, under mild and organic reductant-free conditions. It is worth mentioning that alternative synthetic methodologies for the installation of alkyl (i.e., methyl) groups at the β-postion of the MBH acceptors requires the utilization of harmful organometallic reagents such as trialkyl-Al and trialkyl-In compounds.^16^

Our investigation started by subjecting MBH acetate **1a** and RAE **2a** (1 equiv) to a constant current electrolysis of 10 mA.^17^ A graphite (C) cathode and a Zn sacrificial anode were used as electrodes with tetraethylammonium tetrafluoroborate (TEABF_4_, 1 equiv) as the supporting electrolyte in DMF (Table 1, entry 1). Encouragingly, the desired product **3aa** was isolated in 25% yield as a single *E* isomer, along with 5% of the reduction–deacetoxylation product **1a′**. Exclusive S_N_2′ radical trapping was recorded, and no over-reduced product **3aa′** was detected. The high *E* selectivity of cinnamates **3** means the present **eChem** approach is a desirable and complementary alternative to the poorly stereoselective photocatalytic strategy, aiming at similar tasks (vide infra).^8^ Moreover, the use of a nontoxic and inexpensive sacrificial anode (Zn) circumvents the use of organic reductants whose cost and separation from the reaction mixture might be burdensome.

**Table 1:**
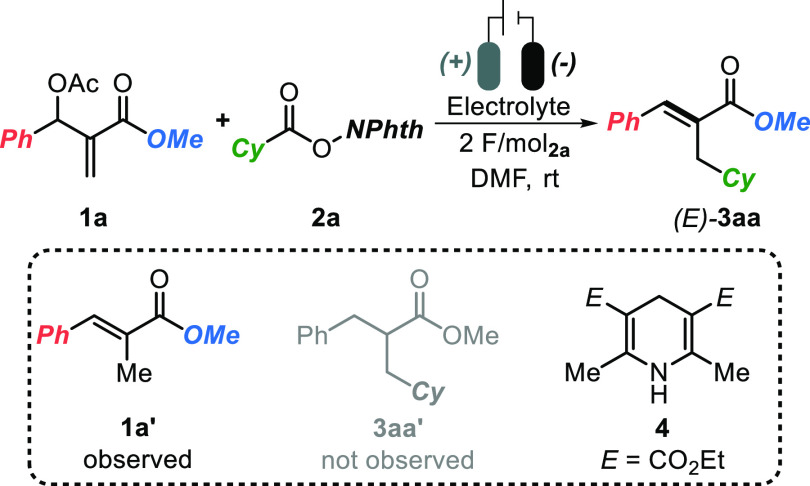
Optimization of the Reaction Conditions.^a^

entry	electrolyte (equiv)	anode (+)||cathode (−)	*I* (mA)	yield^b^ (%)
1^c^	TEABF_4_ (1)	Zn(+)||C(−)	10	25
2^c^	TEABF_4_ (2)	Zn(+)||C(−)	10	38
3	TEABF_4_ (2)	Zn(+)||C(−)	10	59
4	TEABF_4_ (2)	Zn(+)||C(−)	4	67
**5**	**TEABF_4_ (2)**	**Zn(+)||C(−)**	**2**	**79**
6	TBAPF_6_ (2)	Zn(+)||C(−)	4	65
7	LiBF_4_ (2)	Zn(+)||C(−)	4	66
8	TEABF_4_ (2)	Zn(+)||RVC(−)	4	65
9	TEABF_4_ (2)	Zn(+)||Ni^d^(−)	4	54
10	TEABF_4_ (2)	Mg(+)||C(−)	4	54
11	TEABF_4_ (2)	Ni(+)||C(−)	4	50
12^e^	TEABF_4_ (2)	C(+)||C(−)	4	34

a Reaction conditions, unless otherwise noted: **1a** (0.15 mmol), **2a** (0.30 mmol), electrolyte (0.15 or 0.30 mmol), dry DMF (3 mL), CCE (10, 4, or 2 mA; 2 F/mol_**2a**_), rt. *E*/*Z* ratios were determined via ^1^H NMR spectroscopy on the reaction crude mixtures and were always found to be >20:1.

b Isolated yields after flash chromatography.

c **2a** (0.15 mmol).

d Ni foam.

e **4** (0.30 mmol) added.

In order to optimize the reaction conditions, the equivalents of both the electrolyte (2 equiv, entry 2, 38% yield) and of **2a** (2 equiv, entry 3, 59% yield) were beneficially increased. Next, we reasoned that a slower generation of the radical species, as the result of a lower current, could be beneficial in avoiding radical–radical homocoupling and other undesired side reactions. Interestingly, by lowering the current value from 10 to 4 mA (entry 4, 67% yield) and further to 2 mA (entry 5, 79% yield) a significant increase in reaction efficiency was recorded (with 2 mA current, the formation of **1a′** was completely suppressed). On the other hand, salts such as tetrabutylammonium hexafluorophosphate (TBAPF_6_, entry 6) and LiBF_4_ (entry 7) behaved similarly to TEABF_4_. Cathodes different from graphite, such as RVC (reticulated vitreous carbon, entry 8) and Ni foam (entry 9), and sacrificial anodes such as Mg and Ni (entries 10 and 11), delivered **3aa** in comparable or lower yields with respect to the optimal set (entry 5). To demonstrate the superiority of the strategy based on the sacrificial anode, an attempt using Hantzsch ester **4** as the terminal reductant and two graphite (C) electrodes was performed (entry 12).^15a^ Here, product **3a** was isolated in low yield (34%) along with 22% of reduced byproduct **1a’**.

Having established the optimal reaction conditions (Table 1, entry 5), the generality of the electrochemical alkylation was tested (Scheme 2) on different MBH acetates (or carbonates) 1 (or 5). Cinnamates **3**, having both electron-withdrawing (**3ba**–**3da**, **3ga**) and electron-donating (**3ea**, **3fa**) substituents at the *para* or *ortho* position of the benzene ring, were productively formed (61–77% yield). Naphthalene (**1h**) and heteroarenes (thiophene **1i** and quinoline **1j**) on the MBH acceptor were also well tolerated, although in the case of **1j** a complete reduction of the C–C double bond occurred, resulting in saturated compound **3ja′** as the sole product (64%). Pleasingly, MBH acetate **1k**, derived from an aliphatic aldehyde (i.e., hydrocinnamaldehyde) was proved to be productive (**3ka**, 62% yield) and product **3la**, having a conjugated diene moiety, could also be formed in synthetically useful 57% yield. It is worth mentioning that radical-sensitive moieties such as benzylic (**1m**) and propargylic esters (**1n**) were adequately tolerated in the present eChem alkylation process (yield: 76% and 74%, respectively). Finally, the protocol was not limited to ester-like MBH adducts; as a matter of fact, when methyl ketone **1o** was subjected to the optimal conditions the desired α-alkylated enone **3oa** was isolated in synthetically useful amounts (76% yield).

**Scheme 2:**
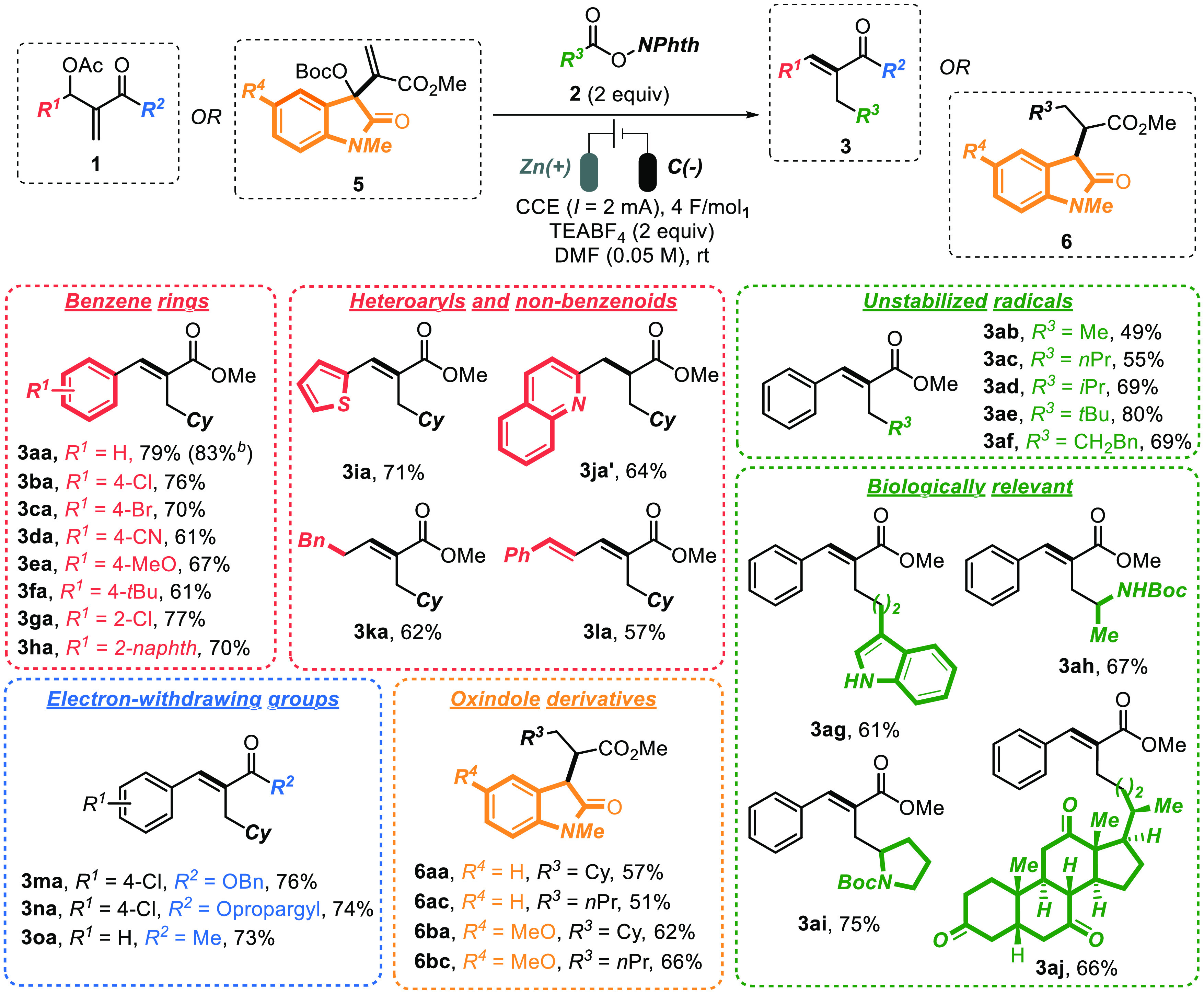
Scope of the Present **eChem** Methodology Reaction conditions: **1** (0.15 mmol), **2** (0.3 mmol), TEABF_4_ (0.30 mmol), dry DMF (3 mL), CCE (2 mA; 4 F/mol_1_), Zn(+) C(−), rt. Isolated yields after flash chromatography; *E*/*Z* ratios were determined via ^1^H NMR spectroscopy on the reaction crude mixtures. Reaction performed on 1.0 mmol of **1a** at 3.0 mA, see the Supporting Information for details.

Additionally, the electrochemical alkylation protocol was extended to MBH carbonates derived from *N*-methylisatins (**5a**,**b**) in the presence of RAEs **2a** and **2c**. Delightfully, the corresponding oxindoles **6** were isolated with high diastereoselectivity (>20:1) and useful yields (51–66%) as a result of an alkylation–reduction sequence. To the best of our knowledge, this protocol represents the first example of S_N_2′-type radical alkylations of oxindole-based MBH acceptors. The exclusive isolation of the fully reduced compounds **6** and **3ja′** might be rationalized in term of lower reduction potential of α,β-unsaturated esters featuring conjugated highly electron-deficient moieties. Over-reductive SET processes and hydrogen abstraction of the resulting radical anions could lead to “zinc-enolate” intermediates that will smoothly undergo protonation during the aqueous reaction quenching.

Next, we examined the adaptability of the disclosed strategy to different radical precursors **2b**–**j**. Methyl (**2b**), primary (**2c**), secondary (**2d** and **2f**), and tertiary (**2e**) alkyl radicals were all installed regio- and stereoselectively at MBH acetate **1a**, highlighting the generality of the methodology (**3ab**–**af**, 49–80% yield). As a rule of thumb, the higher the substitution (hence, the stability) of the radical the higher the yield observed. Tolerance toward an unprotected indole group was also ascertained (**3ag**, 61% yield). Pleasingly, amino acid derived RAEs **2h** (Boc-Ala) and **2i** (Boc-Pro) as well as dehydrocholic acid derived **2j** generated competent alkyl radicals for the alkylation of **1a** (**3ah-aj**, 66–75% yield). This demonstrates the possibility to employ naturally occurring acids as precursors of functionalized radicals as well as developing bioconjugation protocols via a late-stage **eChem** approach. The millimole scale reaction on the model substrates **1a** and **2a** was also effectively carried out, delivering the desired **3aa** in 83% isolated yield.

To gain mechanistic insights into the formation of byproduct **1a′**, voltametric experiments on **1a** were then carried out (see the Supporting Information). Unfortunately, no clear evidence for a reductive event was recorded, suggesting that **1a′** could result from successive reactive steps. However, a control experiment run on **1a** in the absence of **2a** (optimal reaction conditions) furnished **1a′** in 18% yield, along with poor recovery of unreacted **1a** (34%, decomposition to unidentified byproducts, Scheme 3a). This result rules out an exclusive RAE-mediated formation of **1′** from **1** and proves MBH acceptors **1** are not inert toward reductive conditions, rendering a judicious choice of the reaction parameters pivotal for their productive employment in electroreductive processes.

**Scheme 3:**
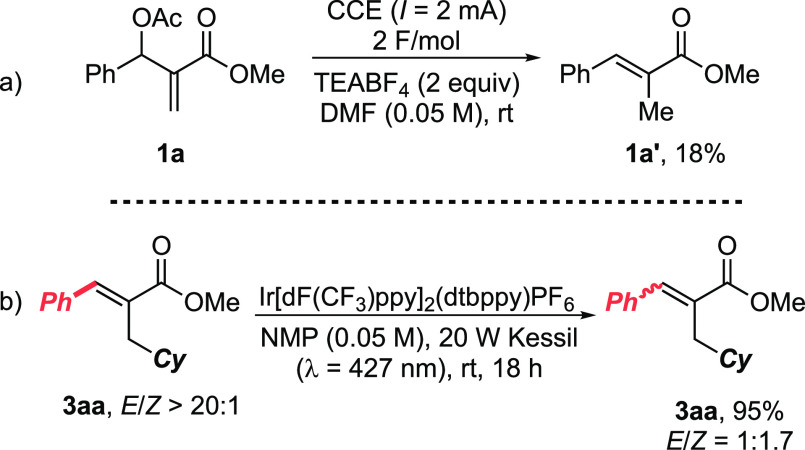
Control Experiments

Finally, our attention was caught by the stereochemical discrepancy between our protocol (*E*/*Z* > 20:1) and previously reported photochemical radical alkylations of MBH derivatives (*E*/*Z* ca. 1:1).^8,18^ To prove a possible photomediated isomerization of the final cinnamyl C–C double bond, we subjected (*E*)-**3aa** (*E*/*Z* > 20:1) to visible-light irradiation in the presence of a common triplet-emitter Ir-based photosensitizer (Ir[dF(CF_3_)ppy]_2_(dtbppy)PF_6_, (1 mol %, Scheme 3b). Interestingly, **3aa** was recovered in 95% yield as a 1:1.7 *E*/*Z* mixture, showing the intrinsic unsuitability of some photocatalytic methodologies aiming at the stereoselective formation of α-substituted cinnamates.^19^

Mechanistically, the **eChem** cycle depicted in Scheme 4 is tentatively proposed. This starts with the cathodic fragmentation of **2a** into CO_2_, phthalimide anion, and the desired cyclohexyl radical. This event is in accordance with cyclovoltammetry experiments run on **2a**, showing a clear irreversible cathodic event at −1.57 V vs ferrocene (−1.15 V vs Ag/AgCl).^15a^ The possible involvement of the MBH adduct into cathodic reduction was judged unlikely due to the absence of reductive signals on the collected voltametric spectra in the same reductive windows of **2a** (see the Supporting Information). Addition of the cyclohexyl radical onto the electrophilic double bond of **1a** gives the key intermediate **A**, which can evolve following two alternative pathways. Radical fragmentation (path a) would directly render the observed product **3aa** and the acetoxy radical, undergoing reduction to the acetate anion. On the other hand, intermediate **A** can first be reduced and then deliver **3aa** through E1cB elimination. As depicted, cathodic reductions are coupled with anodic formation of Zn^2+^ ions, following the sacrificial anode strategy.

**Scheme 4:**
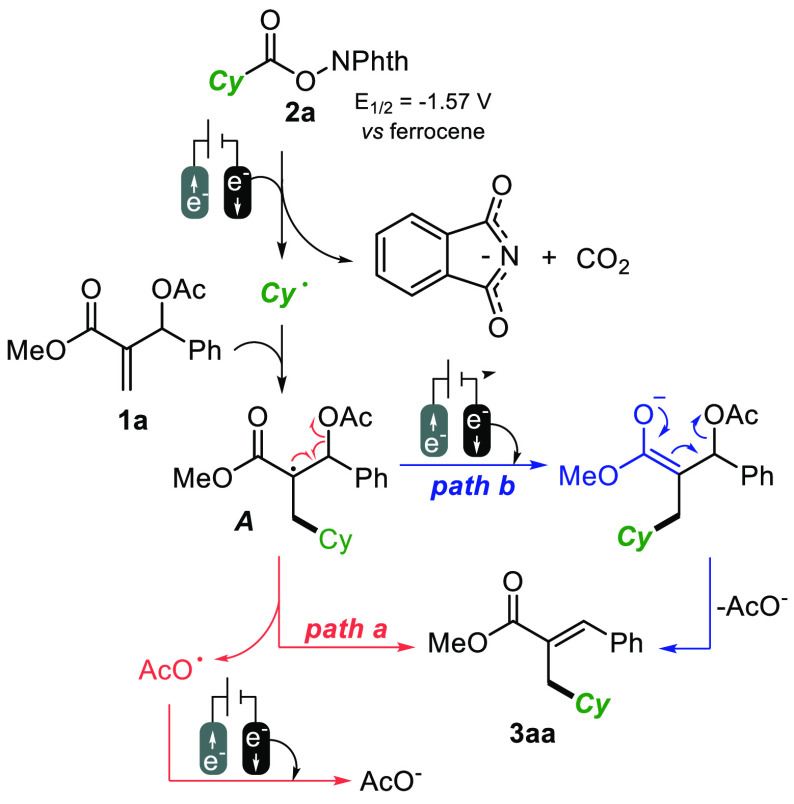
Proposed Mechanistic Profile

In conclusion, we have developed a novel electrochemical alkylation of MBH adducts with redox-active esters as radical precursors. Under galvanostatic conditions and employing a sacrificial anode, a wide range of α-substituted α,β-unsaturated esters or ketones were formed chemo- and stereoselectively (*E/Z* ratio >20:1). Extension of the protocol to isatin-derived MBH carbonates resulted in the formation of substituted oxindoles following an alkylation–reduction sequence. Control experiments and mechanistic proposals completed the present investigation.

## Supporting Information Available

Additional screening of reaction conditions, detailed experimental procedures, characterization data, CV experiments and NMR spectra (PDF)
