# Supplementary material for: Regio- and Stereoselective Electrochemical Alkylation of Morita–Baylis–Hillman Adducts
Source: Org Lett. 2022 Jun 14;24(24):4354–9. doi: 10.1021/acs.orglett.2c01529 (PMC9237826; doi:10.1021/acs.orglett.2c01529)
Supplement: Supplementary file 1 — ol2c01529_si_001.pdf [file ol2c01529_si_001.pdf]

Supporting Information

Regio- and Stereoselective Electrochemical Alkylation of Morita-Baylis-Hillman Adducts.

Giulio Bertuzzi,[a,b]* Giada Ombrosi,[a] and Marco Bandini[a,b]*

[a] Dipartimento di Chimica "Giacomo Ciamician", Alma Mater Studiorum – Università di Bologna, via Selmi 2, 40126 Bologna, Italy

[b] Center for Chemical Catalysis – C^3, via Selmi 2, 40126, Bologna, Italy

Emails: giulio.bertuzzi2@unibo.it; marco.bandini@unibo.it

Table of Contents

General methods	S2
Table S1. Optimization of reaction conditions, additional data	S3
Limitation of the methodologies: unsuccessful substrates	S5
Cyclovoltammetry Experiments	S6
Photoisomerization of ***E*-3aa**	S8
Optimized general procedure for **eChem** alkylation of MBH adducts	S9
^{1}H and ^{13}C NMR spectra	S17
Copies of selected HRMS spectra	S45
References	S51

General Methods.

^{1}H-NMR spectra were recorded on Varian 400 (400 MHz) spectrometer. Chemical shifts are reported in ppm from TMS with the solvent resonance as the internal standard (deuterochloroform: 7.24 ppm). Data are reported as follows: chemical shift, multiplicity (s = singlet, d = doublet, dd= doublet doublet, t = triplet, td = triple doublet, dt = double triplet, q = quartet, sext = sextet, sept = septet, p = pseudo, b = broad, m = multiplet), coupling constants (Hz). ^{13}C-NMR spectra were recorded on a Varian 400 (100 MHz) spectrometer with complete proton decoupling. Chemical shifts are reported in ppm from TMS with the solvent as the internal standard (deuterochloroform: 77.0 ppm).

HRMS spectra were obtained with a G2XS QTof mass spectrometer using either ESI or APCI ionization techniques, as specified case by case.

Chromatographic purification was done with 240-400 mesh silica gel. Other anhydrous solvents were supplied by Sigma Aldrich in Sureseal® bottles and used without any further purification. Commercially available chemicals were purchased from Sigma Aldrich, Stream and TCI and used without any further purification. Melting points were determined with Bibby Stuart Scientific Melting Point Apparatus SMP 3 and are not corrected.

Anhydrous DMF was purchased from Merck and used as received. All other commercially available starting materials and (non-anhydrous) solvents were purchased from Merck, TCI chemicals, Fluorochem or Alfa Aesar and were used as such without further purification.

MBH acetates **1** are known compounds and were synthesized according to literature procedures.[1]

RAEs **2** are known compounds and were synthesized according to literature procedures.[2] For characterization see: **2a**,[2] **2b**,[3] **2c**,[2] **2d**,[2] **2e**,[4] **2f**,[3] **2g**,[4] **2h**,[5] **2i**,[2] **2j**.[2]

Table S1. Optimization of reaction conditions, additional data.

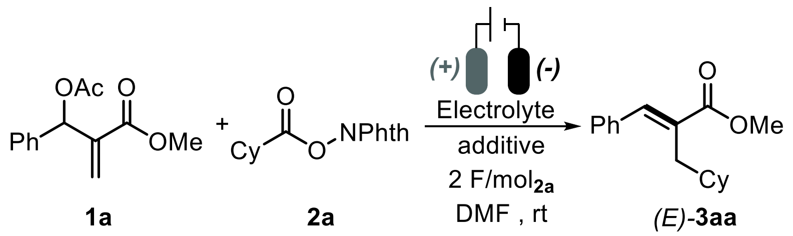

Entry	Electrolyte (equiv)	Anode (+) || Cathode (-)	Solvent	Additive (equiv)	I [mA]	Yield [%]
1	$TEABF_4$ (2)	Zn(+) || C(-)	ACN	none	4	45
2	$TEABF_4$ (2)	Zn(+) || C(-)	DMA	none	4	63
3	$LiPF_6$ (2)	Zn(+) || C(-)	DMF	none	4	59
4	TBAI (2)	Zn(+) || C(-)	DMF	none	4	58
5	$TBABF_4$ (2)	Zn(+) || C(-)	DMF	none	4	67
6	$TEABF_4$ (2)	Zn(+) || C(-)	DMF	PPh_3 (1)	4	23
7	$TEABF_4$ (2)	Zn(+) || C (-)	DMF	PPh_3 (0.2)	4	70
8	$TEABF_4$ (2)	Zn(+) || C(-)	DMF	PPh_3 (0.2)	2	78
9	$TEABF_4$ (2)	Zn(+) || Pt(-)	DMF	none	4	50
10	$TEABF_4$ (2)	Zn(+) || RVC foam(-)	DMF	none	4	63

Reaction conditions: **1a** (0.15 mmol), **2a** (0.30 mmol), electrolyte (0.30 mmol), dry DMF (3 mL). CCE (10, 4 or 2 mA; 2 F/mol$_{2a}$), rt.

In entries 6, 7, and 8 the use of PPh_3 as an additive was explored. Unfortunately, this did not result in a regioselectivity switch (S_N2 instead of S_N2' adduct, see **Scheme S1**) as it was postulated. The use of PPh_3 in stoichiometric amount resulted in a lower yield of isolated product **3aa** due to decomposition of **1a** (entry 6). Nonetheless, its presence, in catalytic amount, improved the yield slightly when the reaction was carried out at 4 mA, with complete consumption of **1a** (compare entry 7 with entry 4 of Table 1, main text). However, when the reaction was repeated at 2 mA, a slightly lower yield was recorded if compared to the reaction run in the absence of PPh_3 (compare entry 8 with entry 5 of Table 1, main text).

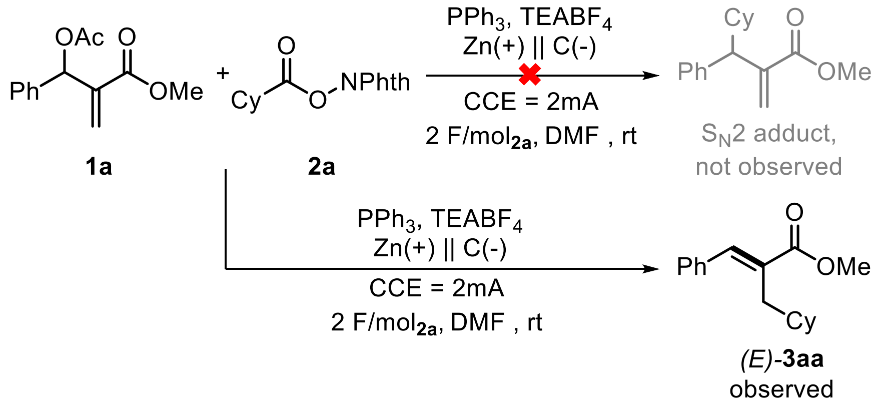

Scheme S1. Possible product distribution when PPh_3 is employed as an additive in the eChem alkylation of MBH adducts.

Limitation of the methodology: unsuccessful substrates.

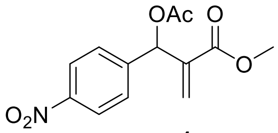

1p

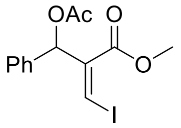

1q

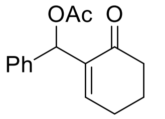

1r

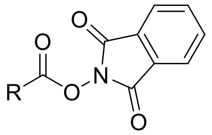

2k, R = Ph
2l, R = Bn
2m, R = allyl
2n, R = cinnamyl

Figure S1. Unsuccessful substrates.

MBH acetates **1p-1r** were tested under the optimal reaction conditions (RAE **2a**) but failed to give the desired products. A complex reaction mixture was observed by ^{1}H NMR spectroscopy when compound **1p** was employed. Successive de-iodination, reduction, radical alkylation sequences were observed when **1q** was subjected to the reaction mixture (compounds **1a**, **3aa** and **1a'** were observed). No reaction occurred with cyclohexanone-derived compound **1r**.

RAEs **2k-2n** were tested under the optimal reaction conditions (MBH acetate **1a**) but failed to give the desired products. In all cases, decomposition of RAE **2** was observed but no productive trapping of the radical by **1a** occurred. Substantial amounts of **1a'** and recovered **1a** were observed.

Cyclovoltammetry experiments

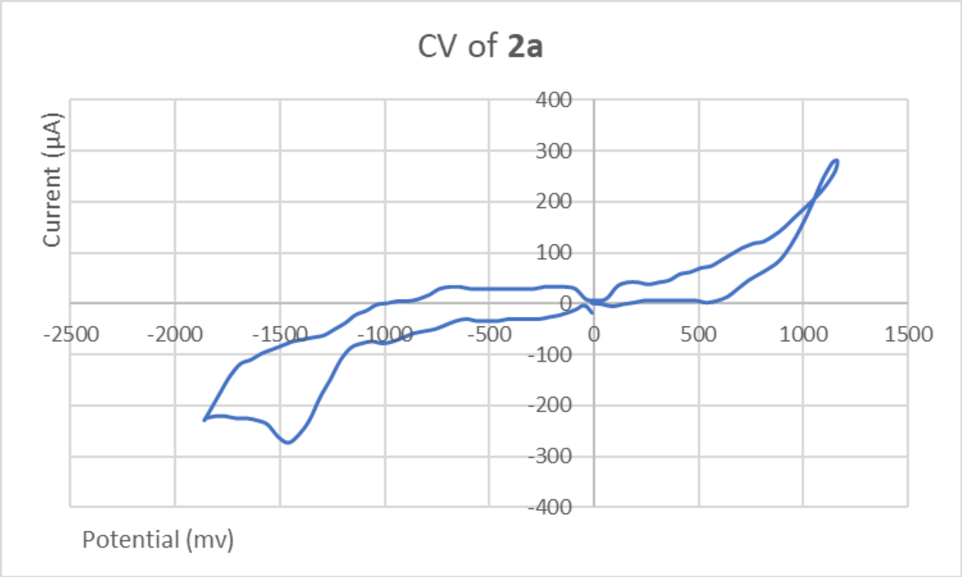

Figure S2. Cyclovoltammetry of RAE **2a**.

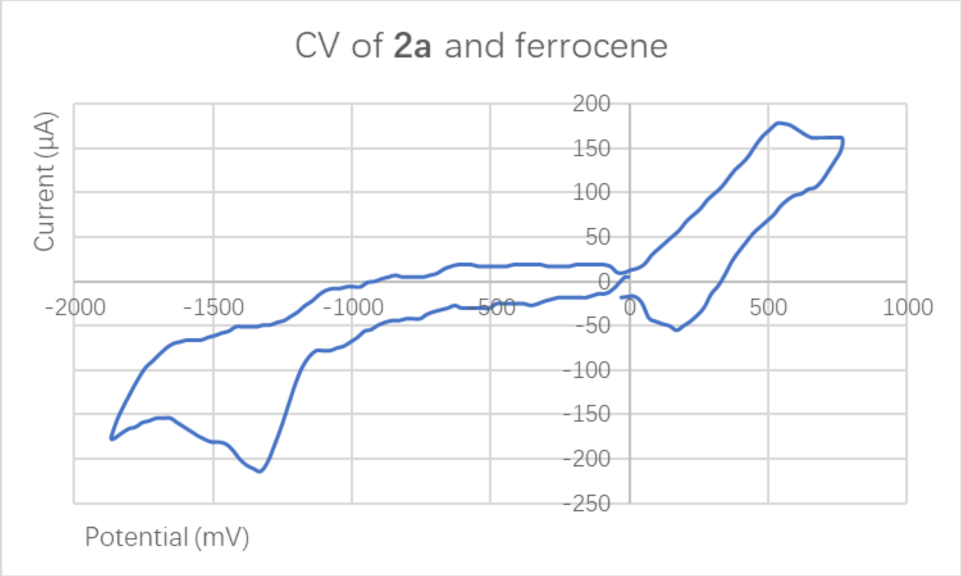

Figure S3. Cyclovoltammetry of RAE **2a** and ferrocene.

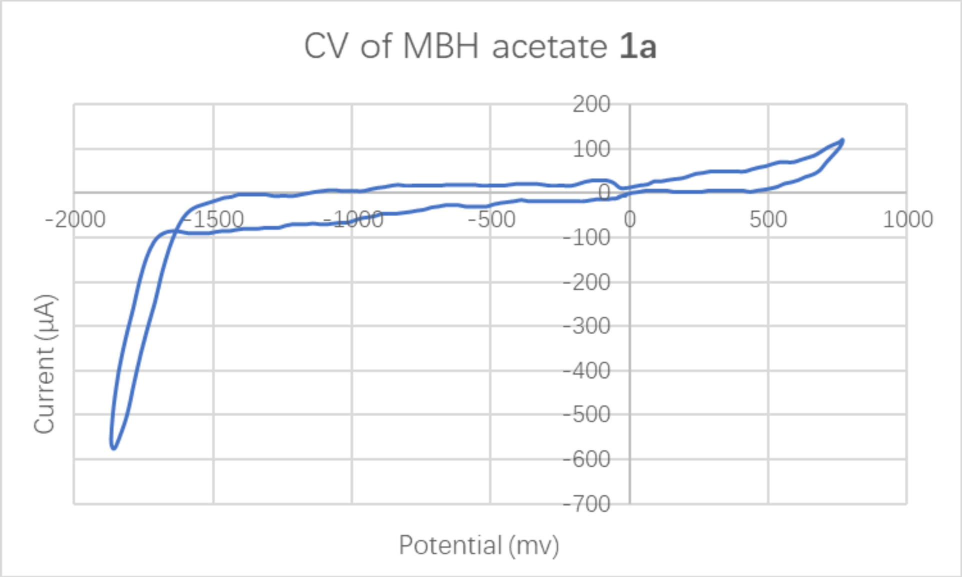

Figure S4. Cyclovoltammetry of MBH acetate **1a**.

Cyclovoltammetry experiments were carried out using the ElectraSyn 2.0 apparatus with a Pt counter electrode, an RVC working electrode and an Ag/Ag^+ reference electrode (Ag wire in a 10 mM $AgNO_3$ and 0.1 M $TBAClO_4$ acetonitrile solution).

For **2a**: 4.8 mg of **2a** and 100 mg $TBAPF_6$ were dissolved in 3 mL of anhydrous DMF, stirred while purging with N_2 (balloon) and then subjected to the CV experiment at 350 $mV{\cdot}s^{-1}$ (**Figure S2**). Then, 3.0 mg of ferrocene were added and the CV experiment was repeated (**Figure S3**). An irreversible peak between -1.27 and -1.49 V is observed. This is then calculated to be -1.57 V *vs* ferrocene (reversible peak between 0.072 and 0.325 V).

For **1a**: 3.7 mg of **2a** and 100 mg $TBAPF_6$ were dissolved in 3 mL of anhydrous DMF, stirred while purging with N_2 (balloon) and then subjected to the CV experiment at 350 $mV{\cdot}s^{-1}$ (**Figure S4**) No cathodic event is observed apart from reduction of the solvent occurring below -1.50 V.

.

Photoisomerization of ***E*-3aa**

In a flame-dried Schlenk tube equipped with a magnetic stirring bar, ***E*-3aa** (0.10 mmol, 25.8 mg) and Ir[dF(CF_3)ppy]$_2$(dtbppy)PF_6 (1 mg) were dissolved in dry NMP (1 mL) under N_2 atmosphere. The Schlenk flask was then sealed, and the content was stirred vigorously for 18 h under blue-light irradiation (20 W Kessil lamp, 427 nm, at a distance of approximately 6 cm). Then, EtOAc (5 mL) and $HCl_{(aq)}$ (1M, 5 mL) were added and the biphasic crude mixture was placed in a separatory funnel. The organic layer was separated, the aqueous layer was extracted with EtOAc (2 x 10 mL) and the combined organic layers were washed with $HCl_{(aq)}$ (0.1 M, 3 x 10 mL), dried over Na_2SO_4 and concentrated *in vacuo*. ^{1}H NMR of the crude mixture revealed that isomerization of the double bond occurred showing a ***Z*-3aa**:***E*-3aa** ratio = 1.7:1.

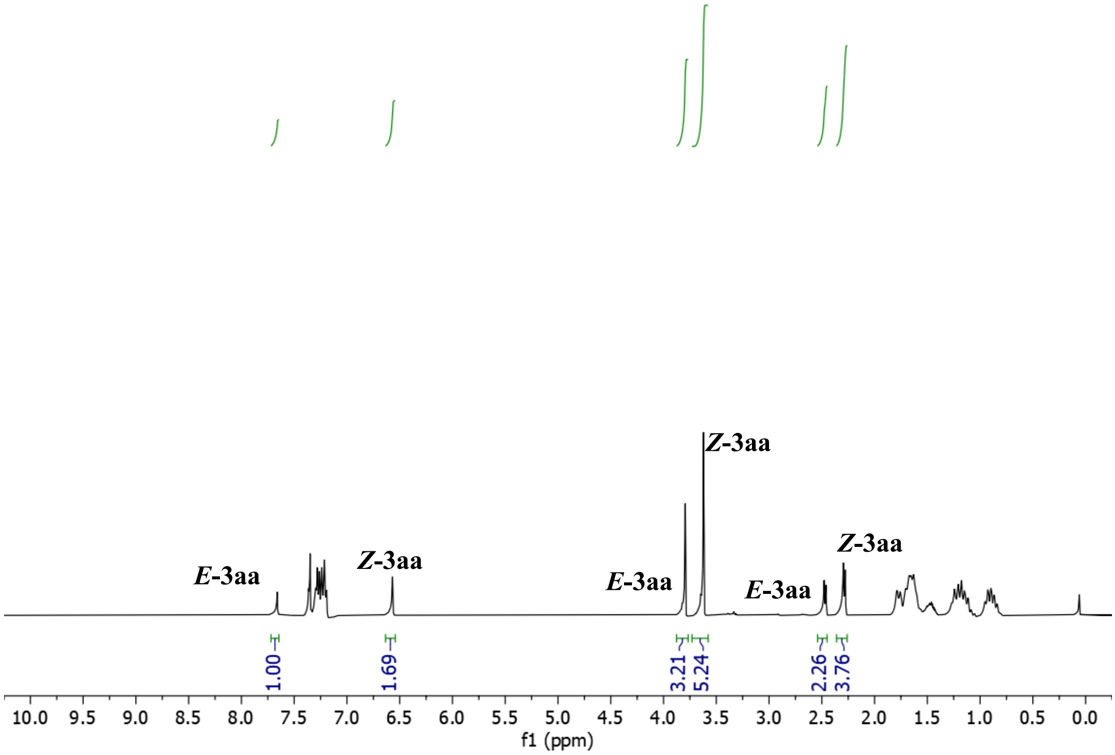

Figure S5. ^{1}H NMR of ***E*-3aa** and ***Z*-3aa** (1:1.7 mixture)

Optimized general procedure for the eChem alkylation of MBH adducts.

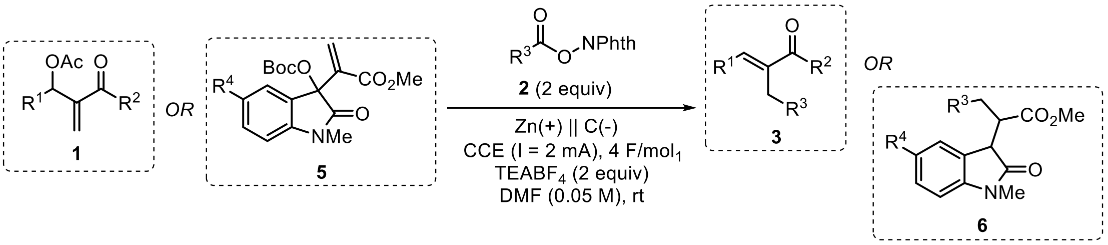

The ElectraSyn vial (5 mL), equipped with a stir bar, was charged with MBH acetate **1** or **5** (0.15 mmol), RAE **2** (0.30 mmol) and $TEABF_4$ (65.1 mg, 0.30 mmol). The ElectraSyn vial cap, equipped with anode (Zn) and cathode (graphite) was inserted into the mixture and closed with a rubber septum. The vessel was evacuated and backfilled with N_2 three times, then dry DMF (3 mL) was added, and the solution bubbled with N_2 (balloon) under stirring for 1 min. The reaction mixture was electrolyzed (under N_2, balloon) at a constant current of 2 mA, until a total charge of 0.60 F (4 F/mol_1) was reached (ca. 8 h). The ElectraSyn vial cap was removed, and the electrodes and vial were rinsed with EtOAc (10 mL) and $HCl_{(aq)}$ (1M, 10 mL), which were combined with the crude mixture in a separatory funnel. Then, the organic layer was separated, the aqueous layer was extracted with EtOAc (2 x 10 mL) and the combined organic layers were washed with $HCl_{(aq)}$ (0.1 M, 3 x 10 mL), dried over Na_2SO_4 and concentrated *in vacuo*. The crude product was finally purified by flash chromatography (*n*Hex/Et_2O mixtures) to afford pure products **3** or **6**.

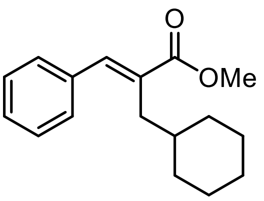

3aa. Viscous colorless oil. FC eluent: *n*Hex/Et_2O: 25:1. Yield = 79%, (0.119 mmol, 30.6 mg), *E*/*Z* ratio: >20:1. **^{1}H NMR** (400 MHz, $CDCl_3$) δ = 7.66 (s, 1H), 7.41 – 7.33 (m, 4H), 7.32 – 7.26 (m, 1H), 3.80 (s, 3H), 2.47 (d, *J* = 7.1 Hz, 2H), 1.71 – 1.42 (m, 6H), 1.28 – 1.00 (m, 3H), 0.93 – 0.78 (m, 2H); **^{13}C NMR** (100 MHz, $CDCl_3$) δ = 169.4, 139.4, 136.0, 132.7, 129.3 (2C), 128.4 (2C), 128.1, 52.0, 37.8, 34.4, 33.2 (2C), 26.4, 26.3 (2C); **HRMSI (APCI)** m/z: $[M+H]^+$ calcd. for $C_{17}H_{23}O_2$ 259.1693; found 259.1698.

Example of mmol scale synthesis of 3aa. The ElectraSyn vial (10 mL), equipped with a stir bar, was charged with MBH acetate **1a** (234 mg, 1.0 mmol), RAE **2a** (546 mg, 2.0 mmol) and $TEABF_4$ (434 mg, 2.0 mmol). The ElectraSyn vial cap, equipped with anode (Zn) and cathode (graphite) was inserted into the mixture and closed with a rubber septum. The vessel was evacuated and backfilled with N_2 three times, then dry DMF (9 mL) was added, and the solution bubbled with N_2 (balloon) under stirring for 1 min. The reaction mixture was electrolyzed (under N_2, balloon) at a constant current of 3 mA, until a total charge of 4.0 F (4 F/mol_{1a}) was reached (ca. 36 h). The ElectraSyn vial cap was removed, and the electrodes and vial were rinsed with EtOAc (25 mL) and $HCl_{(aq)}$ (1M, 25 mL), which were combined with the crude mixture in a separatory funnel. Then, the organic layer was separated, the aqueous layer was extracted with EtOAc (2 x 25 mL) and the combined organic layers were washed with $HCl_{(aq)}$ (0.1 M, 3 x 25 mL), dried over Na_2SO_4 and concentrated *in vacuo*. The crude product was finally purified by flash chromatography (*n*Hex/Et_2O mixtures) to afford pure products **3aa** in 83% yield (0.83 mmol, 244 mg).

3aa is a known compound and the reported spectroscopic data match with the ones reported in the literature (*E/Z* ratio: 1:1.7).[6]

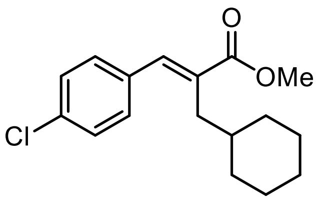

3ba. Viscous colorless oil. FC eluent: *n*Hex/Et_2O: 25:1. Yield = 76%, (0.114 mmol, 33.3 mg), *E*/*Z* ratio: >20:1. **^{1}H NMR** (400 MHz, $CDCl_3$) δ = 7.59 (s, 1H), 7.35 – 7.30 (m, 2H), 7.29 – 7.25 (m, 2H), 3.79 (s, 3H), 2.43 (d, *J* = 7.1 Hz, 2H), 1.71 – 1.41 (m, 6H), 1.25 – 0.99 (m, 3H), 0.92 – 0.74 (m, 2H); **^{13}C NMR** (100 MHz, $CDCl_3$) δ = 169.1, 138.0, 134.4, 134.0, 133.3, 130.5 (2C), 128.6 (2C), 52.0, 37.8, 34.4, 33.2 (2C), 26.3, 26.2 (2C); **HRMS (ESI)** m/z: $[M+H]^+$ calcd. for $C_{17}H_{22}{}^{35}ClO_2$ 293.1303; found 293.1303; calcd. for $C_{17}H_{22}{}^{37}ClO_2$, 295.1274; found 295.1275.

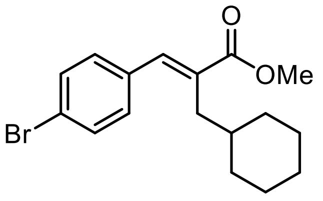

3ca. Viscous colorless oil. FC eluent: *n*Hex/Et_2O: 25:1. Yield = 70%, (0.105 mmol, 35.4 mg), *E*/*Z* ratio: >20:1. **^{1}H NMR** (400 MHz, $CDCl_3$) δ = 7.57 (s, 1H), 7.52 – 7.40 (m, 2H), 7.21 – 7.15 (m, 2H), 3.79 (s, 3H), 2.42 (d, *J* = 7.1 Hz, 2H), 1.66 – 1.41 (m, 6H), 1.19 – 1.02 (m, 3H), 0.90 – 0.74 (m, 2H); **^{13}C NMR** (100 MHz, $CDCl_3$) δ = 169.1, 138.1, 137.5, 134.9, 133.4, 131.6 (2C), 130.8 (2C), 52.0, 37.8, 34.4, 33.2 (2C), 26.3, 26.2 (2C); **HRMS (APCI)** m/z: $[M+H]^+$ calcd. for $C_{17}H_{22}{}^{79}BrO_2$ 337.0798; found 337.0792; calcd. for $C_{17}H_{22}{}^{81}BrO_2$ 339.0778; found 339.0783.

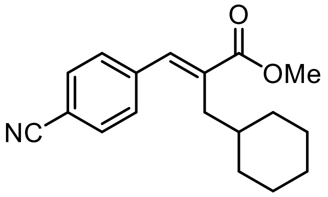

3da. Viscous colorless oil. FC eluent: *n*Hex/Et_2O: 10:1. Yield = 61%, (0.092 mmol, 25.9 mg), *E*/*Z* ratio: >20:1. **^{1}H NMR** (400 MHz, $CDCl_3$) δ = 7.69 – 7.63 (m, 2H), 7.61 (s, 1H), 7.43 – 7.37 (m, 2H), 3.81 (s, 3H), 2.40 (d, *J* = 7.1 Hz, 2H), 1.67 – 1.52 (m, 5H), 1.53 – 1.39 (m, 1H), 1.27 – 0.98 (m, 3H), 0.86 – 0.71 (m, 2H); **^{13}C NMR** (100 MHz, $CDCl_3$) δ = 168.6, 140.8, 137.1, 135.6, 132.2 (2C), 129.6 (2C), 118.6, 111.5, 52.2, 37.7, 34.6, 33.2 (2C), 26.2, 26.2 (2C); **HRMS (APCI)** m/z: $[M+H]^+$ calcd. for $C_{18}H_{22}NO_2$ 284.1645; found 284.1653.

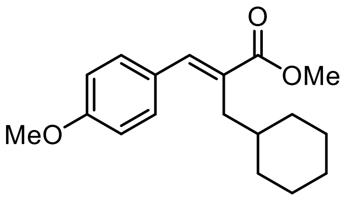

3ea. Viscous colorless oil. FC eluent: *n*Hex/Et_2O: 15:1. Yield = 67%, (0.101 mmol, 28.9 mg), *E*/*Z* ratio: >20:1. **^{1}H NMR** (400 MHz, $CDCl_3$) δ = 7.60 (s, 1H), 7.39 – 7.30 (m, 2H), 6.94 – 6.85 (m, 2H), 3.82 (s, 3H), 3.78 (s, 3H), 2.49 (d, *J* = 7.1 Hz, 2H), 1.72 – 1.45 (m, 6H), 1.26 – 1.05 (m, 3H), 0.98 – 0.85 (m, 2H); **^{13}C NMR** (100 MHz, $CDCl_3$) δ = 169.7, 159.6, 139.0, 131.1 (2C), 130.6, 128.4, 113.9 (2C), 55.2, 51.8, 37.9, 34.4, 33.3 (2C), 26.4, 26.3 (2C); **HRMS (APCI)** m/z: $[M+H]^+$ calcd. for $C_{18}H_{25}O_3$ 289.1798; found 289.1805.

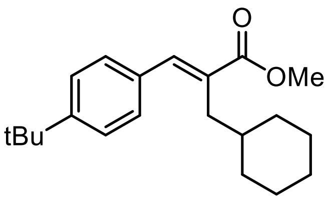

3fa. Viscous colorless oil. FC eluent: *n*Hex/Et_2O: 30:1. Yield = 61%, (0.092 mmol, 28.7 mg), *E*/*Z* ratio: >20:1. **^{1}H NMR** (400 MHz, $CDCl_3$) δ = 7.63 (s, 1H), 7.42 – 7.37 (m, 2H), 7.36 – 7.30 (m, 2H), 3.79 (s, 3H), 2.51 (d, *J* = 7.1 Hz, 2H), 1.72 – 1.47 (m, 6H), 1.32 (s, 9H), 1.23 – 1.07 (m, 3H), 1.01 – 0.86 (m, 2H); **^{13}C NMR** (100 MHz, $CDCl_3$) δ = 169.6, 151.5, 139.2, 133.0, 131.8, 129.4 (2C), 125.4 (2C), 51.9, 37.9, 34.7, 34.5, 33.3 (2C), 31.2 (3C), 26.4, 26.3 (2C); **HRMS (APCI)** m/z: $[M+H]^+$ calcd. for $C_{21}H_{31}O_2$ 315.2319; found 315.2326.

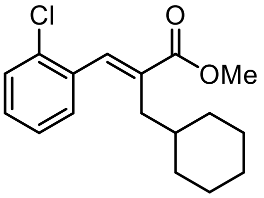

3ga. Viscous colorless oil. FC eluent: *n*Hex/Et_2O: 25:1. Yield = 77%, (0.115 mmol, 33.9 mg), *E*/*Z* ratio: >20:1. **^{1}H NMR** (400 MHz, $CDCl_3$) δ = 7.68 (s, 1H), 7.43 – 7.35 (m, 1H), 7.28 – 7.23 (m, 3H), 3.81 (s, 3H), 2.30 (d, *J* = 7.1 Hz, 2H), 1.65 – 1.51 (m, 5H), 1.50 – 1.37 (m, 1H), 1.19 – 0.93 (m, 3H), 0.76 – 0.62 (m, 2H); **^{13}C NMR** (100 MHz, $CDCl_3$) δ = 168.6, 136.9, 134.9, 134.3, 133.8, 130.1, 129.5, 129.1, 126.4, 52.0, 37.3, 34.6, 33.0 (2C), 26.3, 26.2 (2C); **HRMS (APCI)** m/z: $[M+H]^+$ calcd. for $C_{17}H_{22}{}^{35}ClO_2$ 293.1303; found, 293.1309; calcd. for $C_{17}H_{22}{}^{37}ClO_2$ 295.1274; found 295.1286.

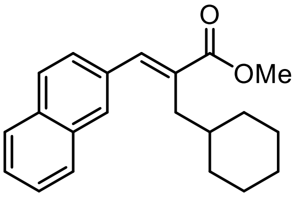

3ha. Viscous colorless oil. FC eluent: *n*Hex/Et_2O: 25:1. Yield = 70%, (0.105 mmol, 32.4 mg), *E*/*Z* ratio: >20:1. **^{1}H NMR** (400 MHz, $CDCl_3$) δ = 7.87 – 7.80 (m, 5H), 7.53 – 7.46 (m, 3H), 3.83 (s, 3H), 2.57 (d, *J* = 7.1 Hz, 2H), 1.74 – 1.50 (m, 6H), 1.27 – 1.02 (m, 3H), 0.94 – 0.80 (m, 2H); **^{13}C NMR** (100 MHz, $CDCl_3$) δ = 169.4, 139.4, 133.5, 133.1, 132.9, 132.8, 129.0, 128.3, 128.0, 127.6, 126.8, 126.6, 126.4, 52.0, 37.9, 34.6, 33.3 (2C), 26.4, 26.3 (2C); **HRMS (APCI)** m/z: $[M+H]^+$ calcd. for $C_{21}H_{25}O_2$ 309.1849; found 309.1849.

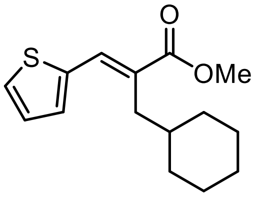

3ia. Viscous colorless oil. FC eluent: *n*Hex/Et_2O: 25:1. Yield = 71%, (0.107 mmol, 28.1 mg), *E*/*Z* ratio: >20:1. **^{1}H NMR** (400 MHz, $CDCl_3$) δ = 7.83 (s, 1H), 7.43 (dt, *J* = 5.1, 1.1 Hz, 1H), 7.27 – 7.21 (m, 1H), 7.06 (dd, *J* = 5.2, 3.7 Hz, 1H), 3.78 (s, 3H), 2.61 (d, *J* = 7.2 Hz, 2H), 1.77 – 1.52 (m, 7H), 1.27 – 0.96 (m, 4H); **^{13}C NMR** (100 MHz, $CDCl_3$) δ = 169.3, 138.7, 132.5, 132.0, 128.8, 128.8, 127.1, 51.9, 37.8, 35.2, 33.3 (2C), 26.4, 26.4 (2C); **HRMS (APCI)** m/z: $[M+H]^+$ calcd. for $C_{15}H_{21}O_2S$ 265.1257; found 265.1260.

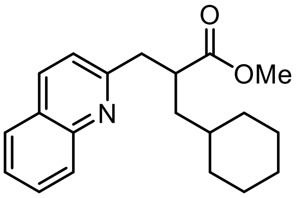

3ja'. Viscous colorless oil. FC eluent: *n*Hex/EtOAc: 10:1. Yield = 64%, (0.096 mmol, 29.9 mg), clean reduction of the double bond occurred, **3ia** not detected. **^{1}H NMR** (400 MHz, $CDCl_3$) δ = 8.05 – 7.95 (m, 2H), 7.75 (dd, *J* = 8.0, 1.4 Hz, 1H), 7.65 (ddd, *J* = 8.5, 6.9, 1.5 Hz, 1H), 7.46 (ddd, *J* = 8.1, 6.9, 1.2 Hz, 1H), 7.23 (d, *J* = 8.7 Hz, 1H), 3.59 (s, 3H), 3.27 (dd, *J* = 13.5, 8.8 Hz, 1H), 3.19 (tt, *J* = 8.9, 5.2 Hz, 1H), 3.06 (dd, *J* = 13.5, 5.4 Hz, 1H), 1.85 – 1.76 (m, 1H), 1.74 – 1.56 (m, 5H), 1.39 (ddd, *J* = 13.5, 8.3, 5.2 Hz, 1H), 1.32 – 1.03 (m, 3H), 0.94 – 0.74 (m, 2H); **^{13}C NMR** (100 MHz, $CDCl_3$) δ = 176.6, 159.9, 147.9, 136.1, 129.3, 129.0, 127.4, 126.8, 125.8, 121.6, 51.4, 42.8, 41.6, 40.2, 35.6, 33.6, 32.8, 26.5, 26.2, 26.2; **HRMS (APCI)** m/z: $[M+H]^+$ calcd. for $C_{20}H_{26}NO_2$ 312.1958; found 312.1963.

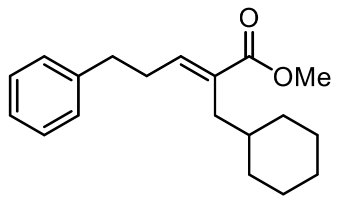

3ka. Viscous colorless oil. FC eluent: *n*Hex/Et_2O: 25:1. Yield = 62%, (0.093 mmol, 28.8 mg), *E*/*Z* ratio: >20:1. **^{1}H NMR** (400 MHz, $CDCl_3$) δ = 7.32 – 7.26 (m, 2H), 7.22 – 7.14 (m, 3H), 6.82 (t, *J* = 7.4 Hz, 1H), 3.71 (s, 3H), 2.73 (dd, *J* = 9.0, 6.7 Hz, 2H), 2.48 (q, *J* = 7.6 Hz, 2H), 2.16 (d, *J* = 7.1 Hz, 2H), 1.70 – 1.57 (m, 5H), 1.43 – 1.27 (m, 1H), 1.27 – 1.02 (m, 3H), 0.93 – 0.79 (m, 2H); **^{13}C NMR** (100 MHz, $CDCl_3$) δ = 168.6, 142.0, 141.2, 131.5, 128.4 (2C), 128.3 (2C), 126.1, 51.6, 37.7, 35.0, 34.3, 33.2 (2C), 30.9, 26.4, 26.3 (2C); **HRMS (APCI)** m/z: $[M+H]^+$ calcd. for $C_{19}H_{27}O_2$ 287.2006; found: 287.2014.

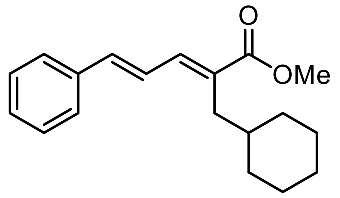

3la. Pale yellow solid. FC eluent: *n*Hex/Et_2O: 25:1. Yield = 57%, (0.086 mmol, 24.3 mg), *E*/*Z* ratio: >20:1. **MP**: 106 - 108 °C. **^{1}H NMR** (400 MHz, $CDCl_3$) δ = 7.47 – 7.43 (m, 2H), 7.40 (d, *J* = 11.3 Hz, 1H), 7.37 – 7.32 (m, 2H), 7.31 – 7.25 (m, 1H), 7.03 (dd, *J* = 15.4, 11.3 Hz, 1H), 6.85 (d, *J* = 15.4 Hz, 1H), 3.76 (s, 3H), 2.39 (d, *J* = 7.0 Hz, 2H), 1.67 (ddd, *J* = 21.5, 16.4, 9.7 Hz, 5H), 1.55 – 1.38 (m, 1H), 1.27 – 1.09 (m, 3H), 1.02 – 0.88 (m, 2H); **^{13}C NMR** (100 MHz, $CDCl_3$) δ = 168.8, 139.3, 139.1, 136.6, 130.9, 128.8 (2C), 128.6, 127.0 (2C), 124.2, 51.7, 38.3, 34.7, 33.4 (2C), 26.5, 26.3 (2C); **HRMS (ESI)** m/z: $[M+H]^+$ calcd. for $C_{19}H_{25}O_2$ 285.1849; found 285.1852.

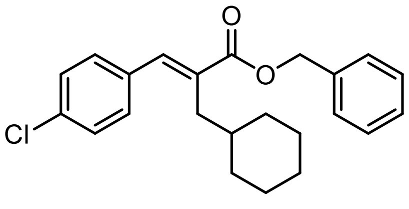

3ma. Viscous colorless oil. FC eluent: *n*Hex/Et_2O: 25:1. Yield = 76%, (0.114 mmol, 42.0 mg), *E*/*Z* ratio: >20:1. **^{1}H NMR** (400 MHz, $CDCl_3$) δ = 7.63 (s, 1H), 7.43 – 7.36 (m, 4H), 7.36 – 7.30 (m, 3H), 7.29 – 7.25 (m, 2H), 5.24 (s, 2H), 2.45 (d, *J* = 7.1 Hz, 2H), 1.70 – 1.54 (m, 5H), 1.54 – 1.44 (m, 1H), 1.19 – 1.01 (m, 3H), 0.90 – 0.76 (m, 2H); **^{13}C NMR** (100 MHz, $CDCl_3$) δ = 168.4, 138.2, 136.1, 134.4, 134.0, 133.4, 130.5 (2C), 128.6 (2C), 128.5 (2C), 128.2, 128.1 (2C), 66.6, 37.8, 34.5, 33.2 (2C), 26.3, 26.2 (2C); **HRMS (APCI)** m/z: $[M+H]^+$ calcd. for $C_{23}H_{26}{}^{35}ClO_2$ 369.1616; found 369.1617; calcd. for $C_{23}H_{26}{}^{37}ClO_2$ 371.1587; found 371.1596.

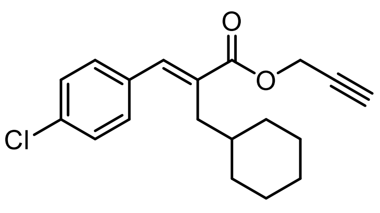

3na. Viscous colorless oil. FC eluent: *n*Hex/Et_2O: 25:1. Yield = 74%, (0.111 mmol, 35.1 mg), *E*/*Z* ratio: >20:1. **^{1}H NMR** (400 MHz, $CDCl_3$) δ = 7.64 (s, 1H), 7.37 – 7.32 (m, 2H), 7.30 – 7.26 (m, 2H), 4.80 (d, *J* = 2.4 Hz, 2H), 2.48 (t, *J* = 2.4 Hz, 1H), 2.45 (d, *J* = 7.1 Hz, 2H), 1.68 – 1.54 (m, 5H), 1.54 – 1.44 (m, 1H), 1.22 – 1.02 (m, 3H), 0.91 – 0.77 (m, 2H); **^{13}C NMR** (100 MHz, $CDCl_3$) δ = 167.7, 139.0, 134.2, 134.2, 132.7, 130.6 (2C), 128.7 (2C), 77.8, 74.8, 52.3, 37.8, 34.4, 33.2 (2C), 26.3, 26.2 (2C); **HRMS (APCI)** m/z: $[M+H]^+$ calcd. for $C_{19}H_{22}{}^{35}ClO_2$ 317.1303; found 317.1309; calcd. for $C_{19}H_{22}{}^{37}ClO_2$ 319.1274; found 319.1285.

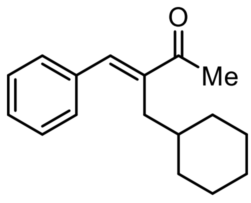

3oa. Viscous colorless oil. FC eluent: *n*Hex/Et_2O: 15:1. Yield = 73%, (0.110 mmol, 26.5 mg), *E*/*Z* ratio: >20:1. **^{1}H NMR** (400 MHz, $CDCl_3$) δ = 7.47 (s, 1H), 7.42 – 7.35 (m, 4H), 7.34 – 7.28 (m, 1H), 2.46 (d, *J* = 7.1 Hz, 2H), 2.43 (s, 3H), 1.66 – 1.52 (m, 5H), 1.50 – 1.35 (m, 1H), 1.20 – 0.99 (m, 3H), 0.91 – 0.76 (m, 2H); **^{13}C NMR** (100 MHz, $CDCl_3$) δ = 200.8, 142.3, 139.7, 136.0, 129.2 (2C), 128.5 (2C), 128.3, 37.6, 33.3 (2C), 33.2, 26.3, 26.3, 26.3 (2C); **HRMS (APCI)** m/z: $[M+H]^+$ calcd. for $C_{17}H_{23}O$ 243.1743; found 243.1743.

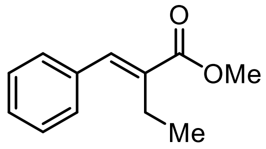

3ab. Viscous colorless oil. FC eluent: *n*Hex/Et_2O: 25:1. Yield = 49%, (0.074 mmol, 14.0 mg), *E*/*Z* ratio: >20:1. **^{1}H NMR** (400 MHz, $CDCl_3$) δ = 7.64 (s, 1H), 7.41 – 7.27 (m, 5H), 3.81 (s, 3H), 2.53 (q, *J* = 7.4 Hz, 2H), 1.16 (t, *J* = 7.4 Hz, 3H); **^{13}C NMR** (100 MHz, $CDCl_3$) δ = 168.8, 138.6, 135.7, 134.7, 129.2 (2C), 128.4 (2C), 128.3, 51.9, 20.8, 13.9; **HRMS (APCI)** m/z: $[M+H]^+$ calcd. for $C_{12}H_{15}O_2$ 191.1067; found 191.1073.

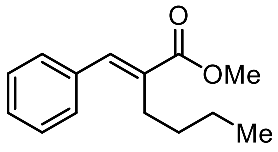

3ac. Viscous colorless oil. FC eluent: *n*Hex/Et_2O: 25:1. Yield = 55%, (0.083 mmol, 18.0 mg), *E*/*Z* ratio: >20:1. **^{1}H NMR** (400 MHz, $CDCl_3$) δ = 7.64 (s, 1H), 7.41 – 7.28 (m, 5H), 3.80 (s, 3H), 2.55 – 2.46 (m, 2H), 1.55 – 1.46 (m, 2H), 1.42 – 1.31 (m, 2H), 0.90 (t, *J* = 7.3 Hz, 3H); **^{13}C NMR** (100 MHz, $CDCl_3$) δ = 169.0, 138.7, 135.8, 133.6, 129.2 (2C), 128.4 (2C), 128.3, 51.9, 31.4, 27.3, 22.8, 13.8; **HRMS (APCI)** m/z: $[M+H]^+$ calcd. for $C_{14}H_{19}O_2$ 219.1380; found: 219.1379.

3ac is a known compound and the reported spectroscopic data match with the ones reported in the literature (*E/Z* ratio: 1:1.7).[6]

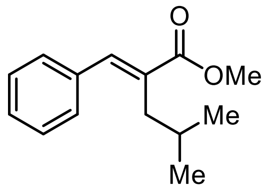

3ad. Viscous colorless oil. FC eluent: *n*Hex/Et_2O: 25:1. Yield = 69%, (0.104 mmol, 18.0 mg), *E*/*Z* ratio: >20:1. **^{1}H NMR** (400 MHz, $CDCl_3$) δ = 7.67 (s, 1H), 7.36 (d, *J* = 5.0 Hz, 4H), 7.31 – 7.25 (m, 1H), 3.79 (s, 3H), 2.47 (d, *J* = 7.2 Hz, 2H), 1.85 (sept, *J* = 6.9 Hz, 1H), 0.84 (d, *J* = 6.7 Hz, 6H); **^{13}C NMR** (100 MHz, $CDCl_3$) δ = 169.3, 139.4, 136.0, 133.1, 129.2 (2C), 128.4 (2C), 128.1, 51.9, 35.6, 28.2, 22.4 (2C); **HRMS (APCI)** m/z: $[M+H]^+$ calcd. for $C_{14}H_{19}O_2$ 219.1380; found 219.1389.

3ad is a known compound and the reported spectroscopic data match with the ones reported in the literature (*E/Z* ratio: 1:1.7).[6]

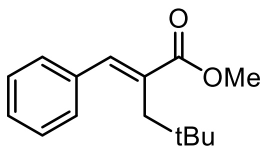

3ae. Viscous colorless oil. FC eluent: *n*Hex/Et_2O: 25:1. Yield = 80%, (0.120 mmol, 27.9 mg), *E*/*Z* ratio: >20:1. **^{1}H NMR** (400 MHz, $CDCl_3$) δ = 7.66 (s, 1H), 7.39 – 7.31 (m, 4H), 7.29 – 7.25 (m, 1H), 3.78 (s, 3H), 2.63 (s, 2H), 0.73 (s, 9H); **^{13}C NMR** (100 MHz, $CDCl_3$) δ = 170.3, 139.9, 136.6, 132.6, 128.9 (2C), 128.3 (2C), 127.7, 51.9, 38.2, 33.4, 29.5 (3C); **HRMSI (APCI)** m/z: $[M+H]^+$ calcd. for $C_{15}H_{21}O_2$ 233.1536; found 233.1545.

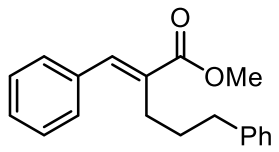

3af. Viscous colorless oil. FC eluent: *n*Hex/Et_2O: 25:1. Yield = 69%, (0.104 mmol, 29.2 mg), *E*/*Z* ratio: >20:1. ^{1}**H NMR** (400 MHz, $CDCl_3$) δ = 7.65 (s, 1H), 7.29 (dt, *J* = 4.4, 1.9 Hz, 4H), 7.27 – 7.22 (m, 2H), 7.22 – 7.15 (m, 4H), 3.81 (s, 3H), 2.68 (t, *J* = 7.4 Hz, 2H), 2.61 – 2.52 (m, 2H), 1.95 – 1.83 (m, 2H); ^{13}**C NMR** (100 MHz, $CDCl_3$) δ = 168.9, 141.9, 139.1, 135.6, 133.0, 129.2 (2C), 128.5 (2C), 128.4 (2C), 128.3, 128.3 (2C), 125.8, 52.0, 35.8, 30.6, 27.1; **HRMS (APCI)** m/z: $[M+H]^+$ calcd. for $C_{19}H_{21}O_2$ 281.1536; found 281.1544.

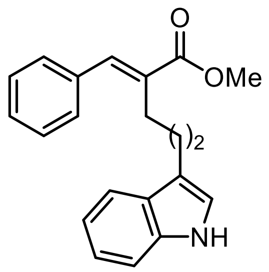

3ag. White solid. FC eluent: *n*Hex/EtOAc: 5:1. Yield = 61%, (0.092 mmol, 29.2 mg), *E*/*Z* ratio: >20:1. **MP**: 95 - 98 °C. ^{1}**H NMR** (400 MHz, $CDCl_3$) δ = 7.89 (bs, 1H), 7.62 (s, 1H), 7.60 (dd, *J* = 7.8, 1.2 Hz, 1H), 7.36 (dt, *J* = 8.1, 1.0 Hz, 1H), 7.23 – 7.08 (m, 7H), 6.93 (d, *J* = 2.3 Hz, 1H), 3.79 (d, *J* = 1.3 Hz, 3H), 2.84 (t, *J* = 7.3 Hz, 2H), 2.64 – 2.55 (m, 2H), 2.06 – 1.92 (m, 2H); ^{13}**C NMR** (100 MHz, $CDCl_3$) δ = ^{13}C NMR (101 MHz, cdcl3) δ 169.0, 138.9, 136.4, 135.6, 133.2, 129.2 (2C), 128.3 (2C), 128.2, 127.5, 121.9, 121.5, 119.2, 119.0, 116.0, 111.0, 51.9, 29.1, 27.3, 25.1; **HRMS (APCI)** m/z: $[M+H]^+$ calcd. for $C_{21}H_{22}NO_2$ 320.1645; found 320.1651.

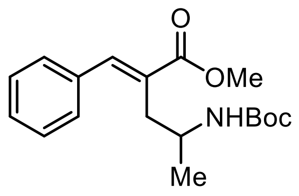

3ah. Viscous colorless oil. FC eluent: *n*Hex/EtOAc: 10:1. Yield = 67%, (0.101 mmol, 32.1 mg), *E*/*Z* ratio: >20:1. ^{1}**H NMR** (400 MHz, $CDCl_3$) δ = 7.75 (s, 1H), 7.41 – 7.26 (m, 5H), 4.50 (bs, 1H), 3.92 (bs, 1H), 3.82 (s, 3H), 2.73 – 2.56 (m, 2H), 1.38 (s, 9H), 1.09 (d, *J* = 6.6 Hz, 3H); ^{13}**C NMR** (100 MHz, $CDCl_3$) δ = 169.1, 155.2, 141.2, 135.6, 130.4, 129.1 (2C), 128.5 (2C), 128.4, 78.8, 52.1, 46.8, 34.4, 28.3 (3C), 21.6; **HRMS (ESI)** m/z: $[M+H]^+$ calcd. for $C_{18}H_{26}NO_4$ 320.1856; found: 320.1852.

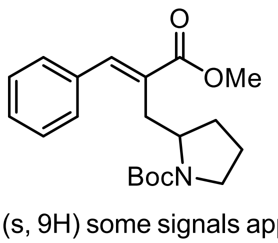

3ai. Pale yellow oil. FC eluent: *n*Hex/EtOAc: 10:1. Yield = 75%, (0.113 mmol, 38.9 mg), *E*/*Z* ratio: >20:1. ^{1}**H NMR** (400 MHz, CD_3OD) δ = 7.77 (s, 1H), 7.44 – 7.35 (m, 3H), 7.35 – 7.26 (m, 2H), 4.20 (dddd, *J* = 8.8, 7.1, 5.6, 1.3 Hz, 1H), 3.79 (s, 3H), 3.19 (q, *J* = 9.1 Hz, 1H), 2.93 – 2.80 (m, 2H), 2.53 (td, *J* = 16.8, 13.4, 7.2 Hz, 1H), 1.88 – 1.51 (m, 4H), 1.39 (s, 9H) some signals appear doubled due to the slow rotation of the C-N bond of the Boc moiety; ^{13}**C NMR** (100 MHz, CD_3OD) δ = 172.5, 159.0, 145.1, 139.6, 134.4, 132.4, 132.2 (2C), 132.0 (2C), 83.6, 60.3, 55.0, 49.2, 35.4, 34.4, 31.2 (3C), 25.8 some signals appear doubled due to the slow rotation of the C-N bond of the Boc moiety; **HRMS (ESI)** m/z: $[M+H]^+$ calcd. for $C_{20}H_{28}NO_4$ 346.2013; found 346.2009.

3ai is a known compound and the reported spectroscopic data match with the ones reported in the literature (*E/Z* ratio: 1:1).[6]

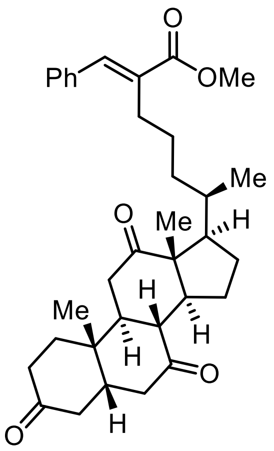

3aj. White solid. FC eluent: *n*Hex/EtOAc: 1:1. Yield = 66%, (0.099 mmol, 52.7 mg), *E*/*Z* ratio: >20:1. **MP**: 186 - 188 °C. **^{1}H NMR** (400 MHz, $CDCl_3$) δ = 7.62 (s, 1H), 7.40 – 7.26 (m, 5H), 3.78 (s, 3H), 2.93 – 2.76 (m, 3H), 2.57 – 2.36 (m, 2H), 2.37 – 2.04 (m, 7H), 2.04 – 1.87 (m, 4H), 1.81 (td, *J* = 11.1, 7.1 Hz, 1H), 1.73 – 1.53 (m, 3H), 1.51 – 1.38 (m, 1H), 1.37 (s, 3H), 1.35 – 1.08 (m, 5H), 1.02 (s, 3H), 0.81 (d, *J* = 6.4 Hz, 3H); **^{13}C NMR** (100 MHz, $CDCl_3$) δ = 212.0, 209.0, 208.7, 168.9, 138.7, 135.8, 133.6, 129.1 (2C), 128.4 (2C), 128.3, 56.9, 51.9, 51.8, 49.0, 46.8, 45.8, 45.5, 45.0, 42.8, 38.6, 36.5, 36.0, 35.7, 35.3, 35.2, 27.8, 27.7, 26.2, 25.2, 21.9, 18.9, 11.8; **HRMS (ESI)** m/z: $[M+H]^+$ calcd. for $C_{34}H_{45}O_5$ 533.3262; found 533.3257.

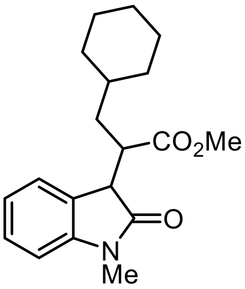

6aa. Colourless sticky foam. FC eluent: *n*Hex/EtOAc: 3:1. Yield = 57%, (0.086 mmol, 26.9 mg), *dr*: >20:1. **^{1}H NMR** (400 MHz, $CDCl_3$) δ = ^{1}H NMR (401 MHz, cdcl3) δ 7.31 – 7.20 (m, 2H), 7.01 (td, *J* = 7.5, 1.0 Hz, 1H), 6.80 (d, *J* = 7.8 Hz, 1H), 3.74 (d, *J* = 3.7 Hz, 1H), 3.71 (s, 3H), 3.29 (ddd, *J* = 9.6, 5.0, 3.7 Hz, 1H), 3.19 (s, 3H), 1.82 – 1.48 (m, 7H), 1.30 – 0.97 (m, 4H), 0.86 – 0.66 (m, 2H); **^{13}C NMR** (100 MHz, $CDCl_3$) δ =171.7, 170.5, 140.2, 123.9, 121.7, 120.4, 118.0, 103.6, 47.6, 42.7, 38.5, 31.2, 30.8, 29.1, 28.3, 26.7, 25.6, 22.1, 21.9; **HRMS (APCI)** m/z: $[M+H]^+$ calcd. for $C_{19}H_{26}NO_3$ 316.1907; found 316.1915.

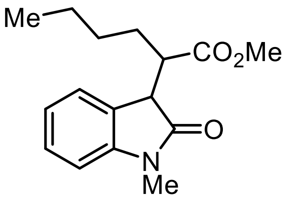

6ac. Pale yellow sticky foam. FC eluent: *n*Hex/EtOAc: 3:1. Yield = 51%, (0.077 mmol, 21.0 mg), *dr*: >20:1. **^{1}H NMR** (400 MHz, $CDCl_3$) δ = 7.31 – 7.21 (m, 2H), 7.01 (t, *J* = 7.5 Hz, 1H), 6.81 (d, *J* = 7.7 Hz, 1H), 3.77 (d, *J* = 3.8 Hz, 1H), 3.72 (s, 3H), 3.20 (s, 3H), 3.18 – 3.12 (m, 1H), 1.32 – 1.13 (m, 6H), 0.78 (t, *J* = 6.8 Hz, 3H); **^{13}C NMR** (100 MHz, $CDCl_3$) δ = 176.1, 174.6, 144.5, 128.2, 126.1, 124.8, 122.3, 108.0, 51.9, 46.9, 45.6, 29.8, 27.6, 26.2, 22.4, 13.8; **HRMS (APCI)** m/z: $[M+H]^+$ calcd. for $C_{16}H_{22}NO_3$ 276.1594; found 276.1595.

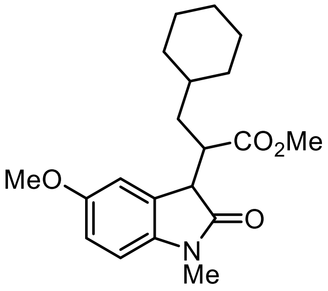

6ba. Pale yellow sticky foam. FC eluent: *n*Hex/EtOAc: 2:1. Yield = 62%, (0.093 mmol, 32.1 mg), *dr*: >20:1. **^{1}H NMR** (400 MHz, CDC_3) δ = 6.89 (dd, *J* = 2.5, 1.2 Hz, 1H), 6.79 (ddd, *J* = 8.4, 2.6, 0.8 Hz, 1H), 6.70 (d, *J* = 8.4 Hz, 1H), 3.77 (s, 3H), 3.71 (s, 3H) overlapped with 3.73 – 3.65 (m, 1H), 3.29 (ddd, *J* = 9.3, 5.0, 3.6 Hz, 1H), 3.17 (s, 3H), 1.73 – 1.51 (m, 7H), 1.21 – 0.95 (m, 4H), 0.88 – 0.66 (m, 2H); **^{13}C NMR** (100 MHz, $CDCl_3$) δ = 171.7, 170.5, 140.2, 123.9, 121.7, 120.4, 118.0, 103.6, 47.6, 42.7, 38.5, 31.2, 30.8, 29.1, 28.3, 26.7, 25.6, 22.1, 21.9; **HRMS (APCI)** m/z: $[M+H]^+$ calcd. for $C_{20}H_{28}NO_4$ 346.2013; found 346.2018.

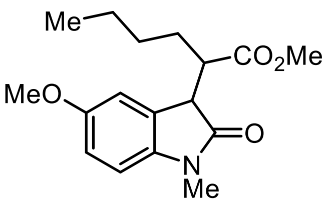

6bc. Pale yellow sticky foam. FC eluent: *n*Hex/EtOAc: 3:1. Yield = 66%, (0.099 mmol, 30.2 mg), *dr*: >20:1. **^{1}H NMR** (400 MHz, $CDCl_3$) δ = 6.91 (dd, *J* = 2.7, 1.3 Hz, 1H), 6.79 (dd, *J* = 8.5, 2.6 Hz, 1H), 6.70 (d, *J* = 8.3 Hz, 1H), 3.81 – 3.74 (m, 4H), 3.72 (s, 3H), 3.20 – 3.10 (m, 4H), 1.27 – 1.11 (m, 5H), 0.95 – 0.82 (m, 1H), 0.78 (t, *J* = 6.8 Hz, 3H); **^{13}C NMR** (100 MHz, $CDCl_3$) δ = ^{13}C NMR (101 MHz, $cdcl_3$) δ 175.8, 174.6, 155.7, 138.1, 127.4, 112.6, 112.4, 108.1, 55.8, 51.9, 47.3, 45.5, 29.8, 27.6, 26.3, 22.4, 13.8; **HRMS (APCI)** m/z: $[M+H]^+$ calcd. for $C_{17}H_{24}NO_4$ 306.1700; found 306.1699.

^{1}H and ^{13}C NMR spectra

3aa ^{1}H NMR (400 MHz, $CDCl_3$)

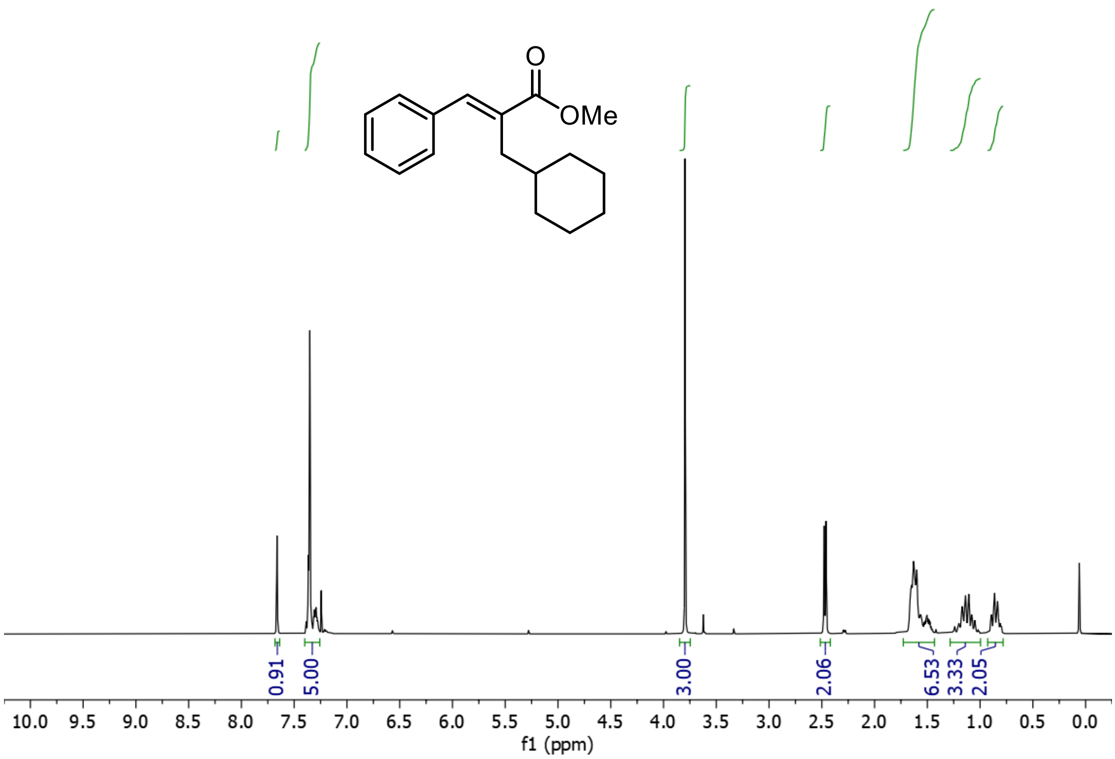

3aa ^{13}C NMR (100 MHz, $CDCl_3$)

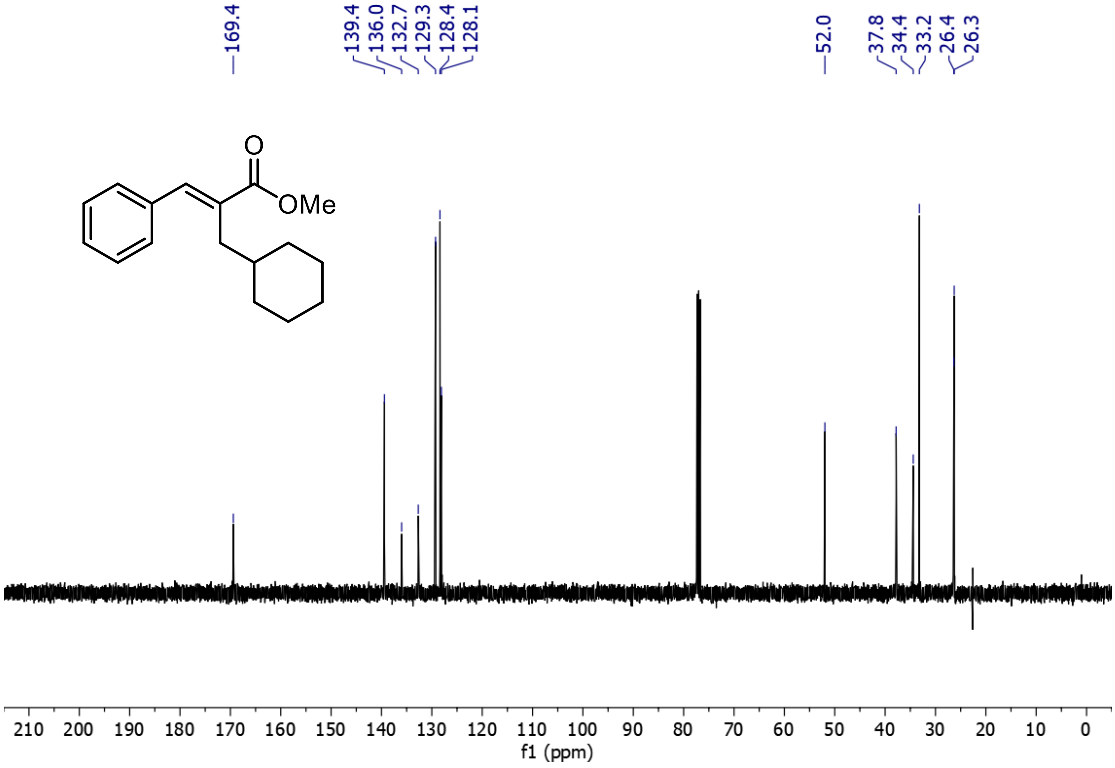

3ba ^{1}H NMR (400 MHz, $CDCl_3$)

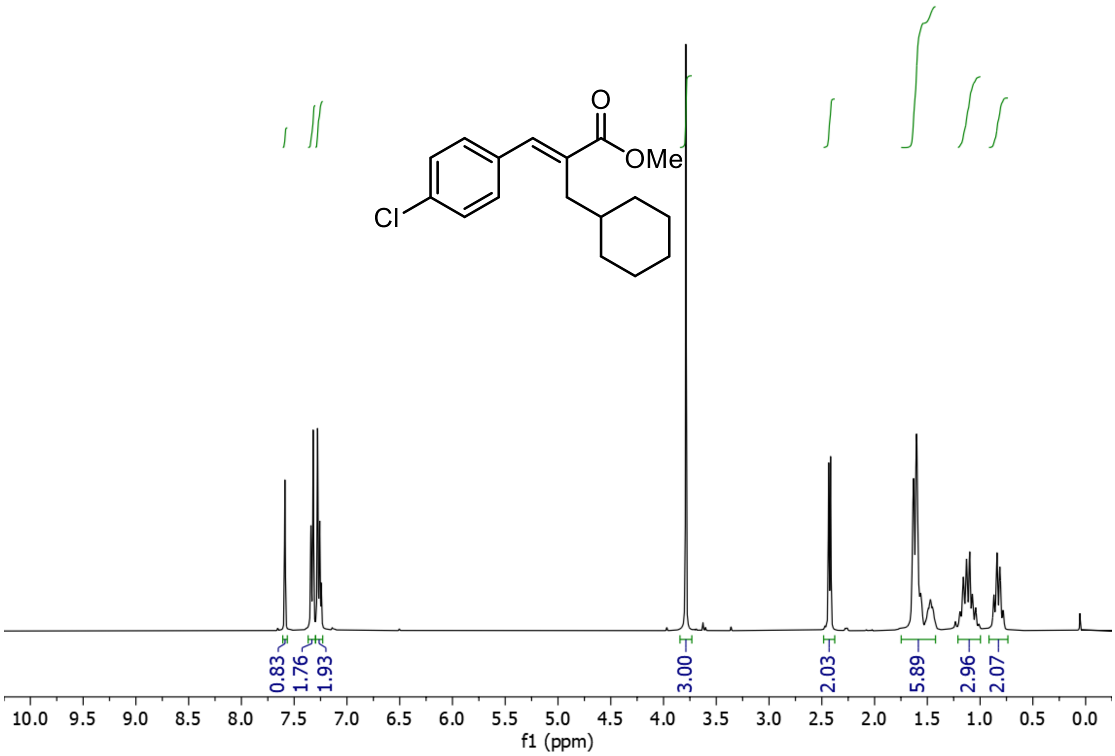

3ba ^{13}C NMR (100 MHz, $CDCl_3$)

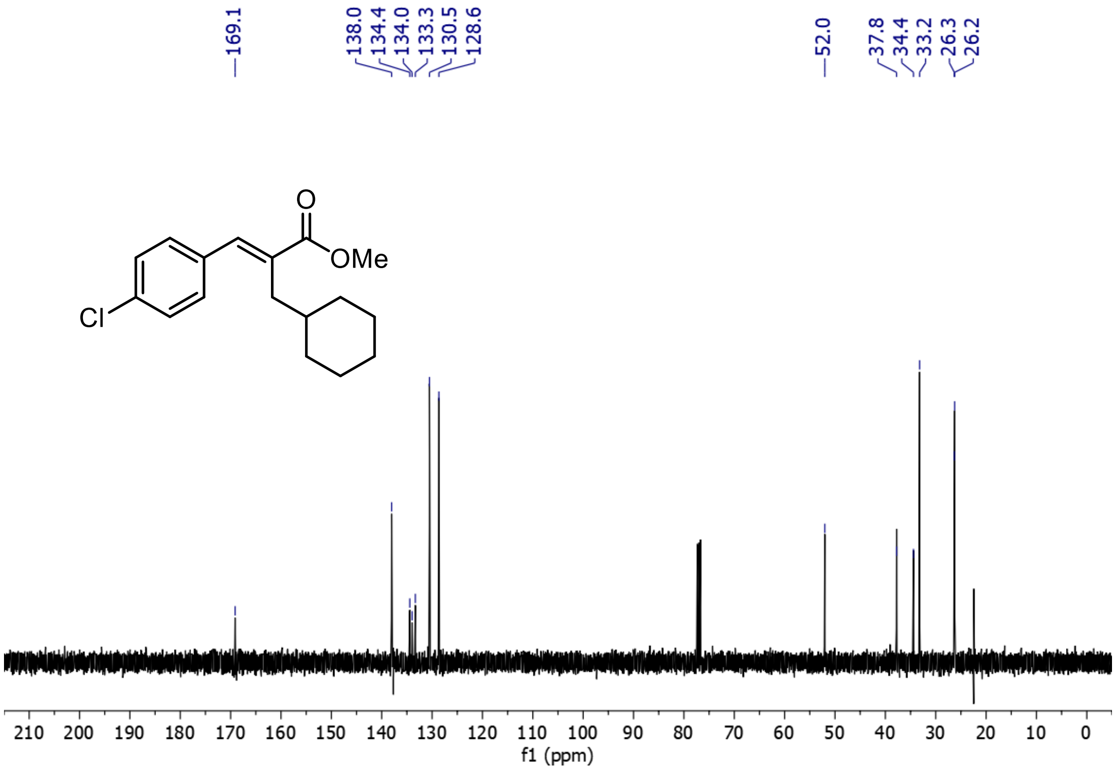

3ca ^{1}H NMR (400 MHz, $CDCl_3$)

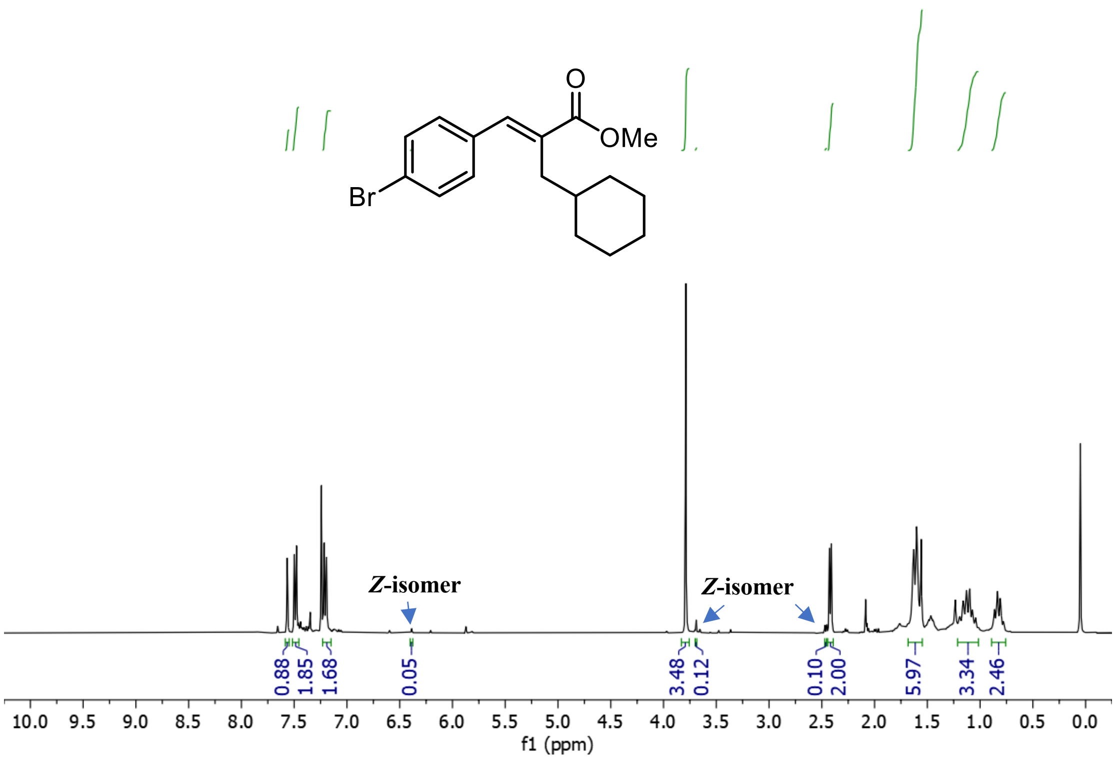

3ca ^{13}C NMR (100 MHz, $CDCl_3$)

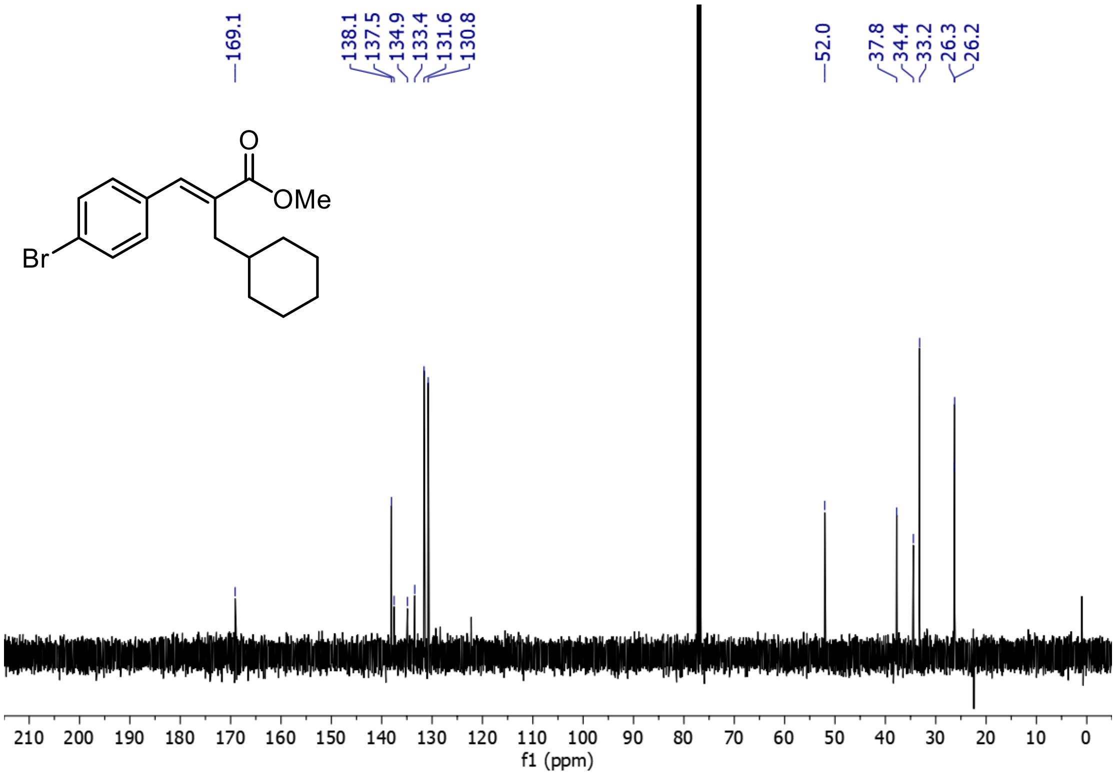

3da ^1H NMR (400 MHz, $CDCl_3$)

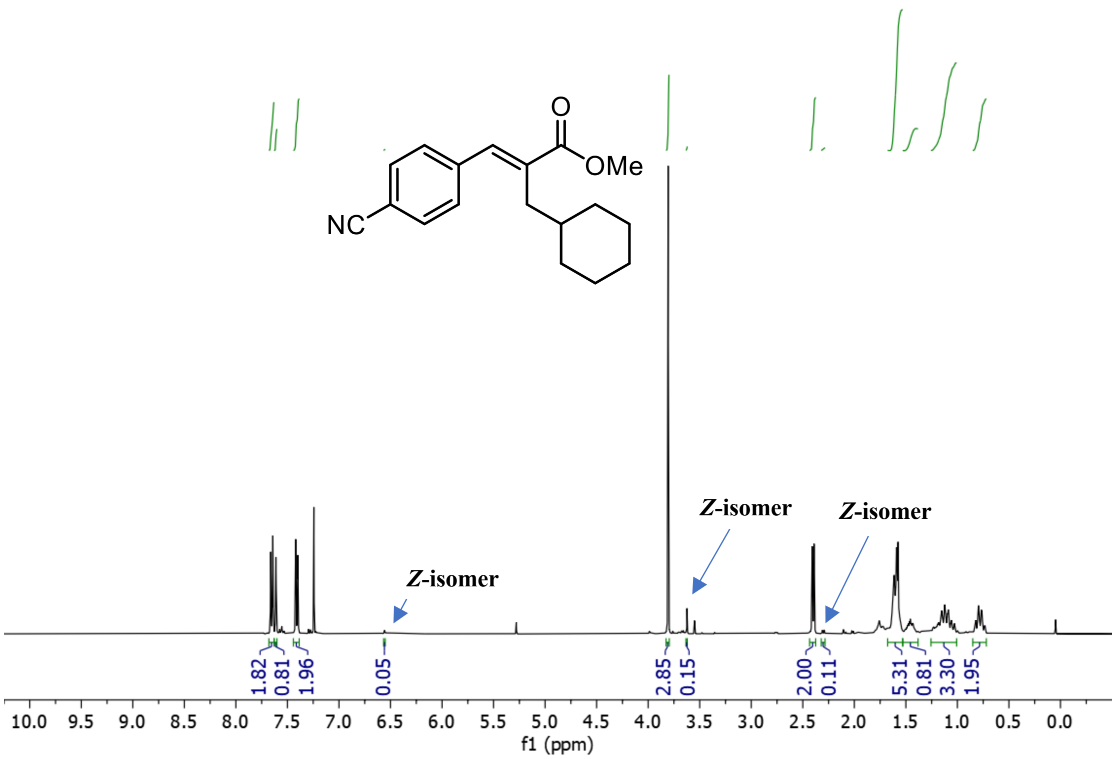

3da ^{13}C NMR (100 MHz, $CDCl_3$)

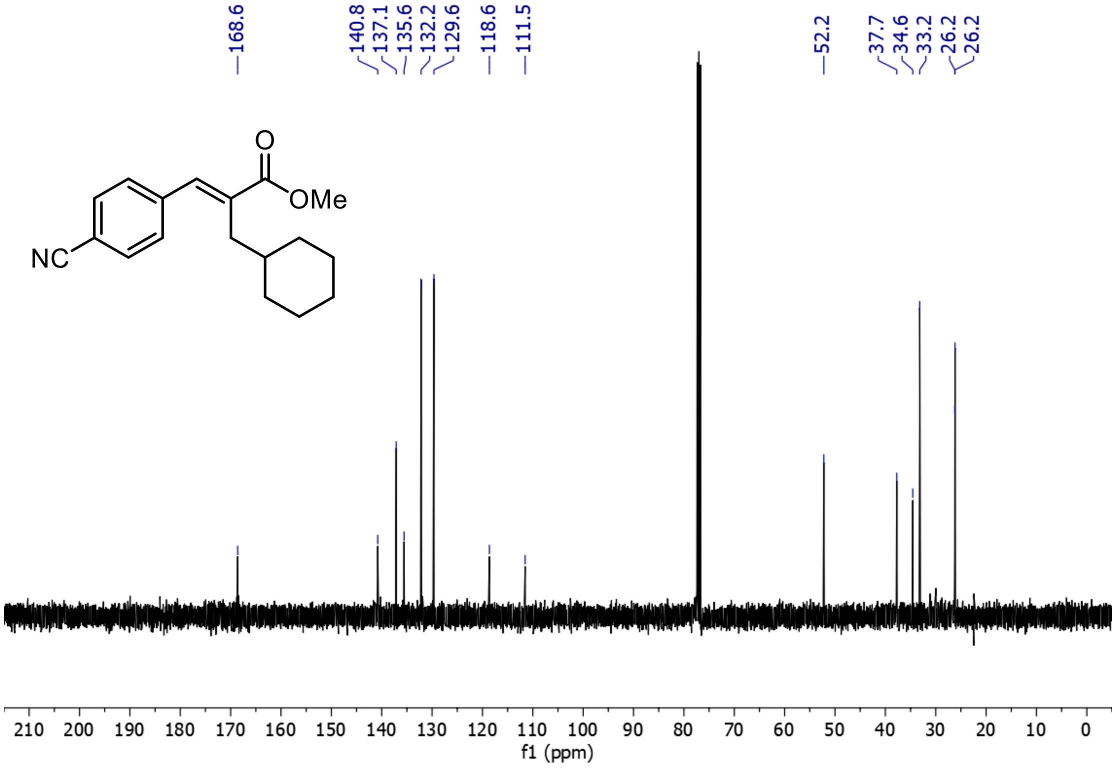

3ea ^{1}H NMR (400 MHz, CDCl$_3$)

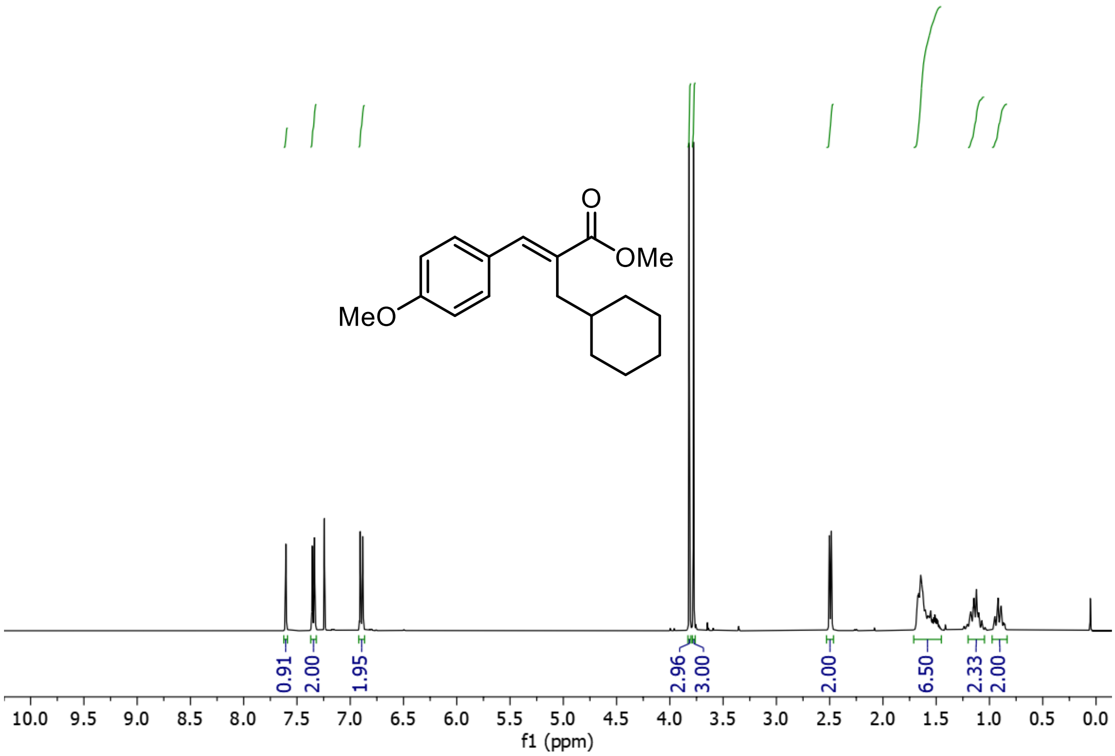

3ea ^{13}C NMR (100 MHz, CDCl$_3$)

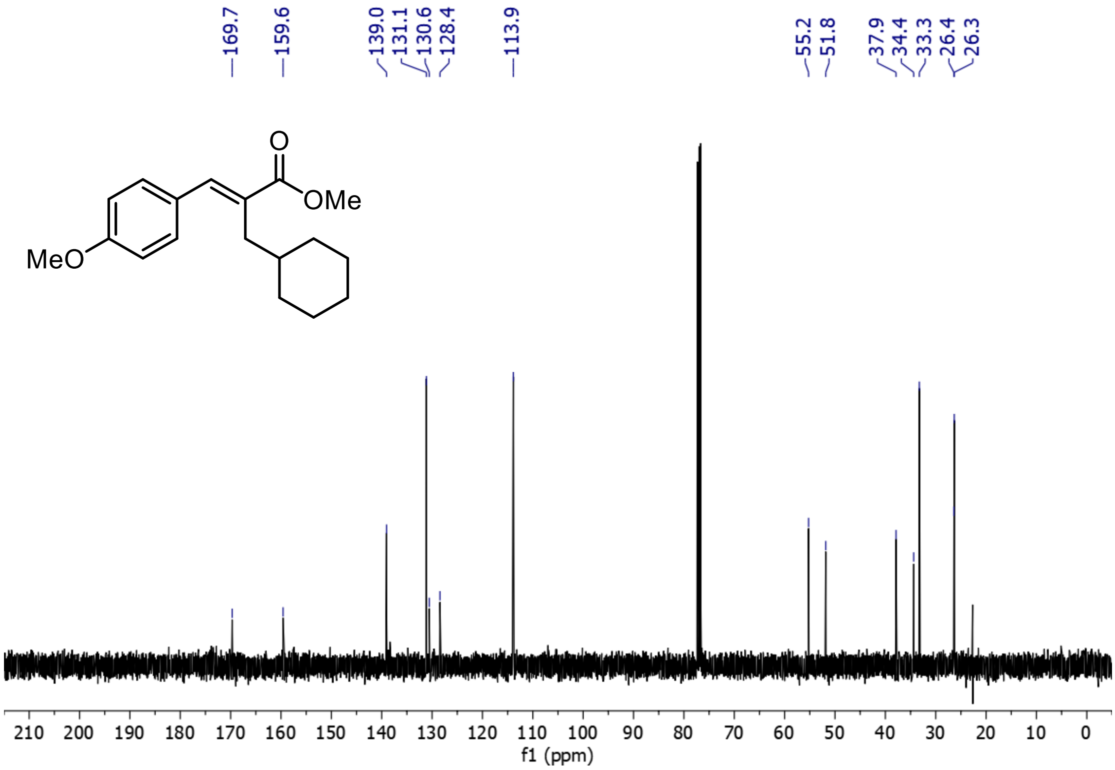

3fa ^{1}H NMR (400 MHz, $CDCl_3$)

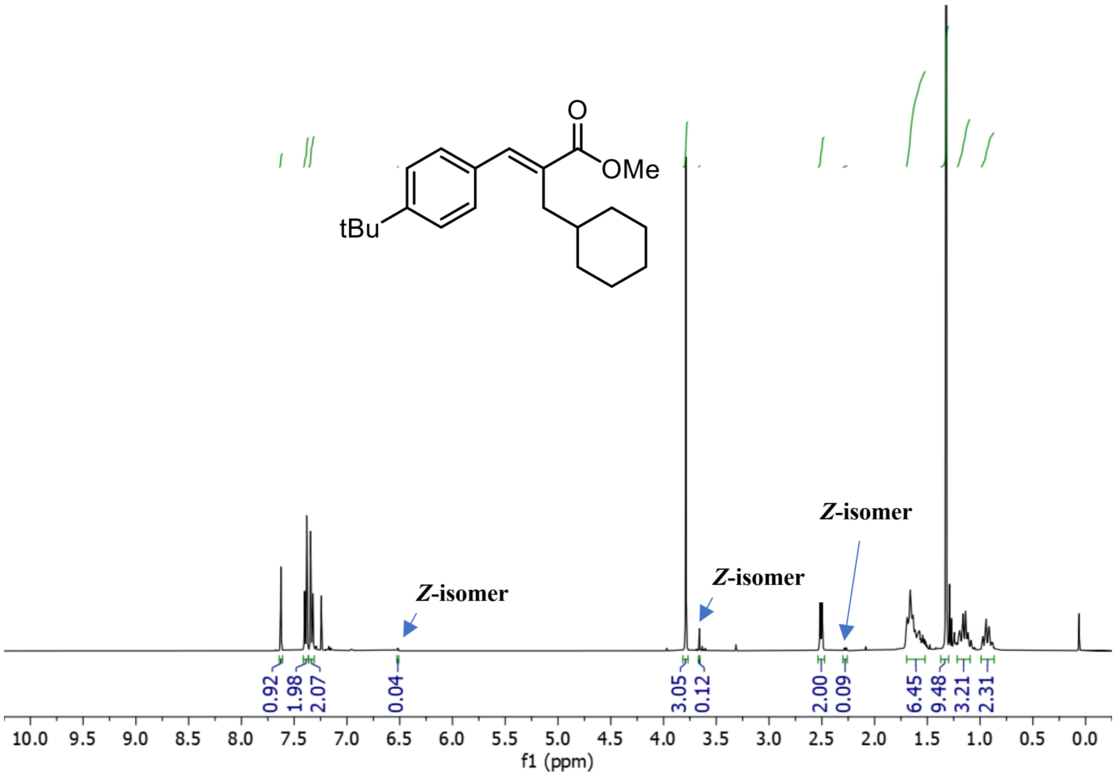

3fa ^{13}C NMR (100 MHz, $CDCl_3$)

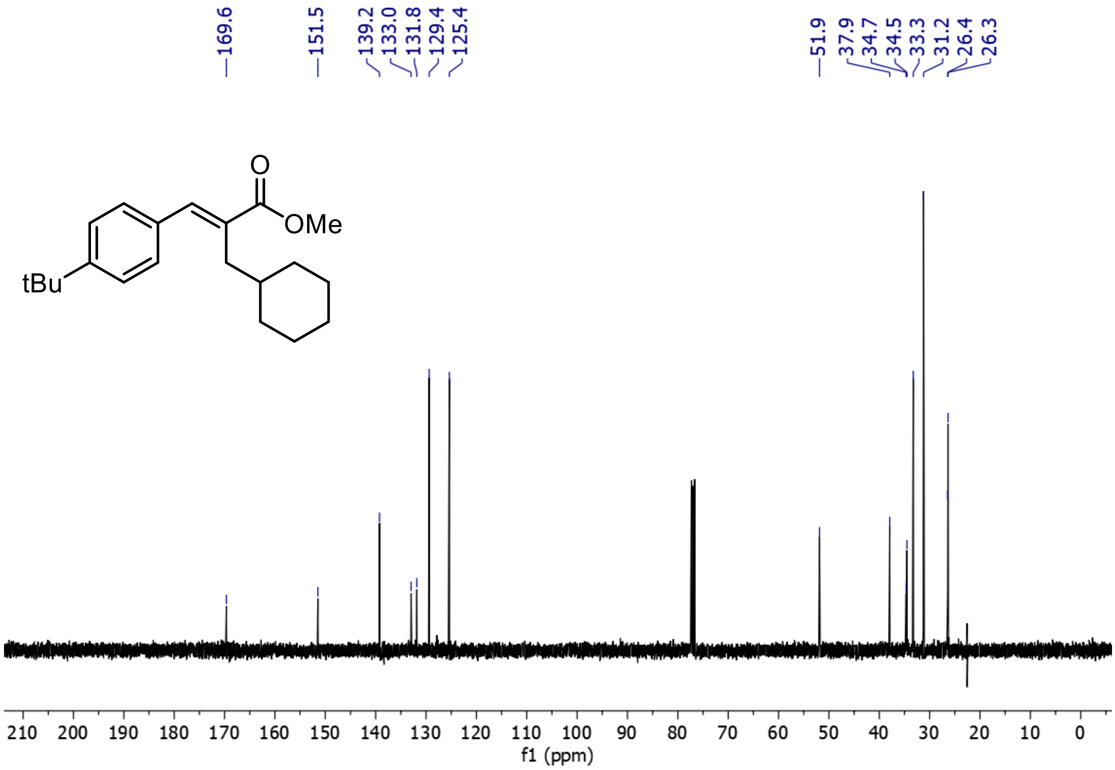

3ga ^{1}H NMR (400 MHz, CDCl$_3$)

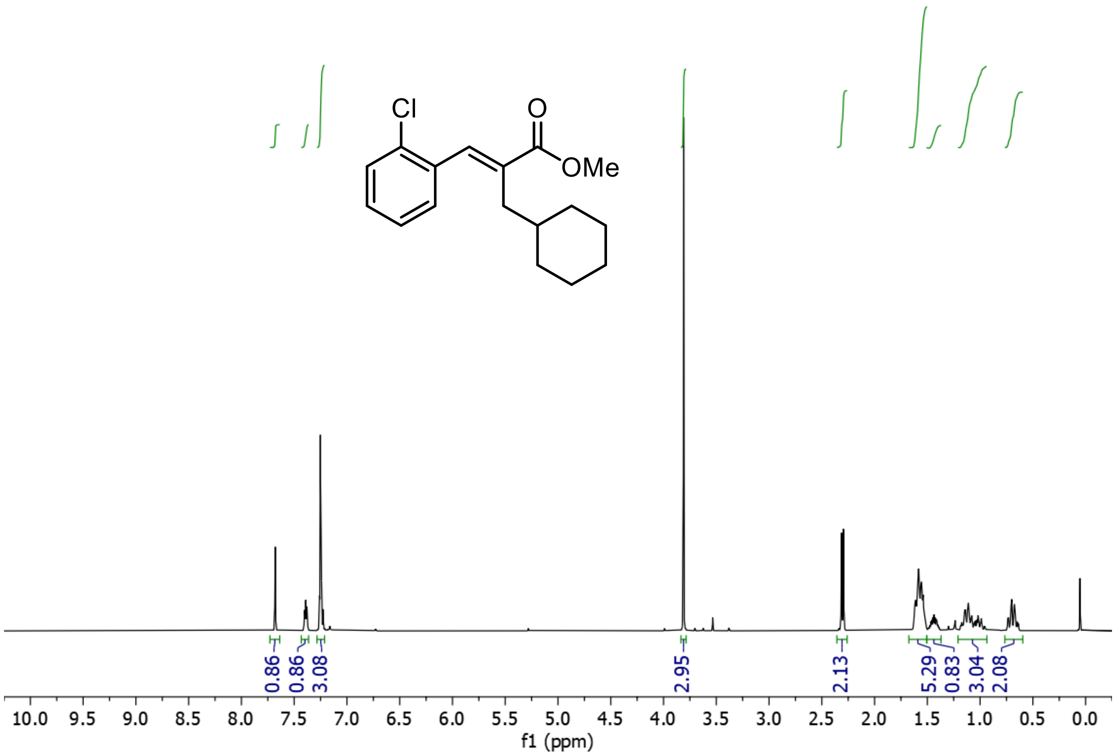

3ga ^{13}C NMR (100 MHz, CDCl$_3$)

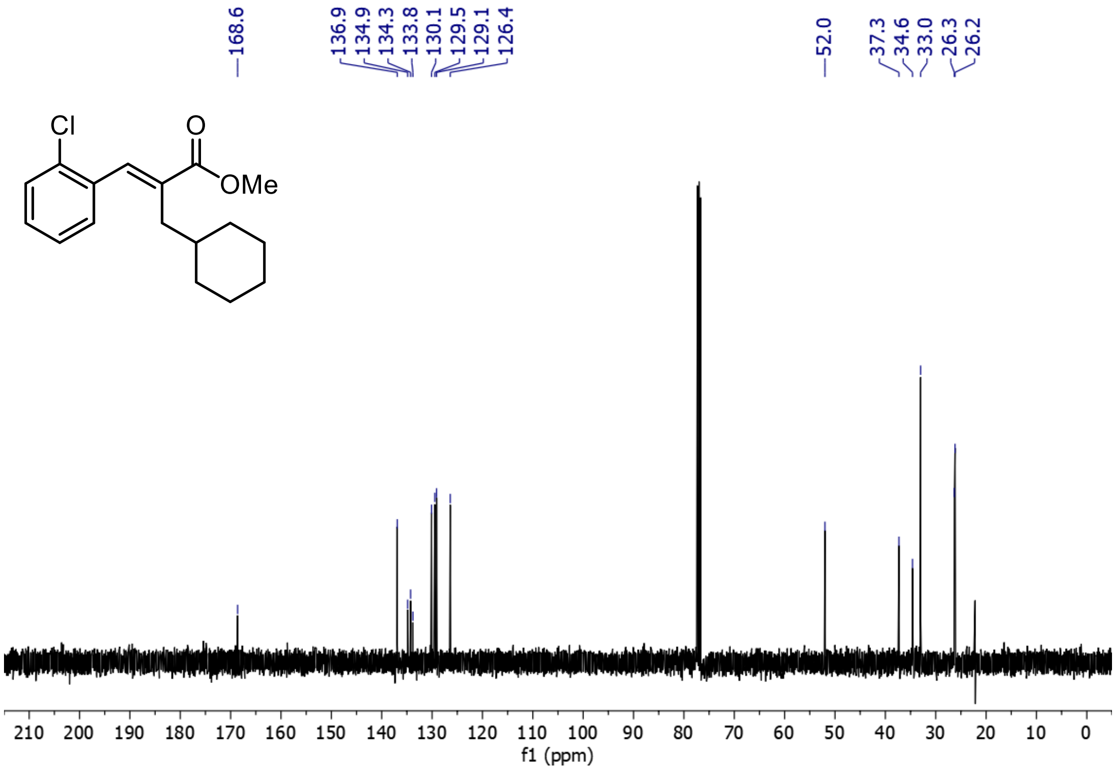

3ha ^{1}H NMR (400 MHz, $CDCl_3$)

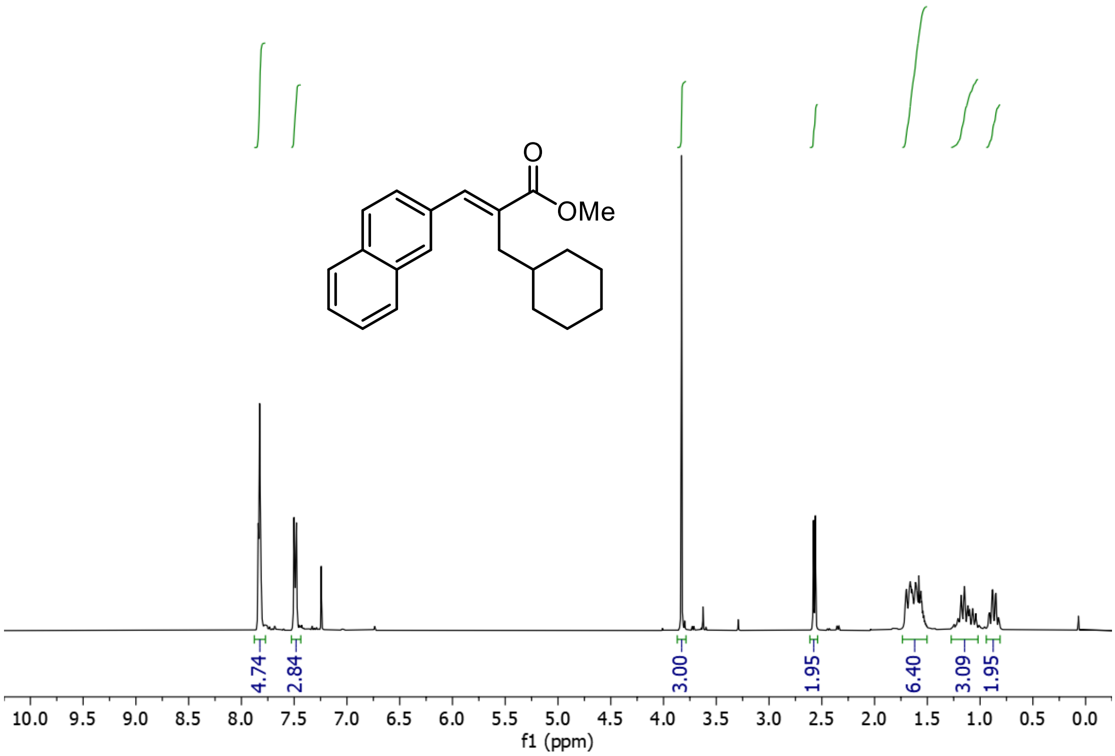

3ha ^{13}C NMR (100 MHz, $CDCl_3$)

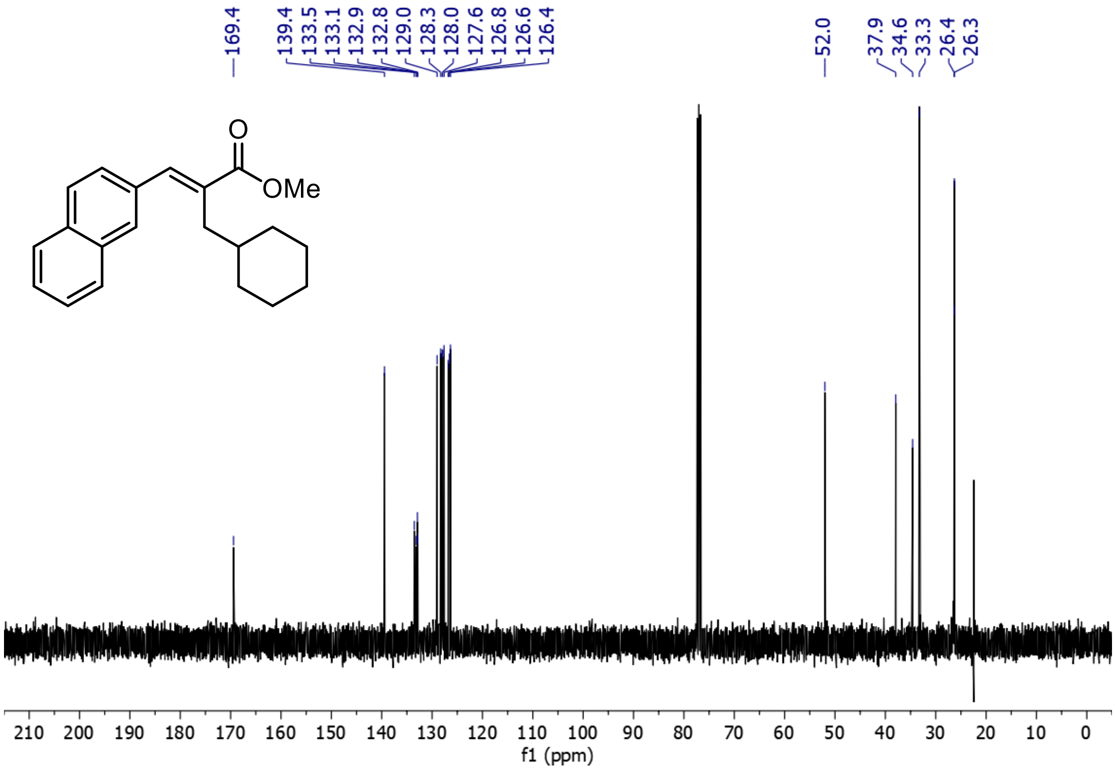

3ia ^{1}H NMR (400 MHz, CDCl$_3$)

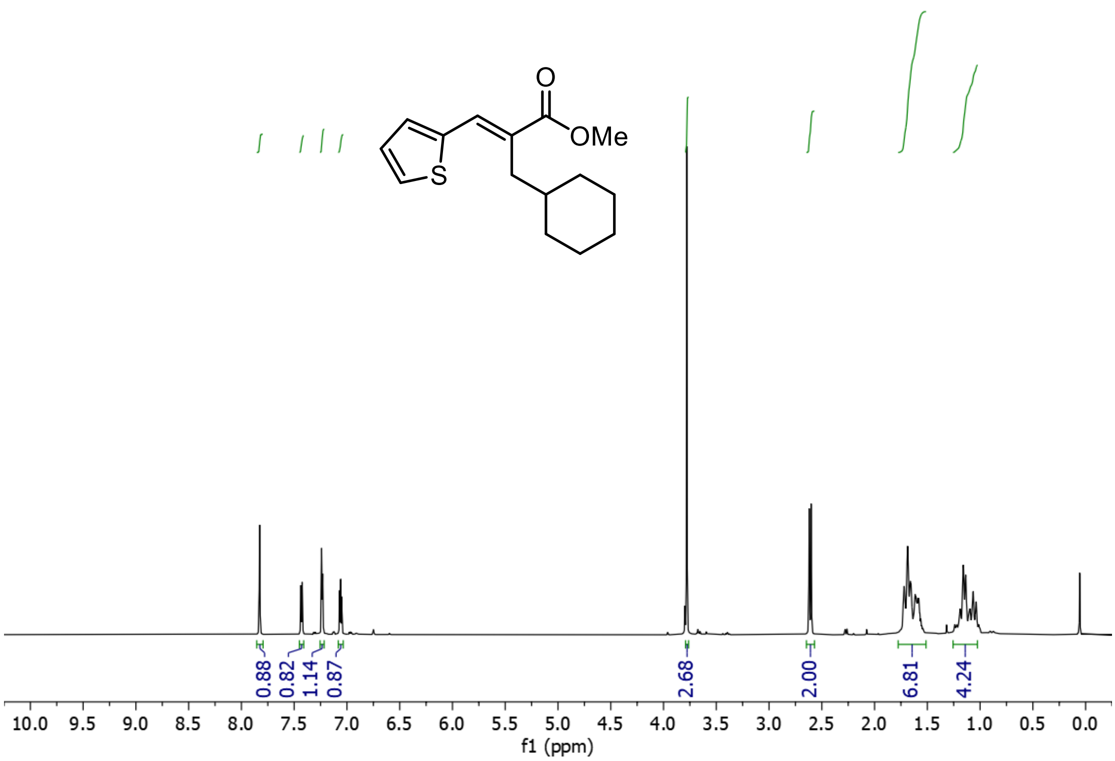

3ia ^{13}C NMR (100 MHz, CDCl$_3$)

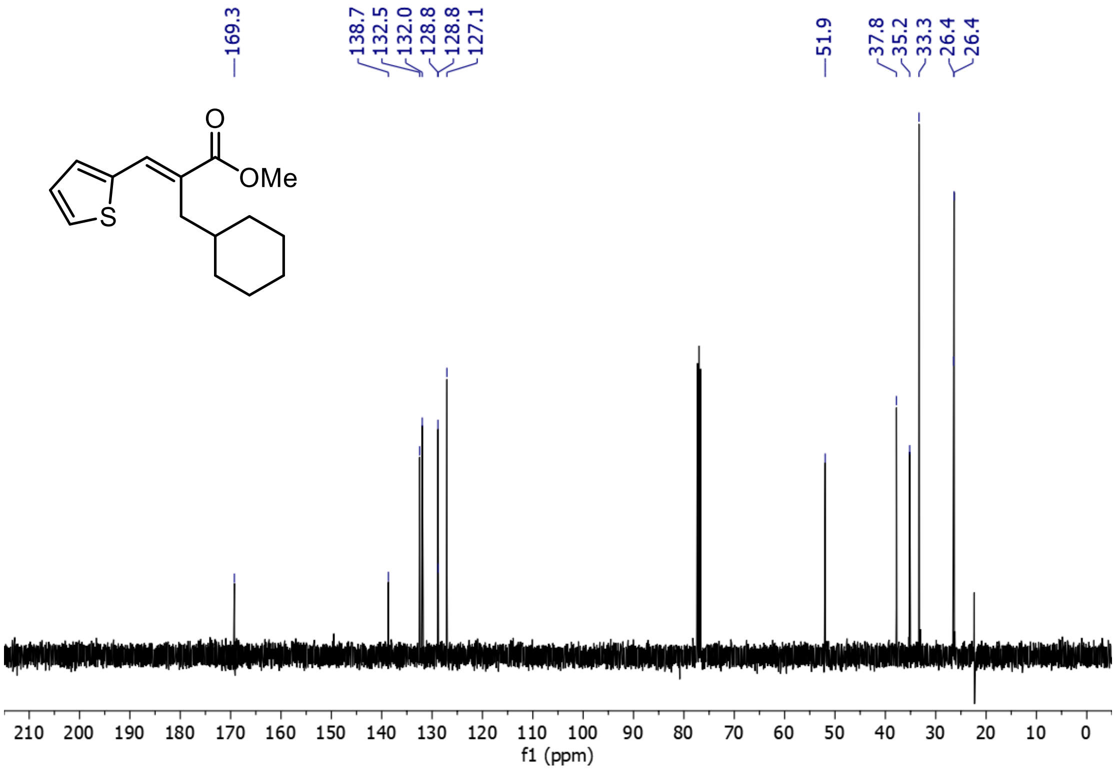

3ja' ^{1}H NMR (400 MHz, $CDCl_3$)

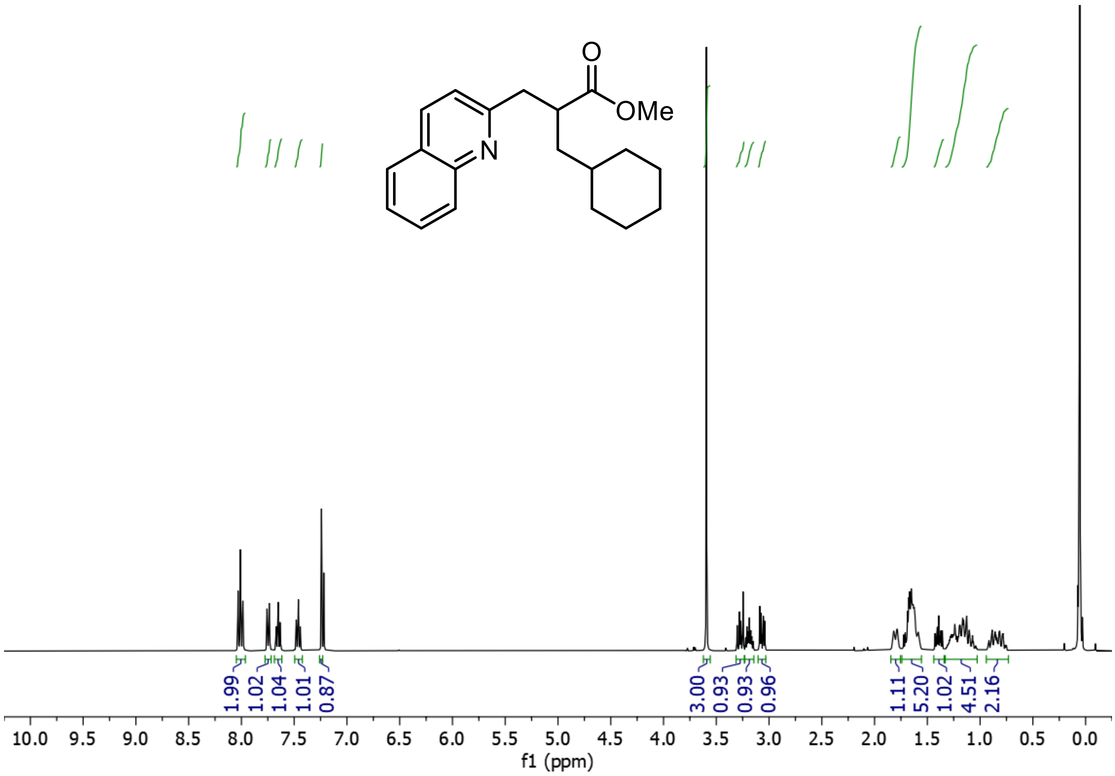

3ja' ^{13}C NMR (100 MHz, $CDCl_3$)

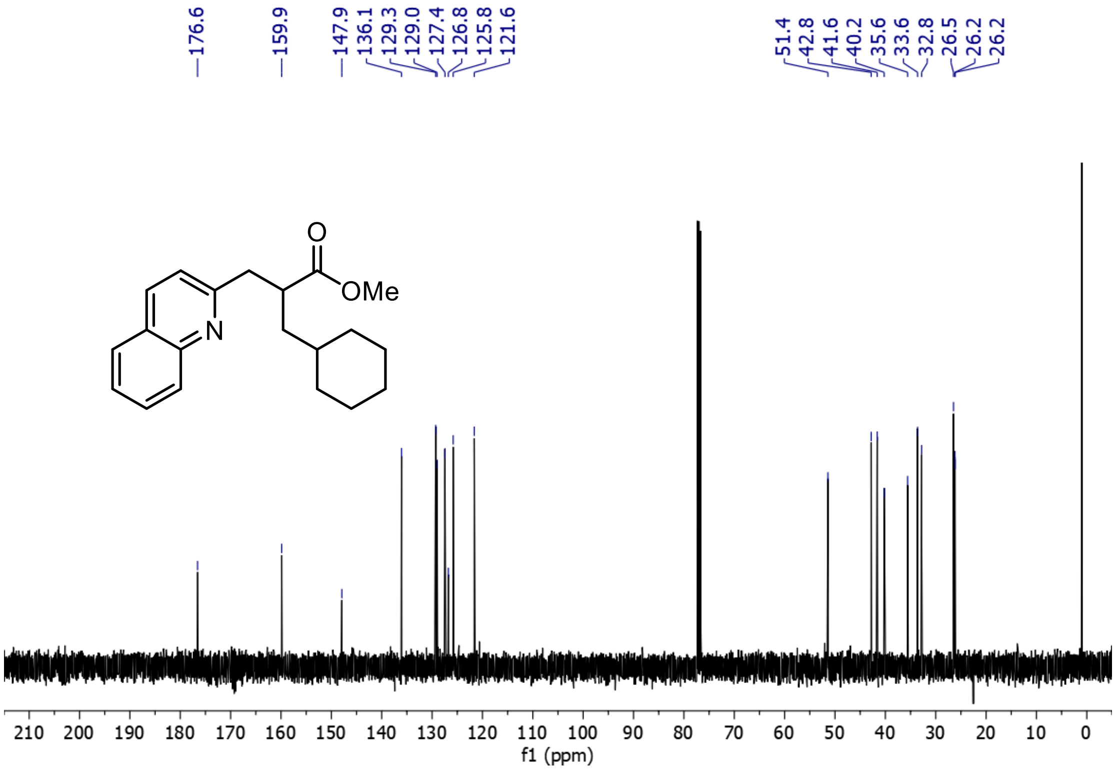

3ka ^{1}H NMR (400 MHz, $CDCl_3$)

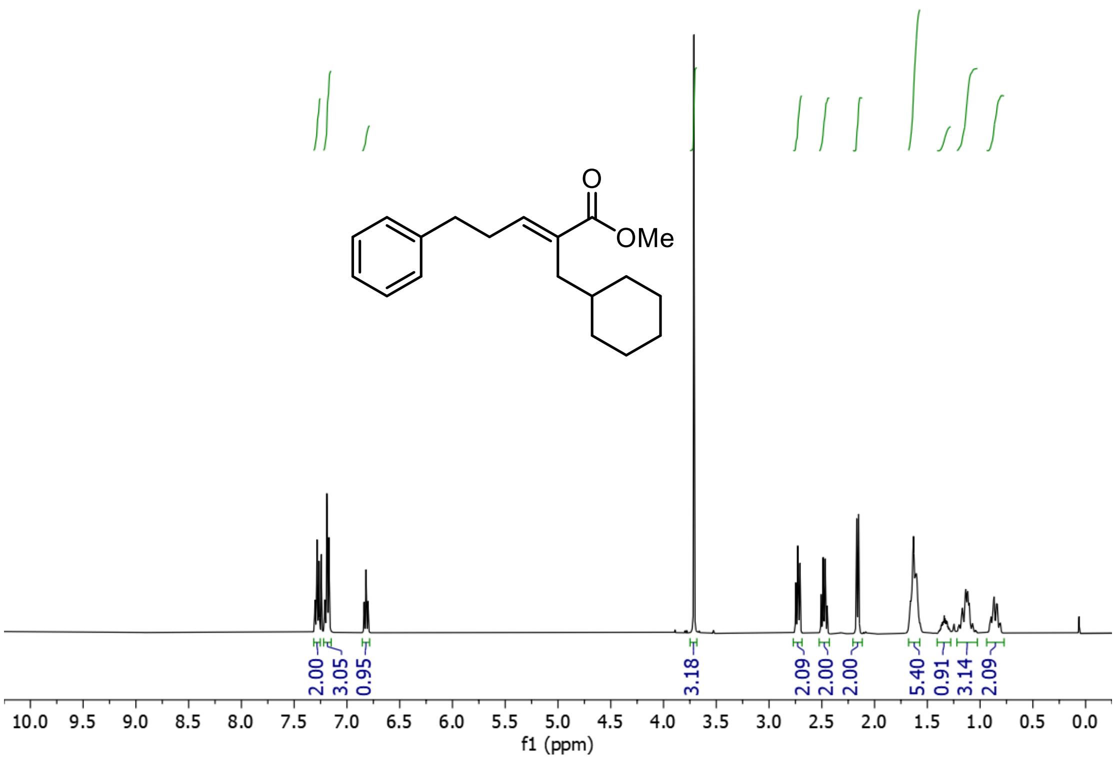

3ka ^{13}C NMR (100 MHz, $CDCl_3$)

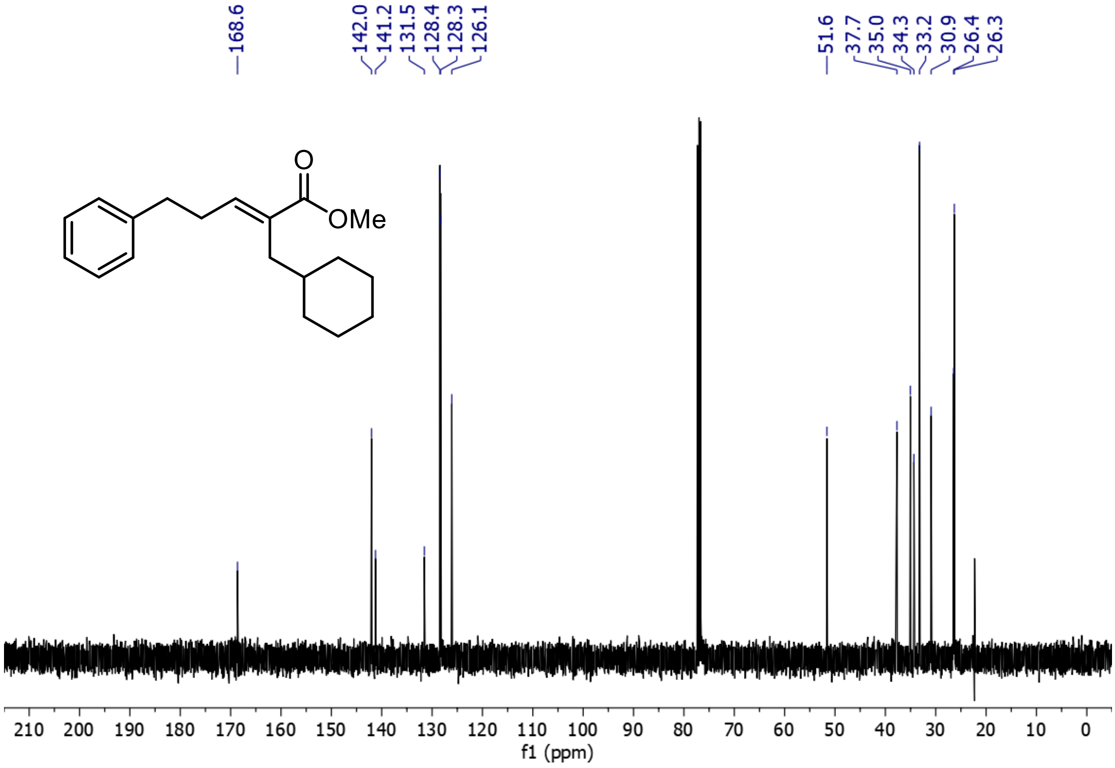

3la ^{1}H NMR (400 MHz, $CDCl_3$)

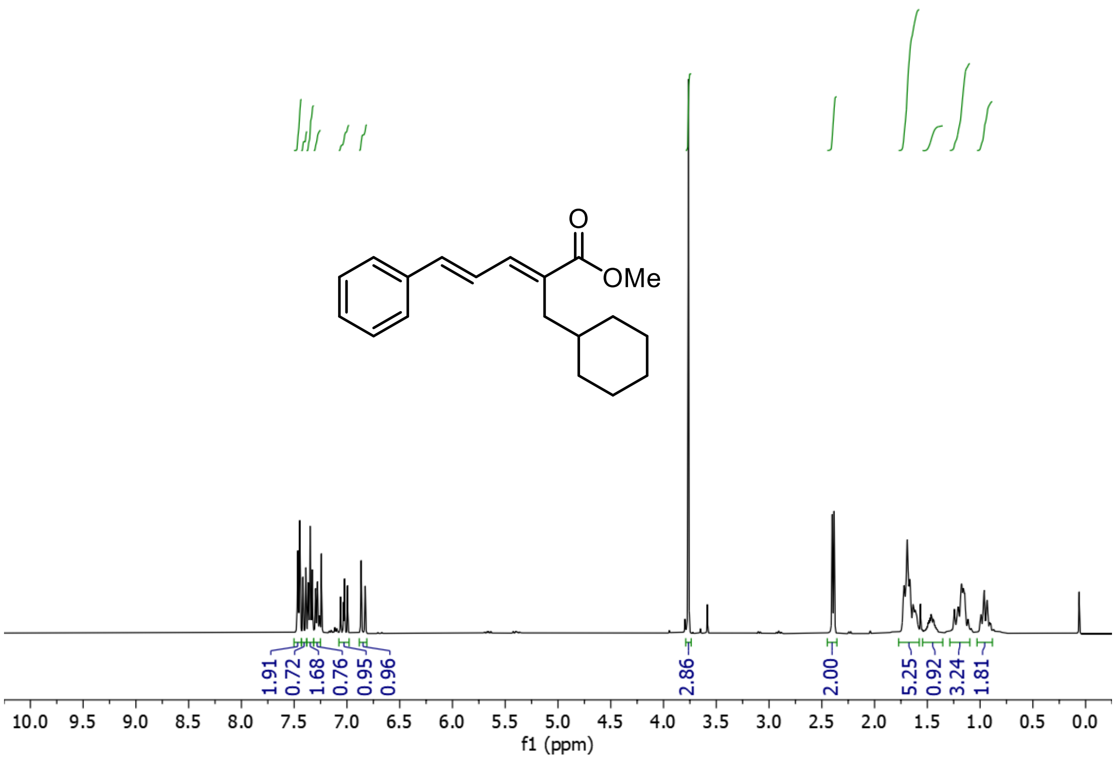

3la ^{13}C NMR (100 MHz, $CDCl_3$)

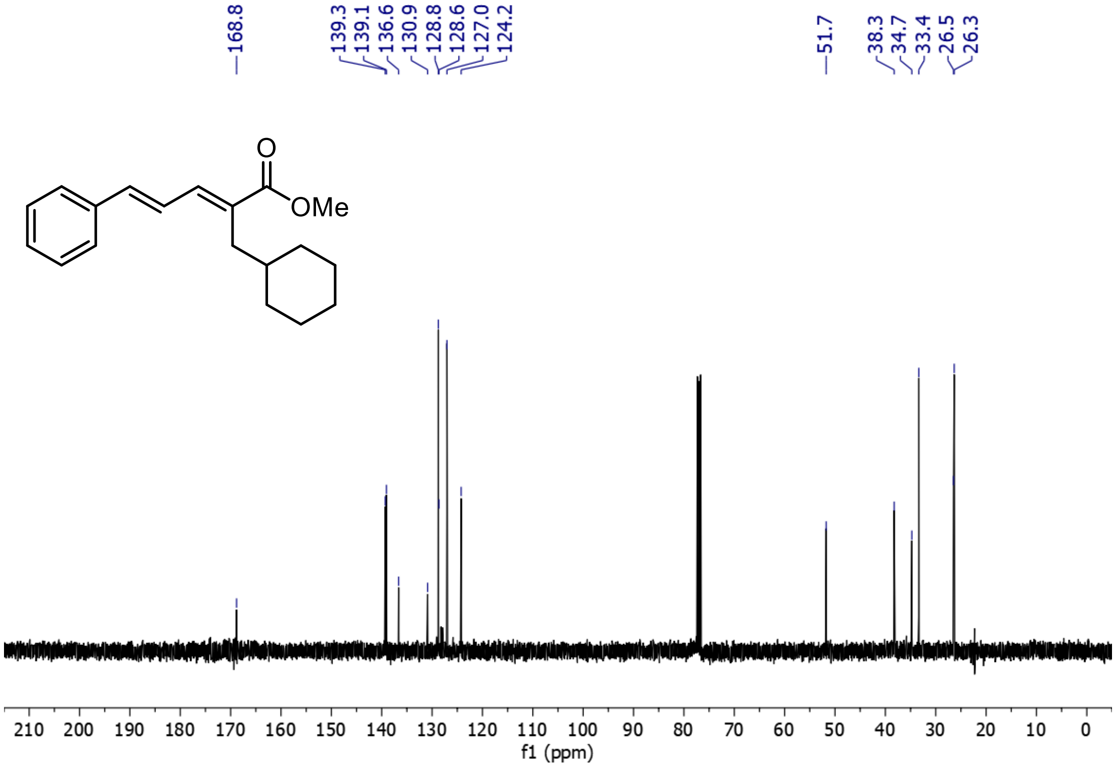

3ma ^1H NMR (400 MHz, $CDCl_3$)

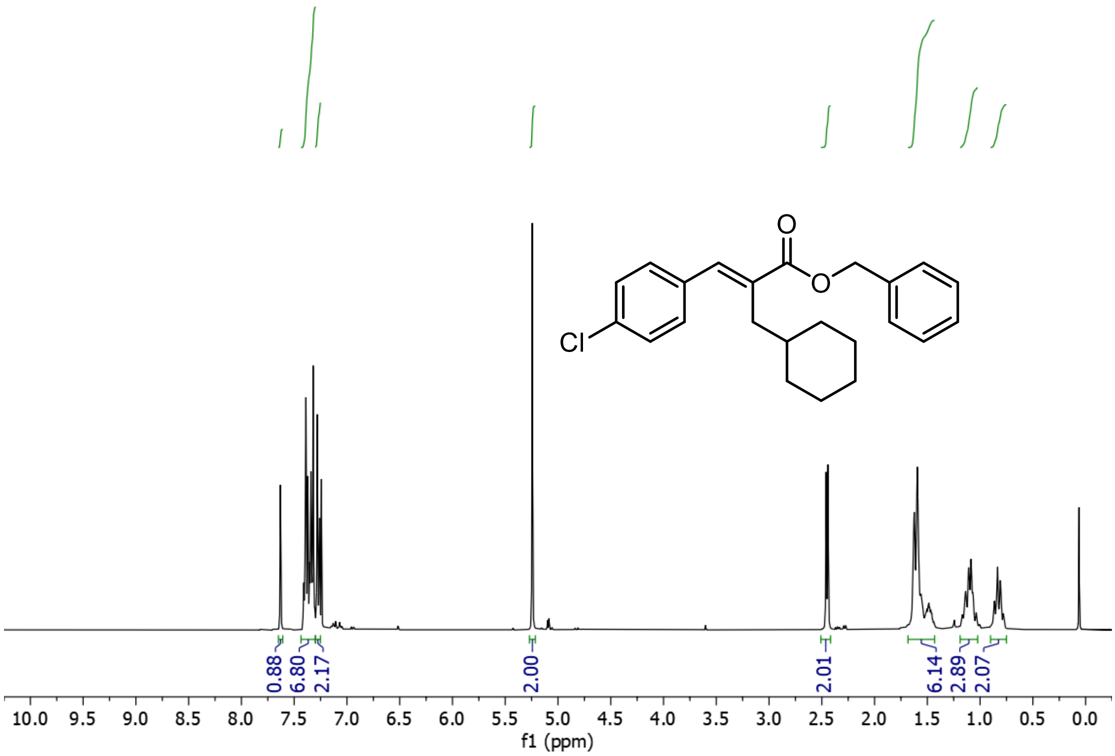

3ma ^{13}C NMR (100 MHz, $CDCl_3$)

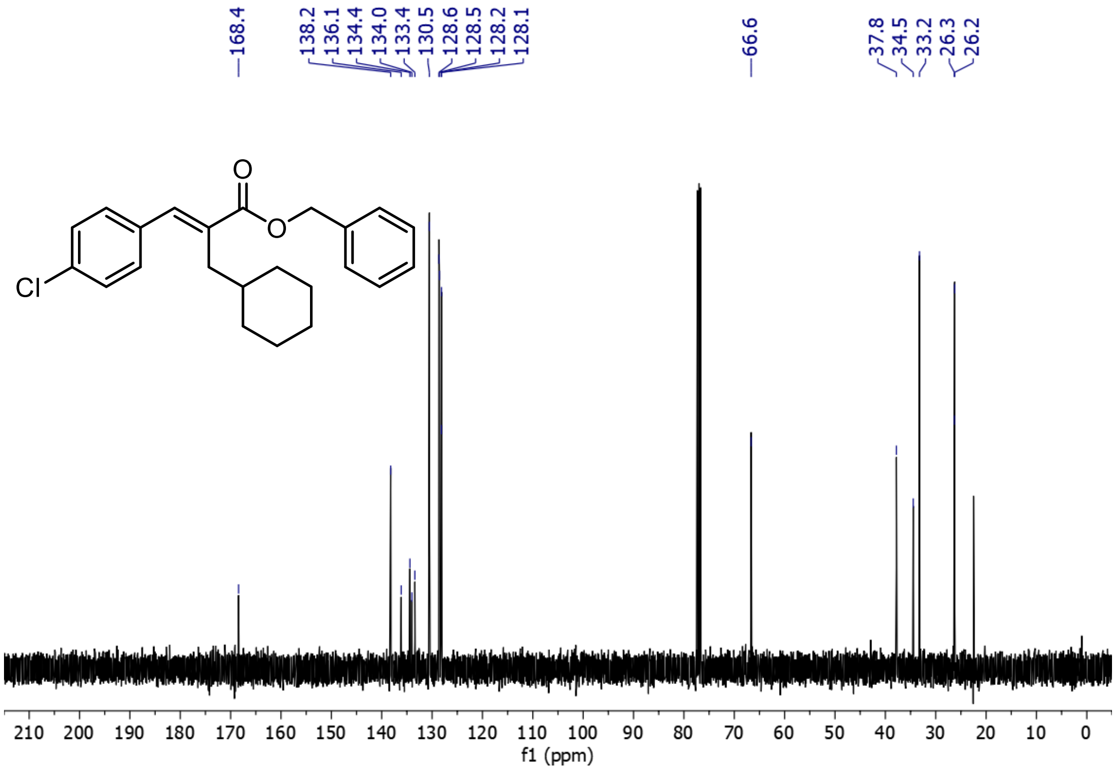

3na ^1H NMR (400 MHz, $CDCl_3$)

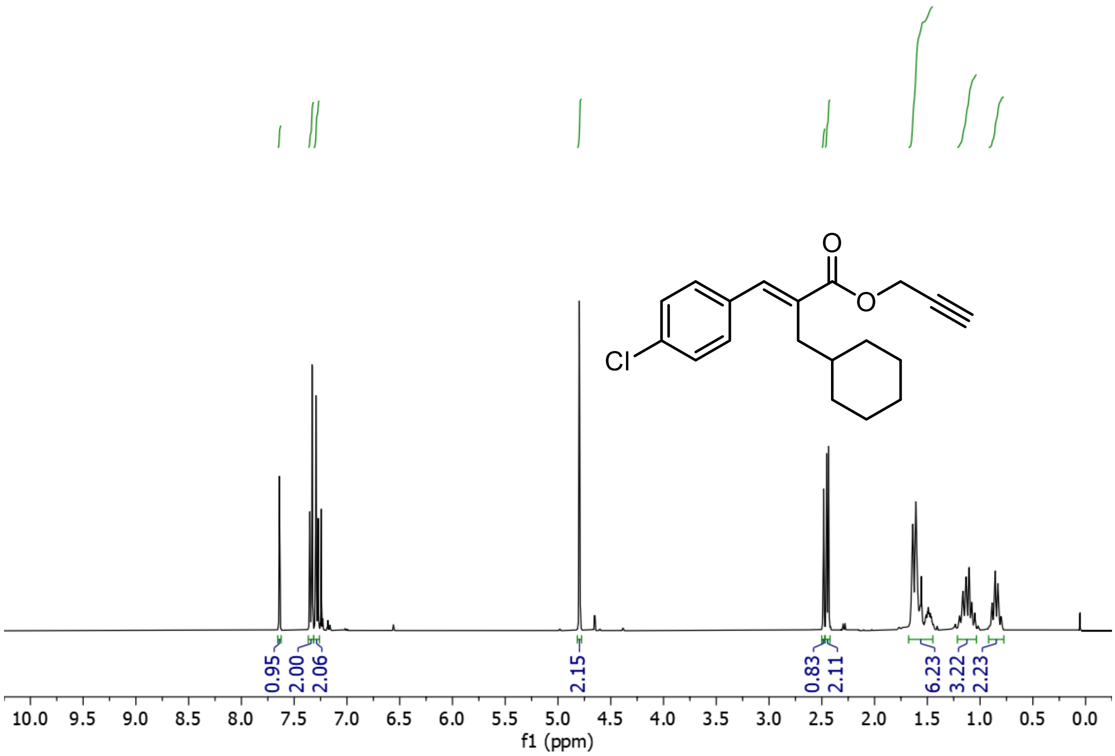

3na ^{13}C NMR (100 MHz, $CDCl_3$)

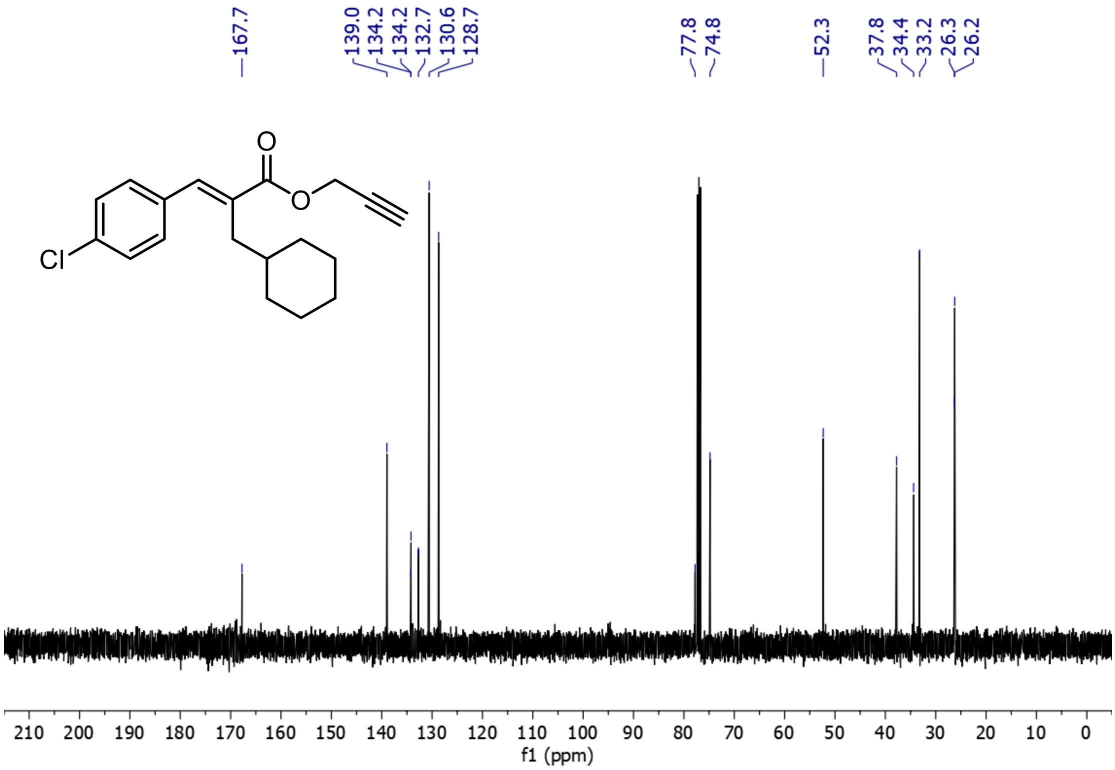

3oa ^{1}H NMR (400 MHz, $CDCl_3$)

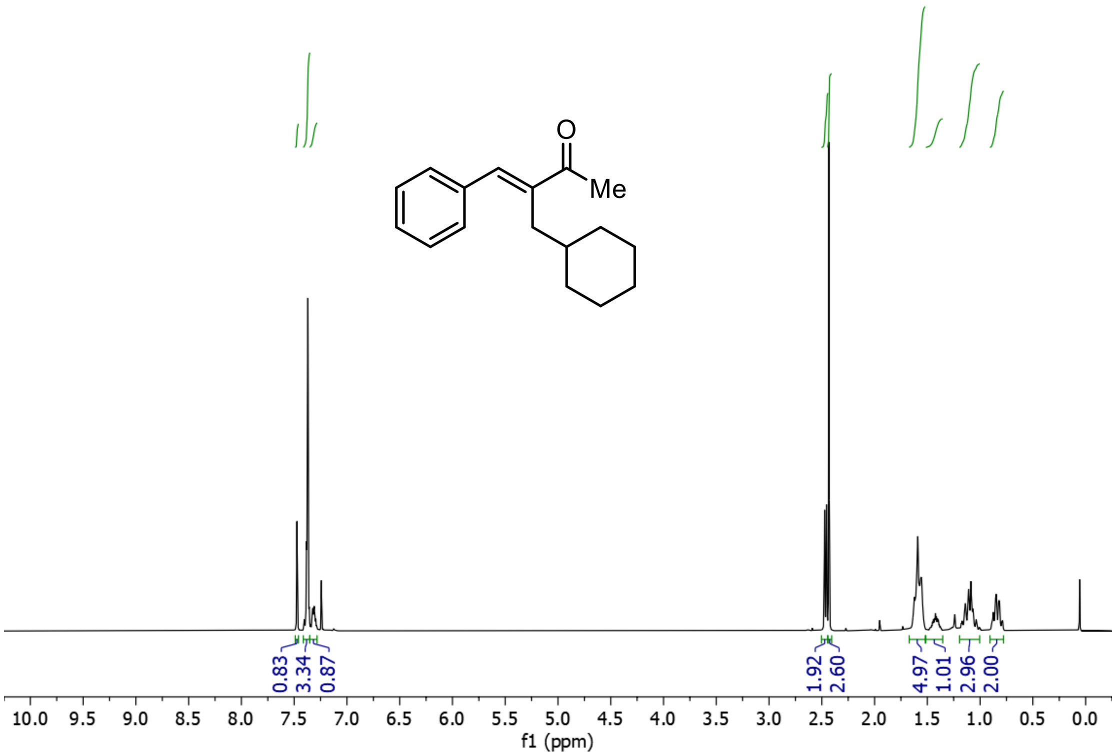

3oa ^{13}C NMR (100 MHz, $CDCl_3$)

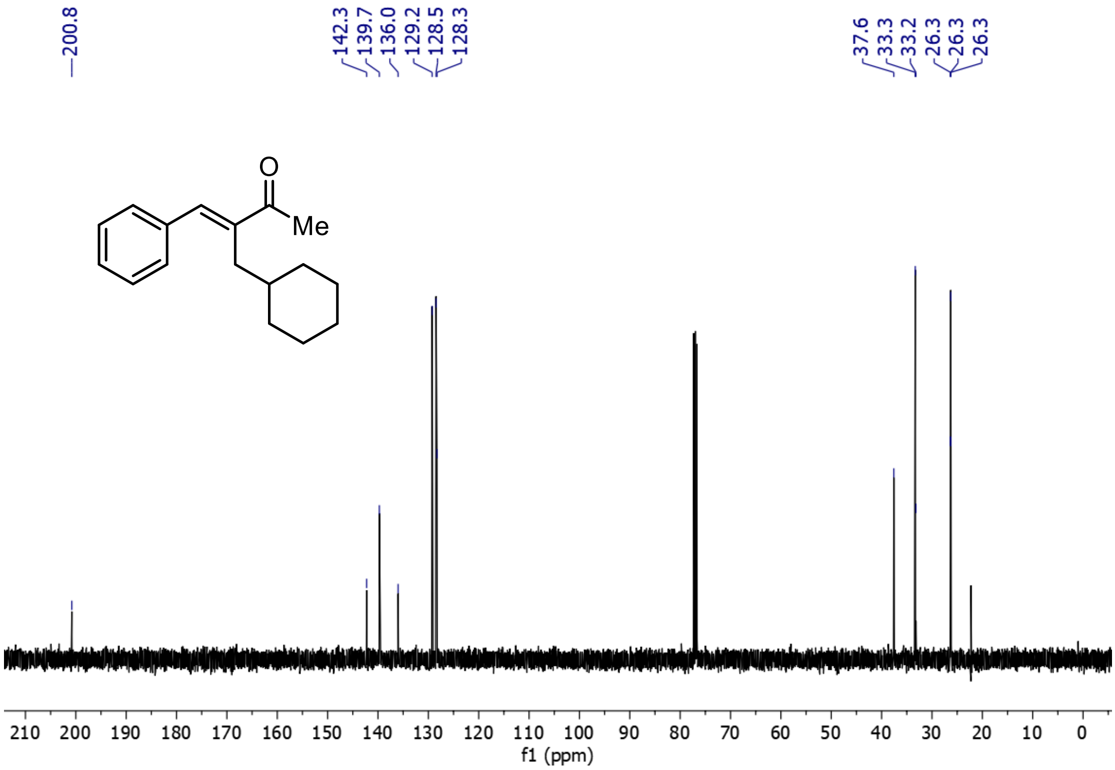

3ab ^1H NMR (400 MHz, $CDCl_3$)

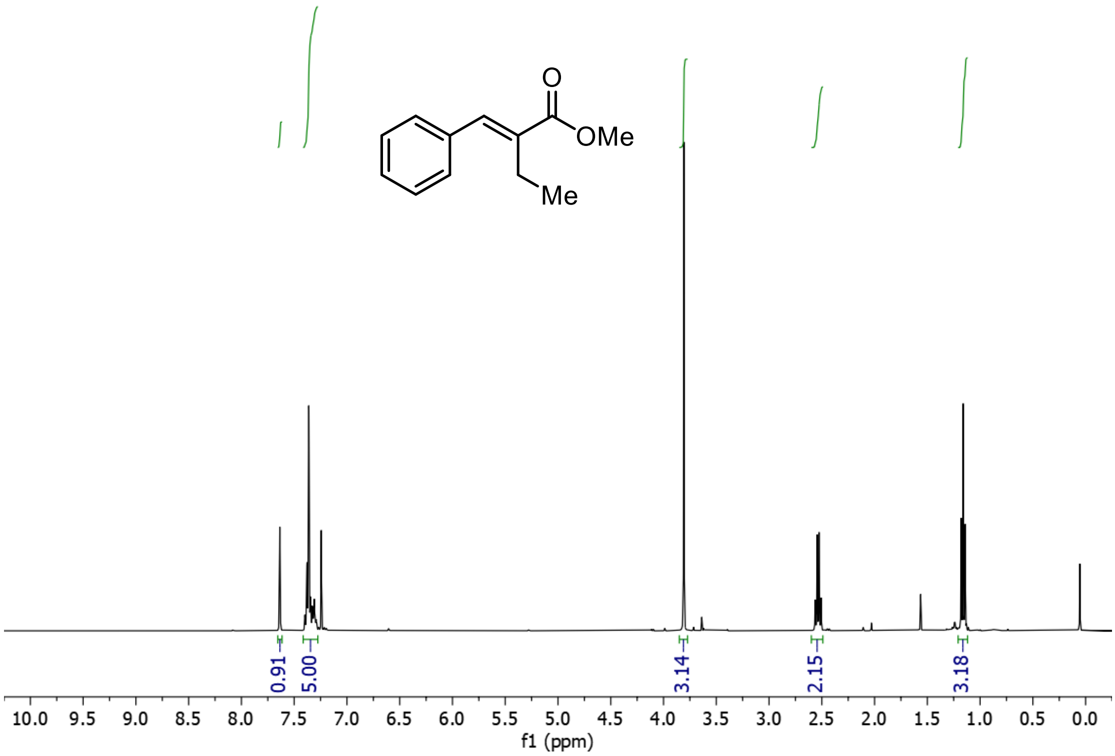

3ab ^1H NMR (100 MHz, $CDCl_3$)

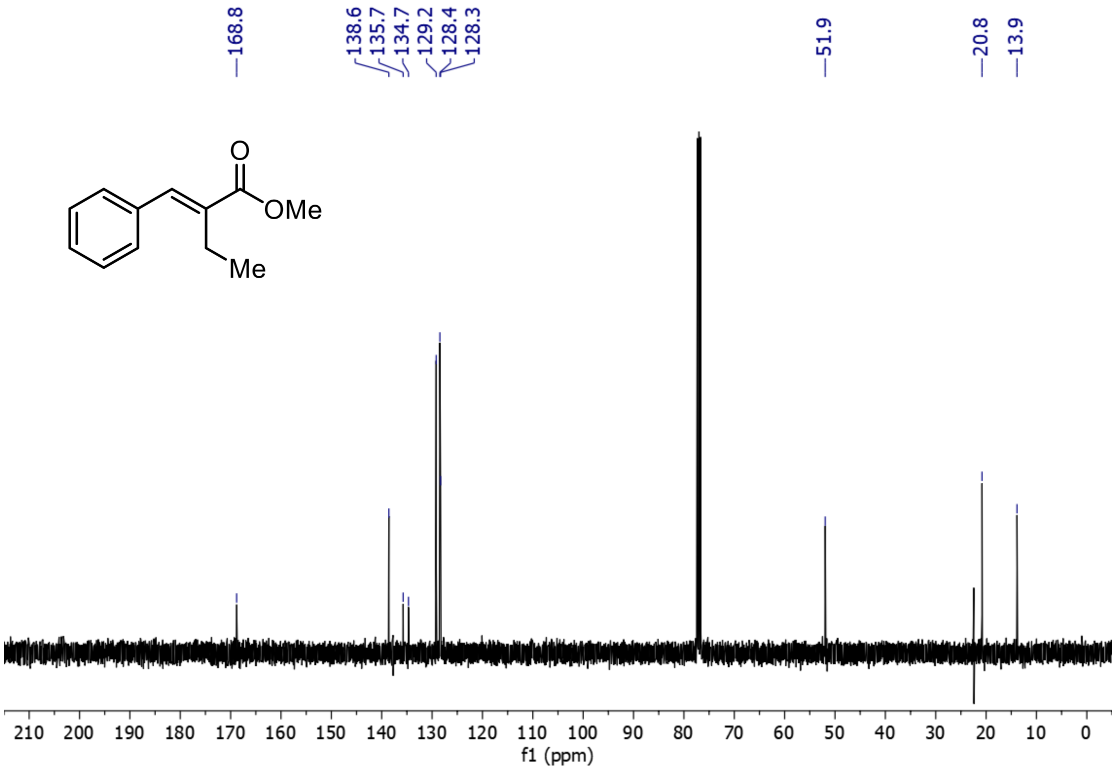

3ac ^{1}H NMR (400 MHz, $CDCl_3$)

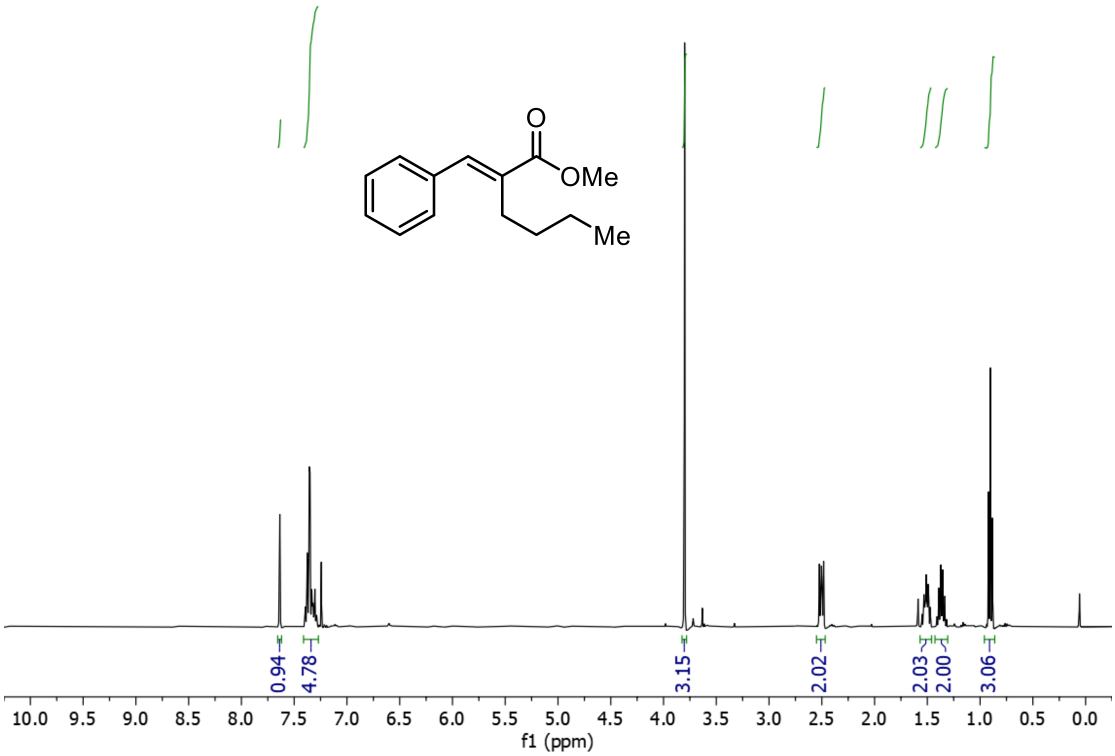

3ac ^{13}C NMR (100 MHz, $CDCl_3$)

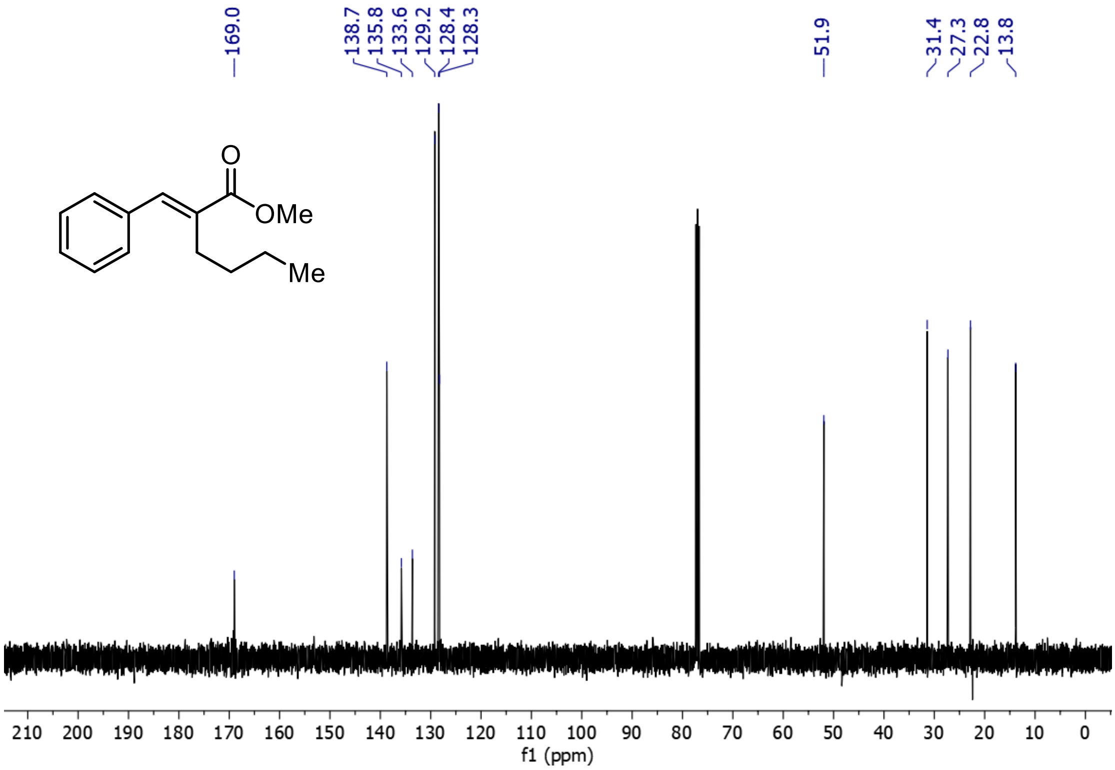

3ad ^{1}H NMR (400 MHz, $CDCl_3$)

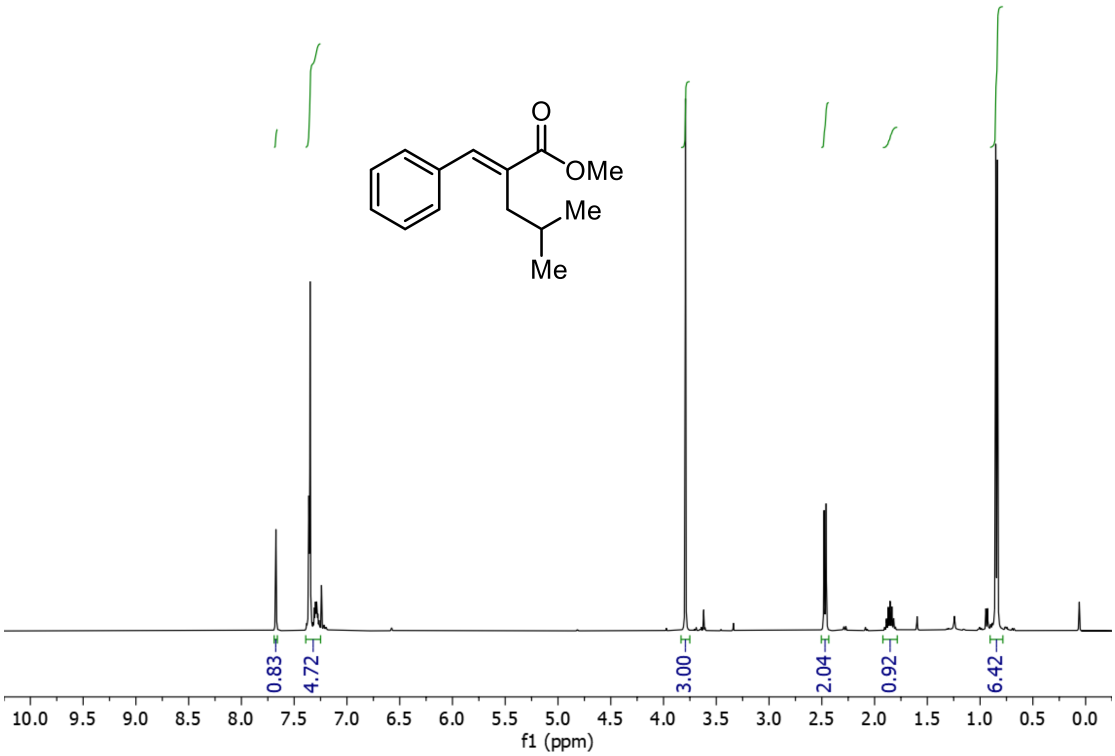

3ad ^{13}C NMR (100 MHz, $CDCl_3$)

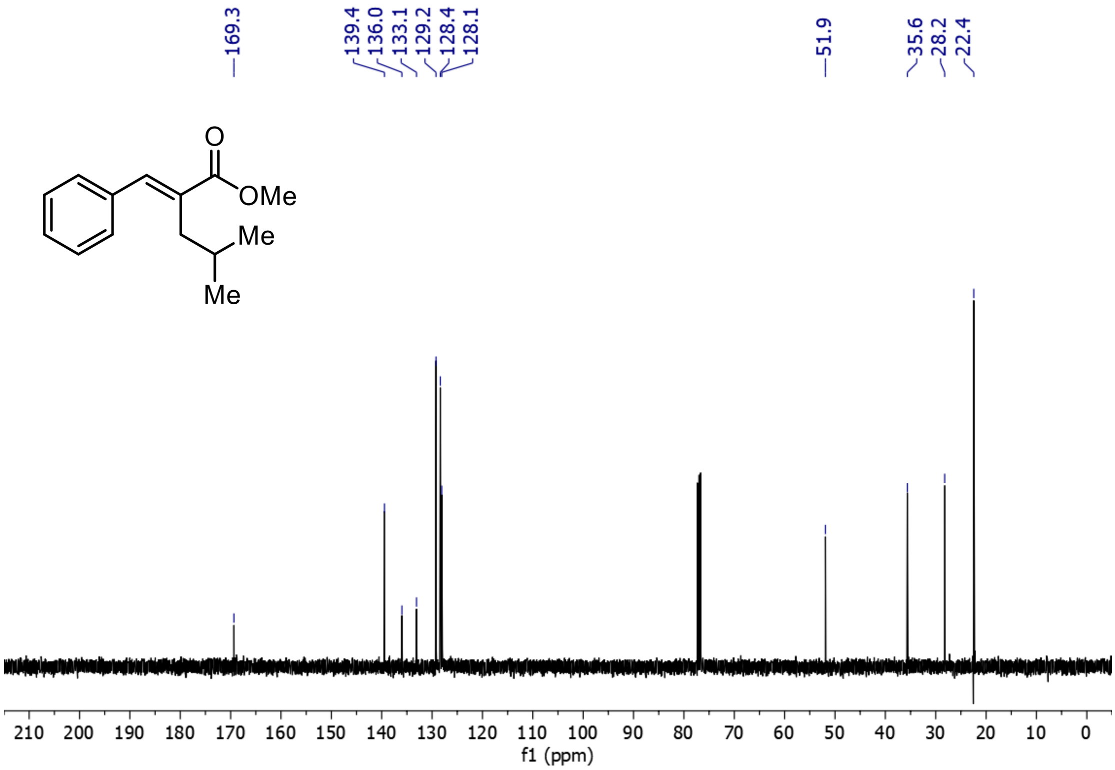

3ae ^{1}H NMR (400 MHz, CDCl$_3$)

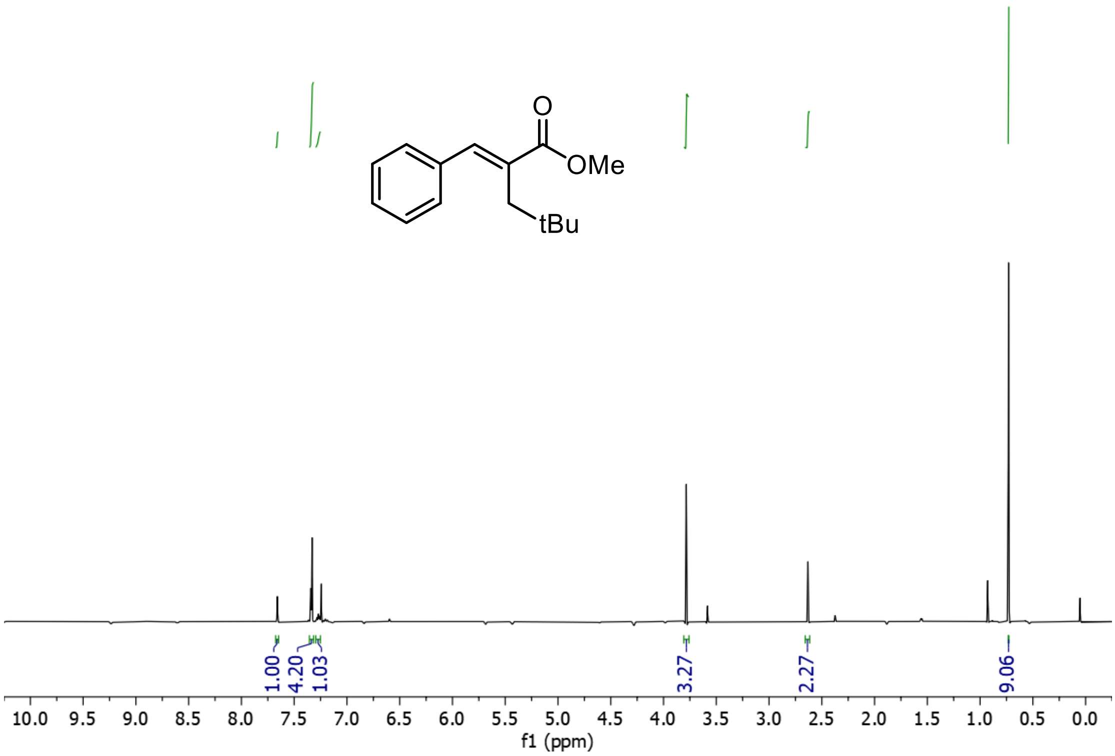

3ae ^{13}C NMR (100 MHz, CDCl$_3$)

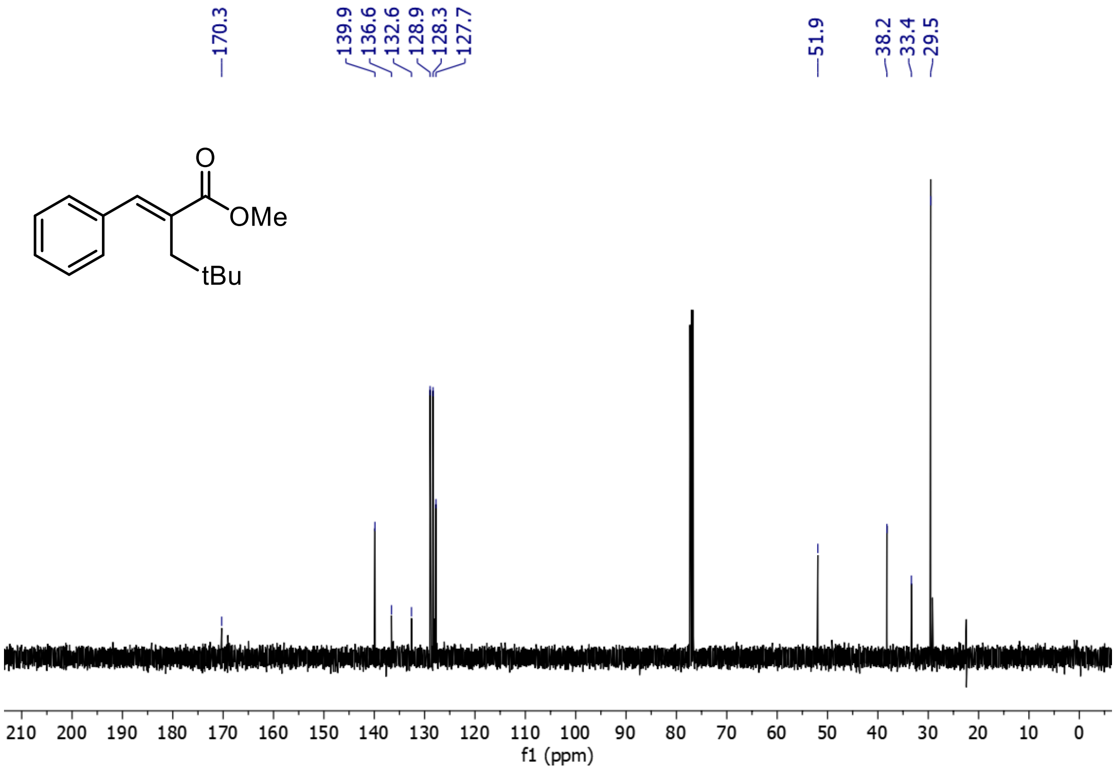

3af ^{1}H NMR (400 MHz, $CDCl_3$)

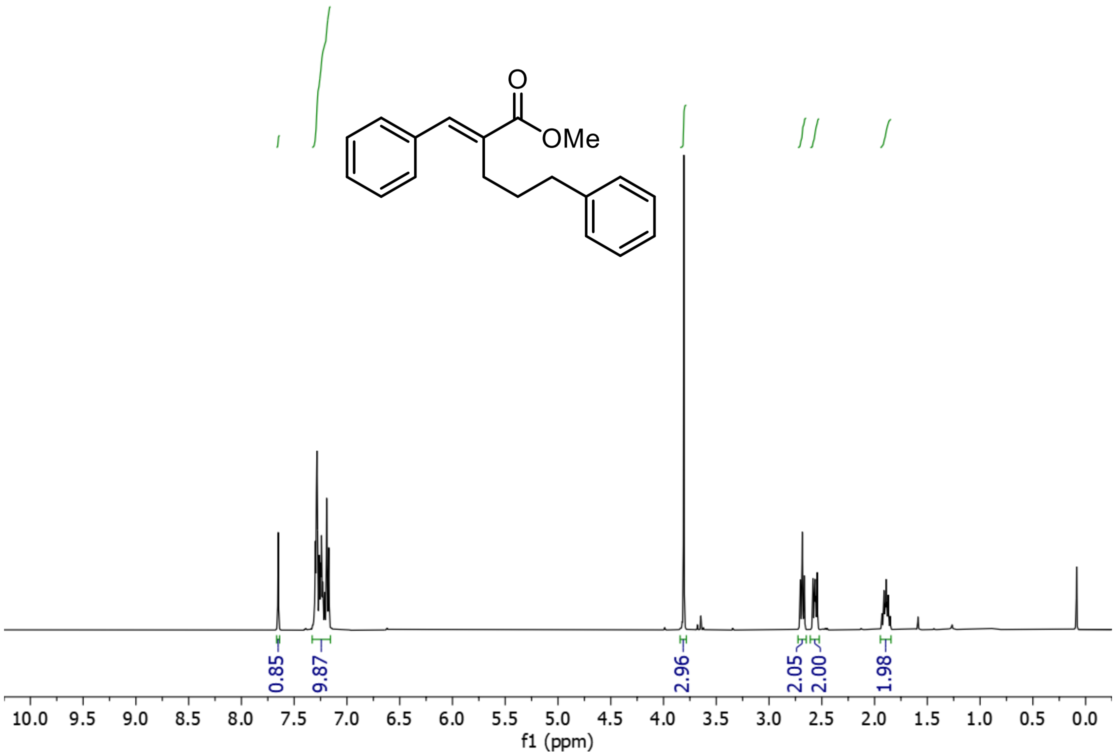

3af ^{13}C NMR (100 MHz, $CDCl_3$)

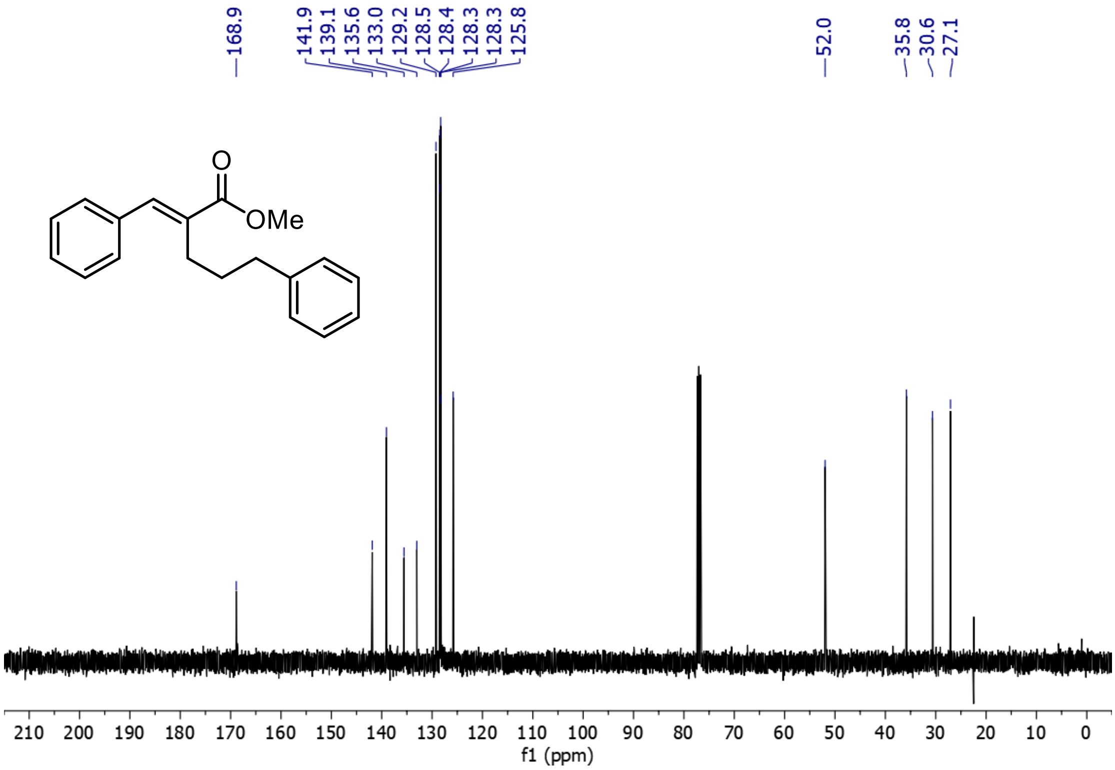

3ag ¹H NMR (400 MHz, CDCl₃)

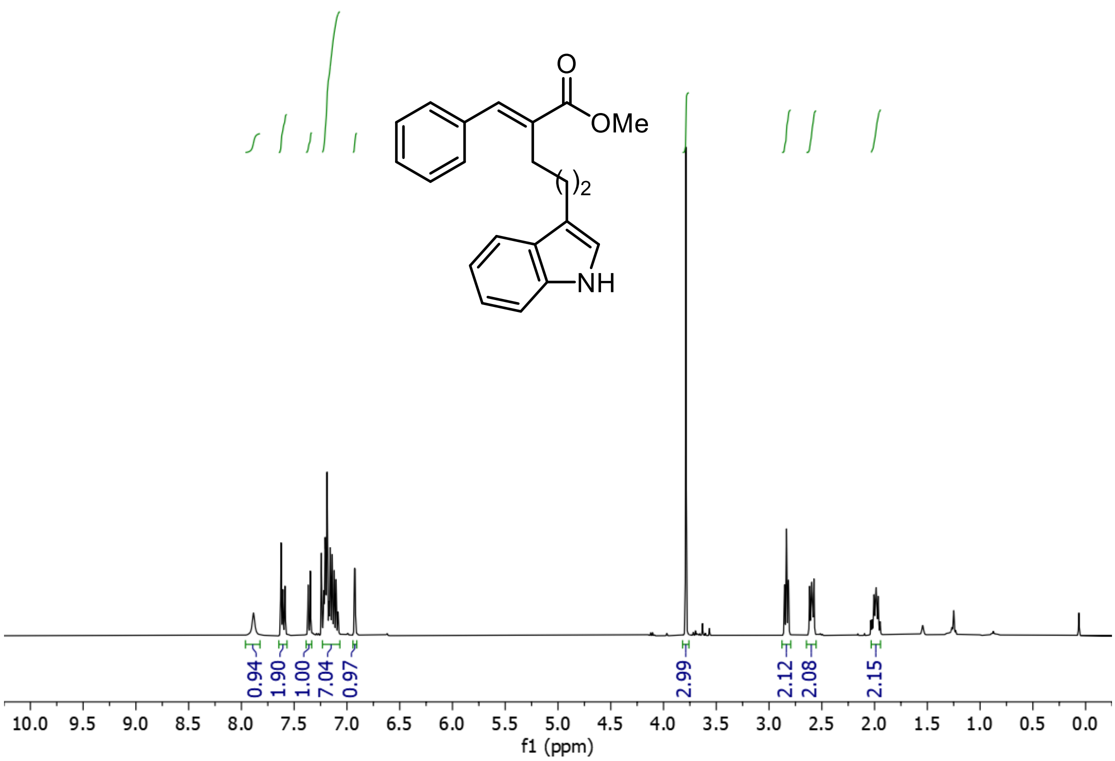

3ag ¹³C NMR (100 MHz, CDCl₃)

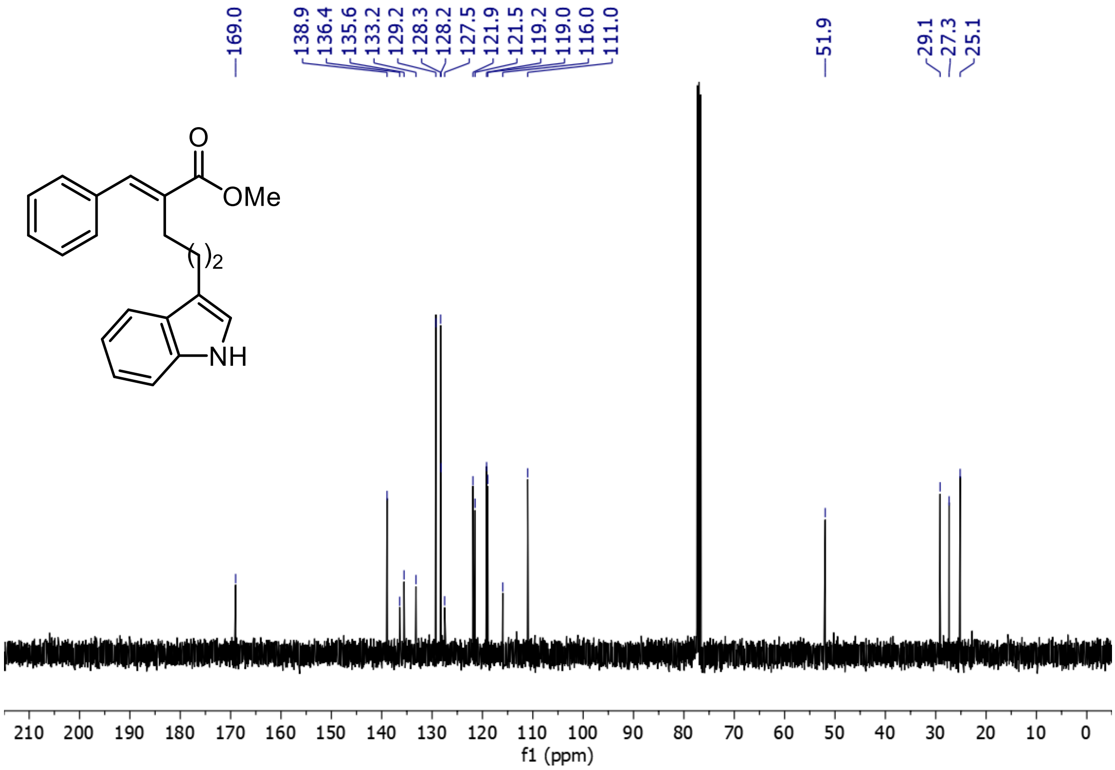

3ah ^{1}H NMR (400 MHz, $CDCl_3$)

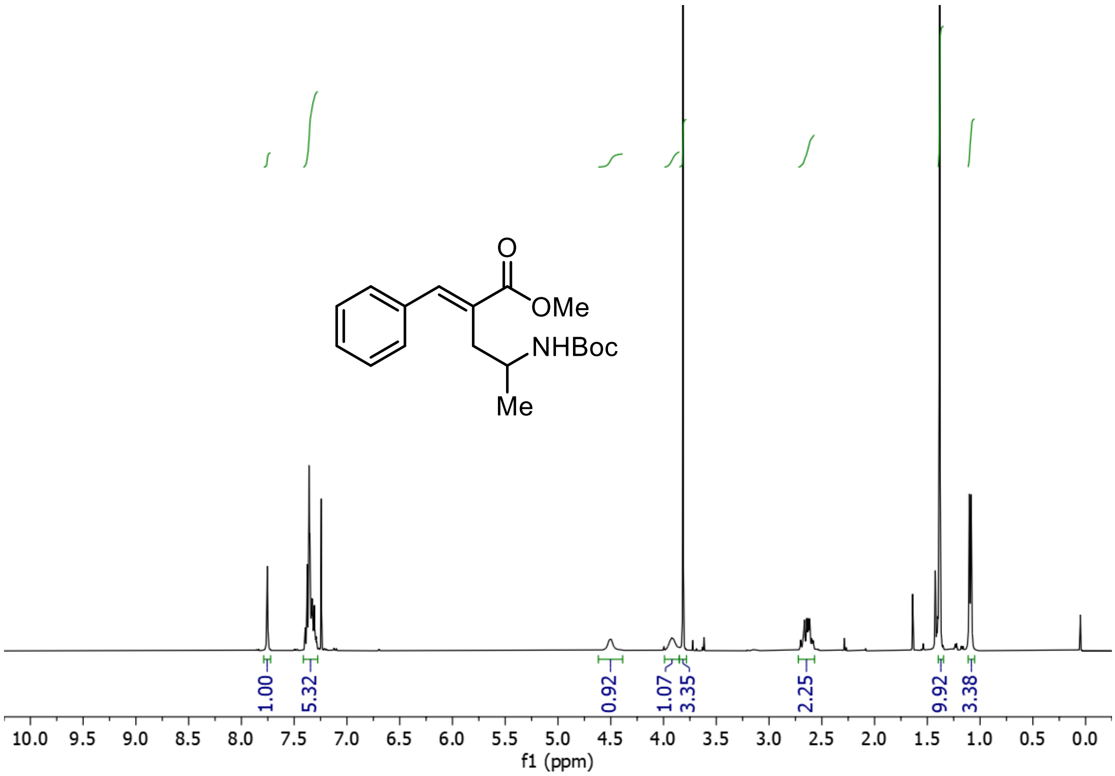

3ah ^{13}C NMR (100 MHz, $CDCl_3$)

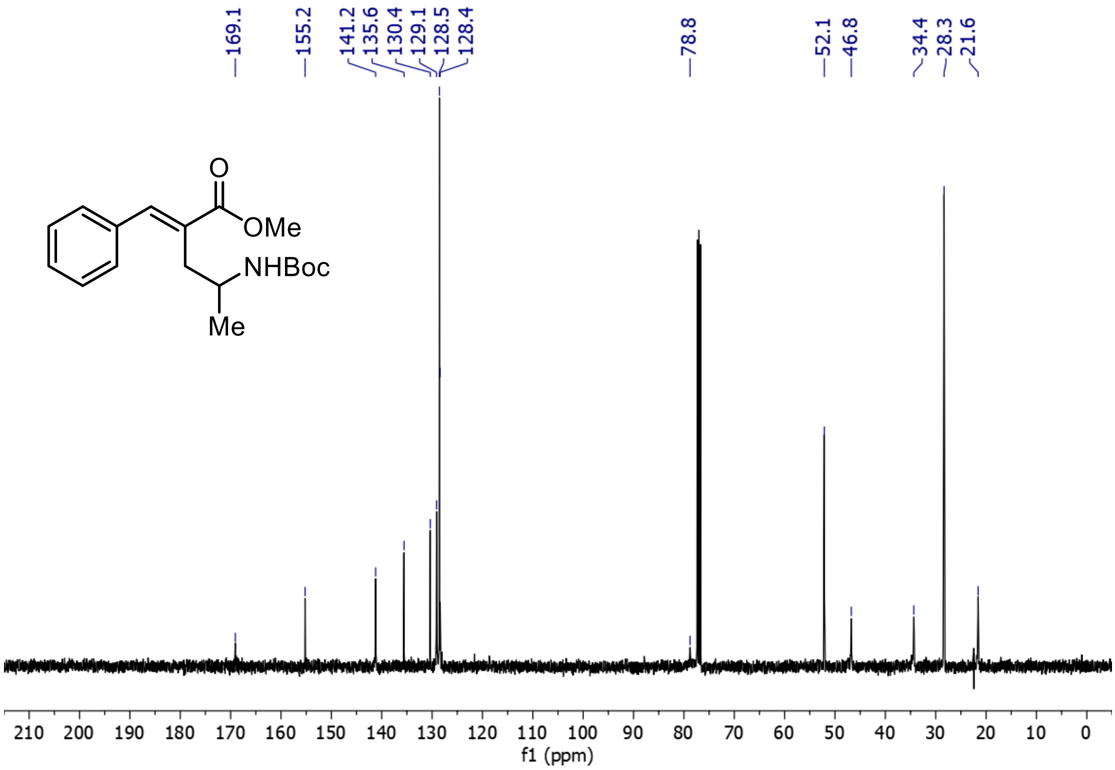

3ai ¹H NMR (400 MHz, CDCl₃)

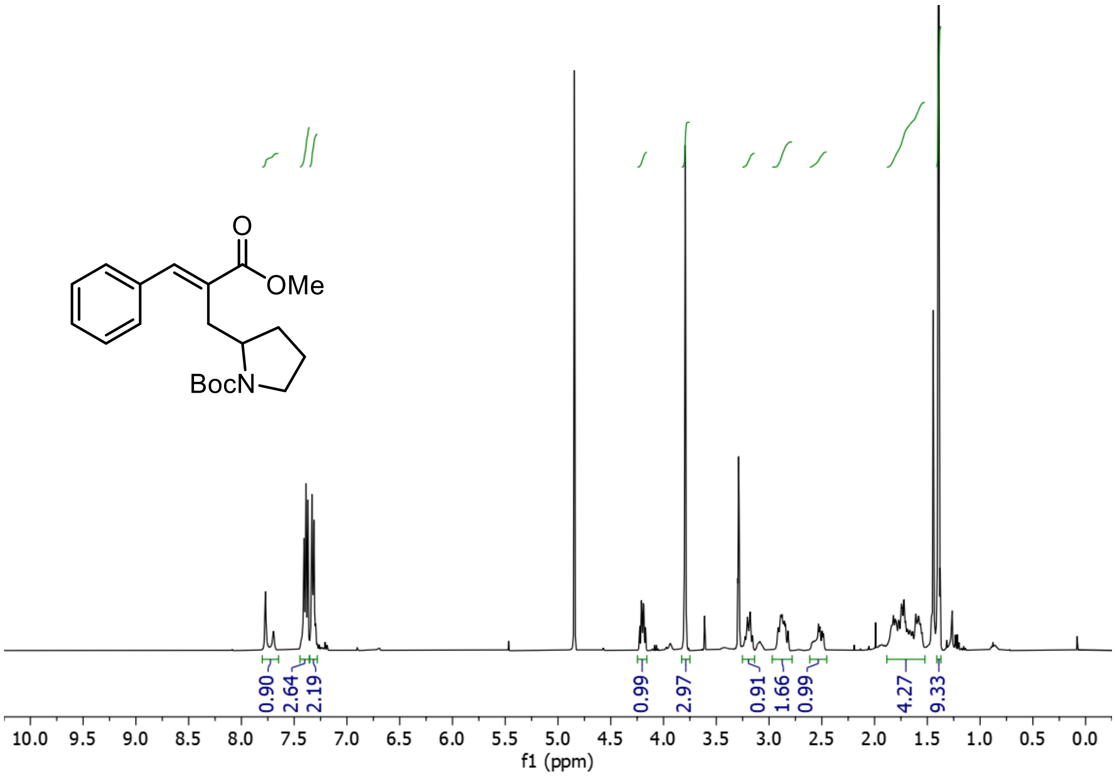

3ai ¹³C NMR (100 MHz, CDCl₃)

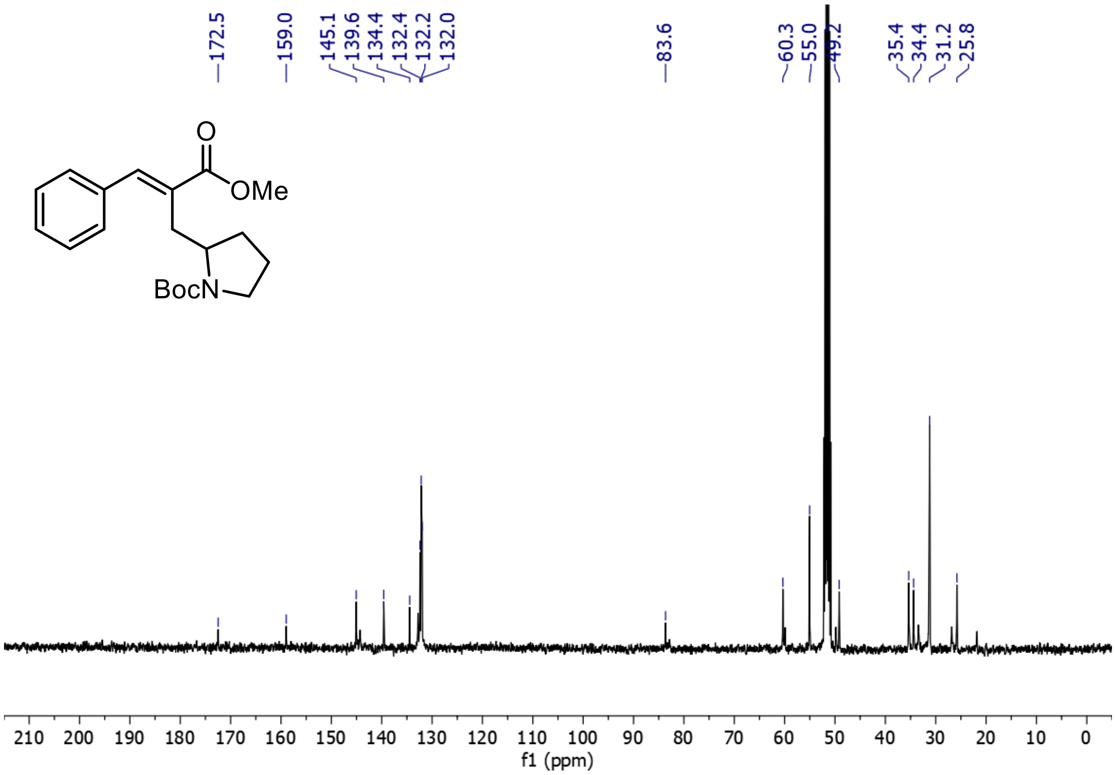

3aj ¹H NMR (400 MHz, CDCl₃)

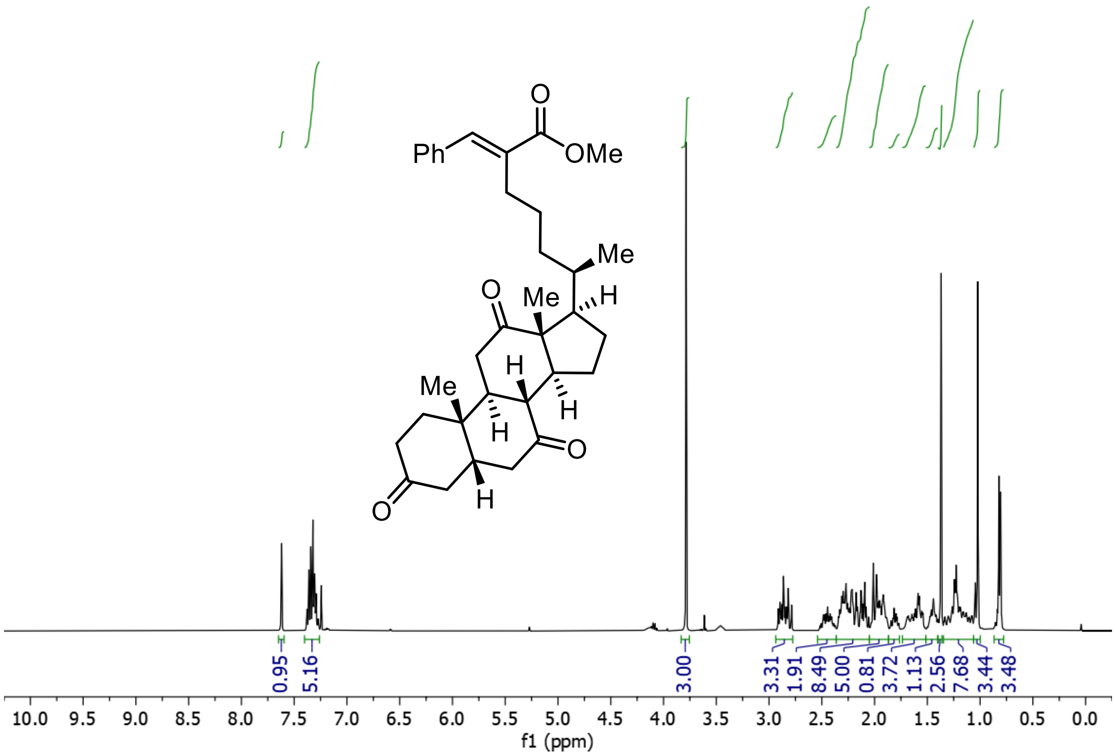

3aj ¹³C NMR (100 MHz, CDCl₃)

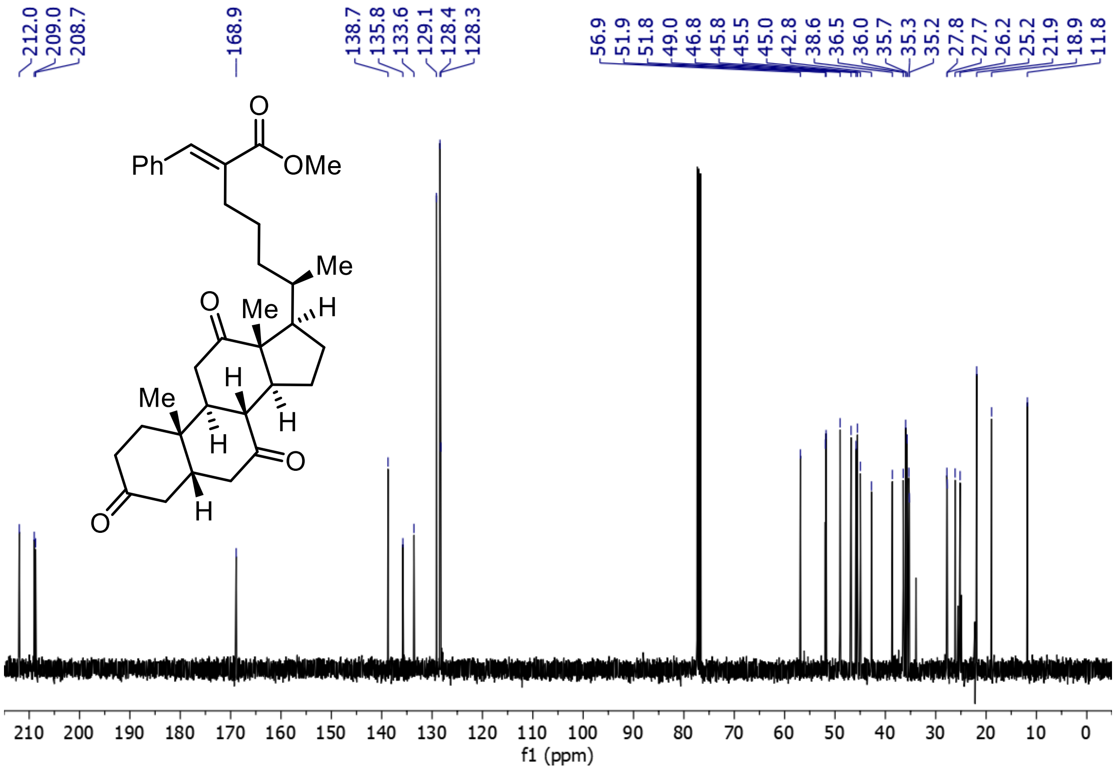

6aa ^{1}H NMR (400 MHz, $CDCl_3$)

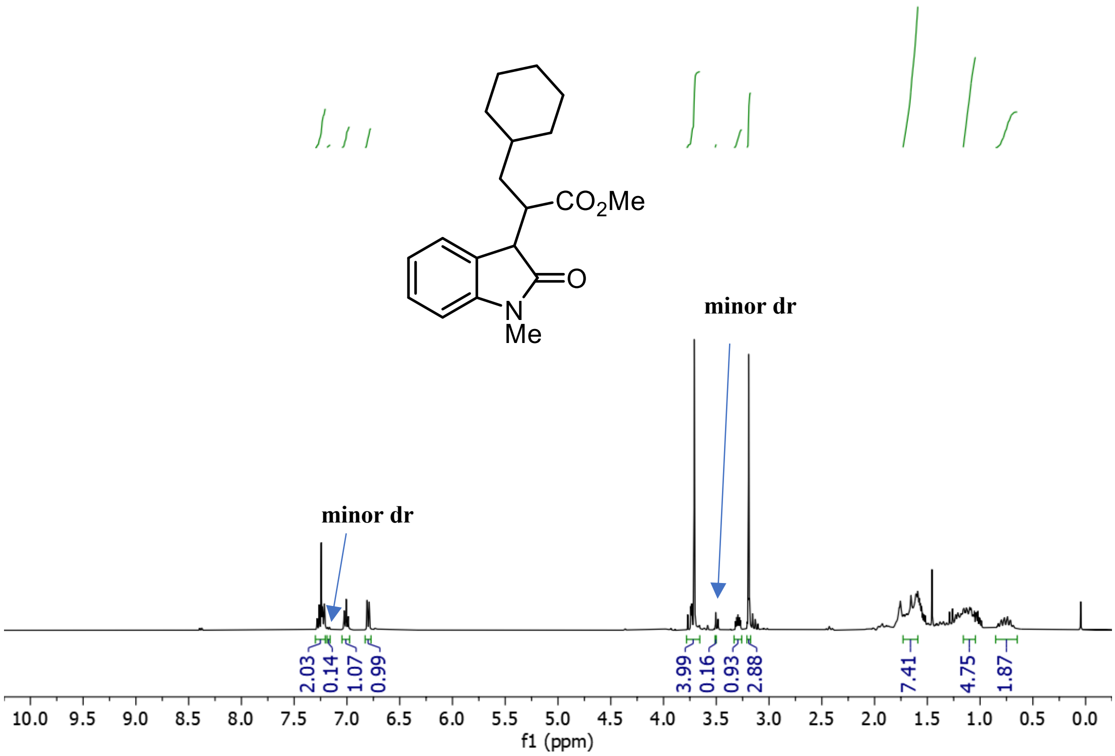

6aa ^{13}C NMR (100 MHz, $CDCl_3$)

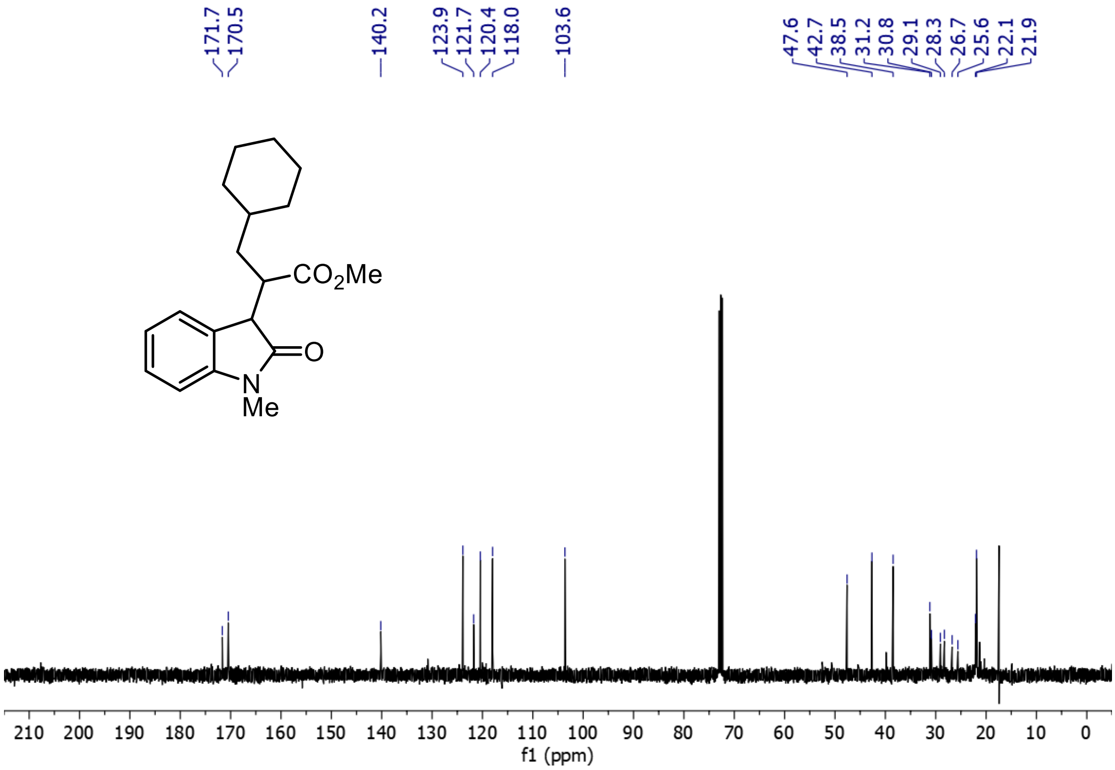

6ac ^{1}H NMR (400 MHz, $CDCl_3$)

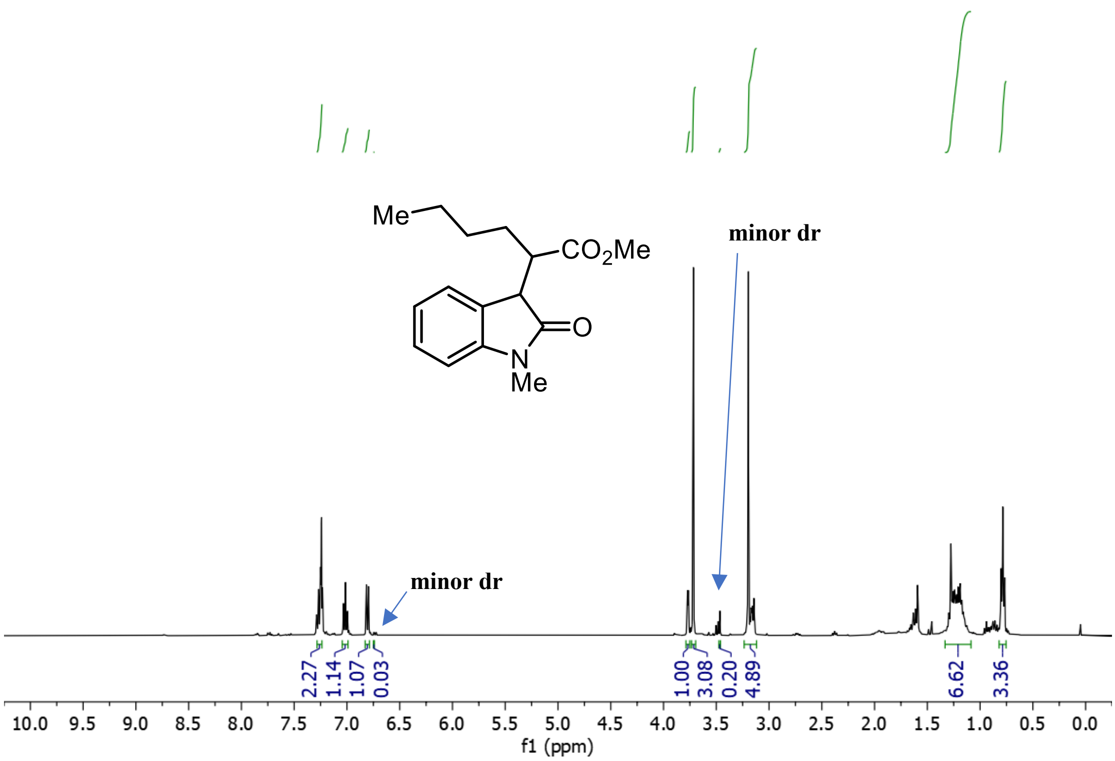

6ac ^{13}C NMR (100 MHz, $CDCl_3$)

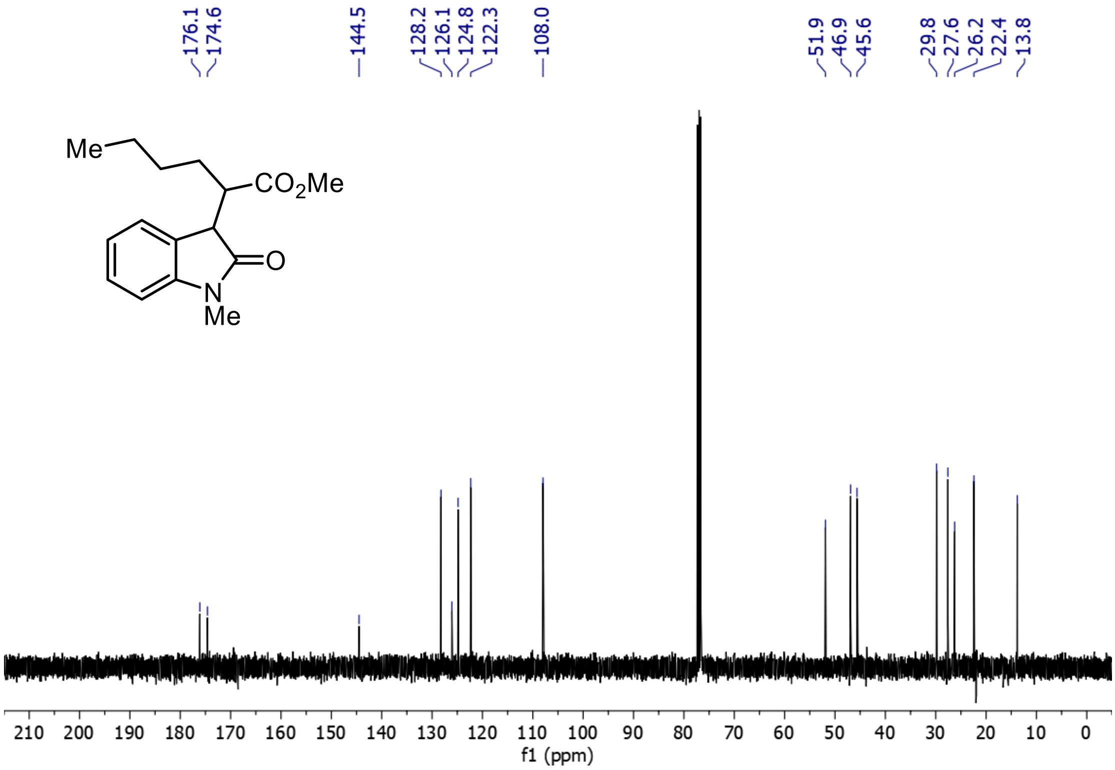

6ba ^{1}H NMR (400 MHz, $CDCl_3$)

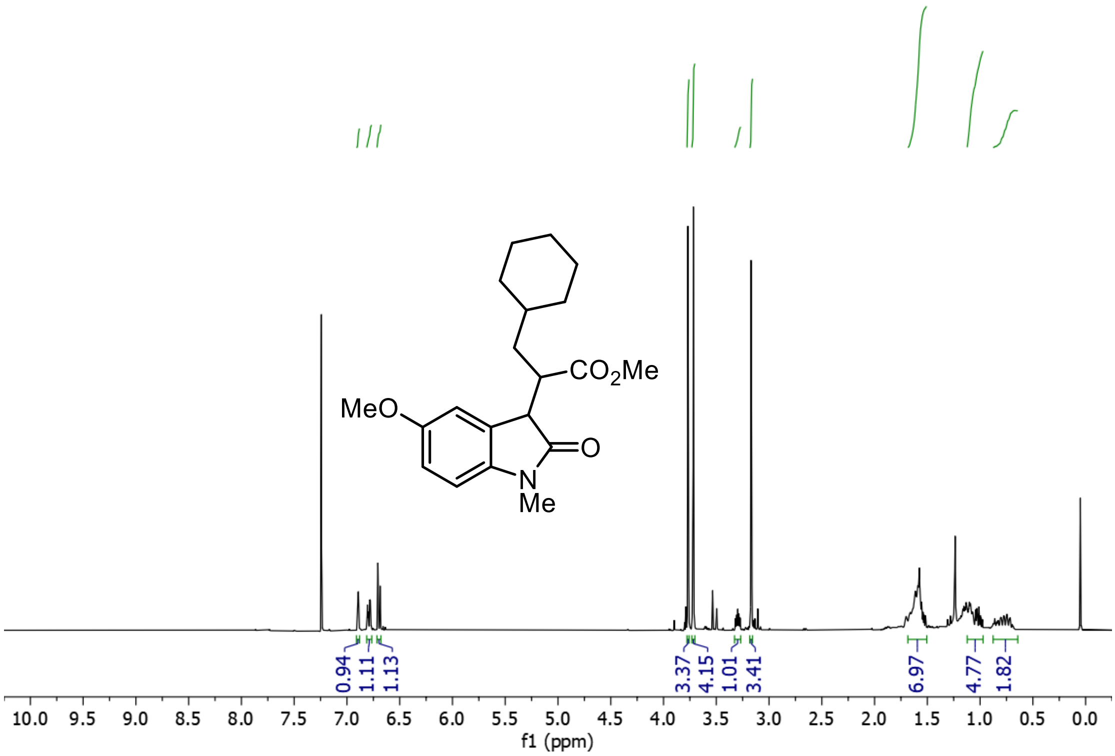

6ba ^{13}C NMR (100 MHz, $CDCl_3$)

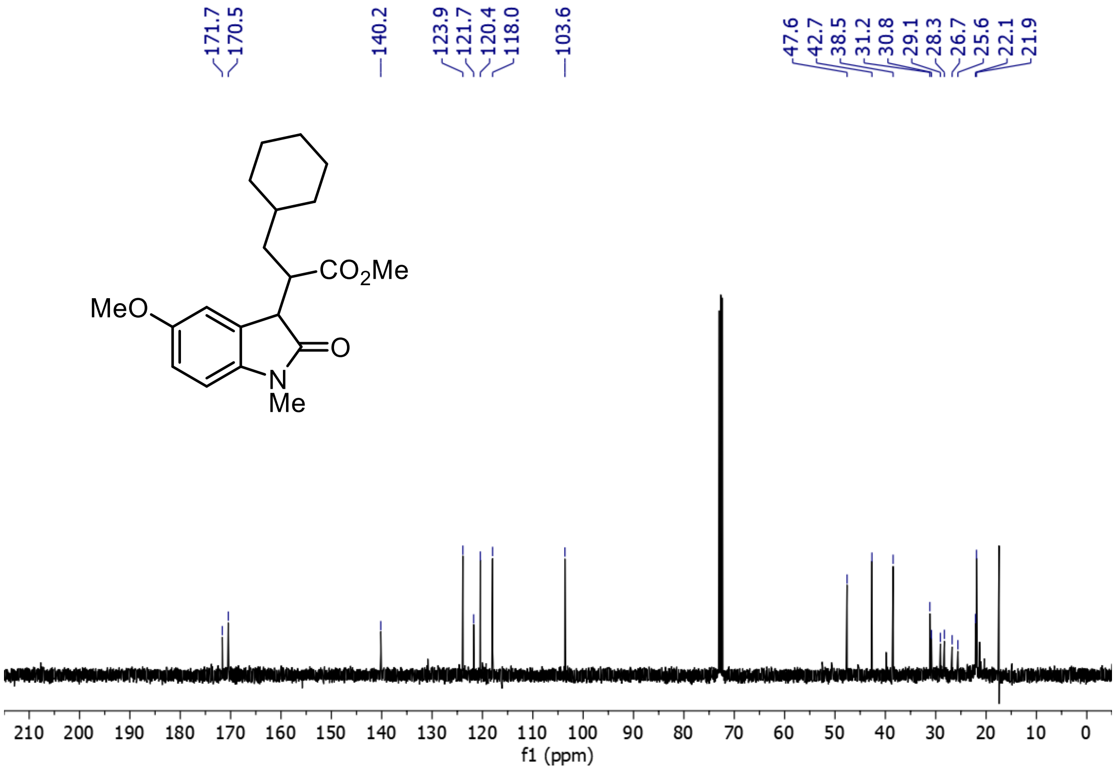

6bc ^{1}H NMR (400 MHz, $CDCl_3$)

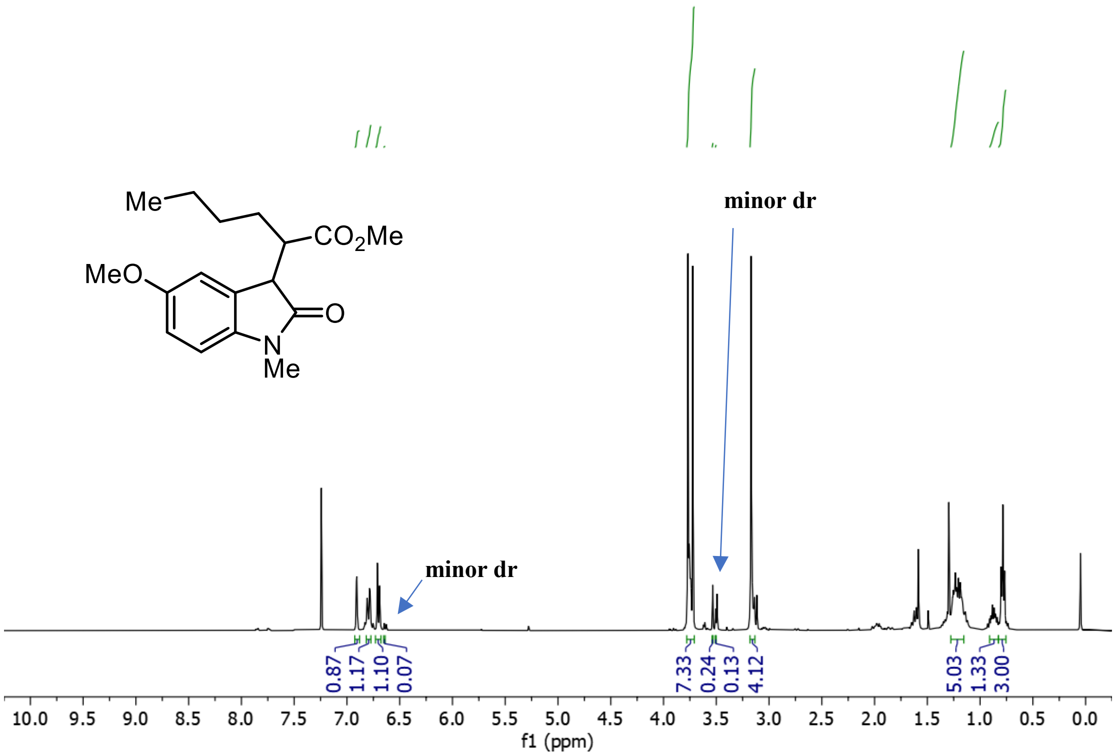

6bc ^{13}C NMR (100 MHz, $CDCl_3$)

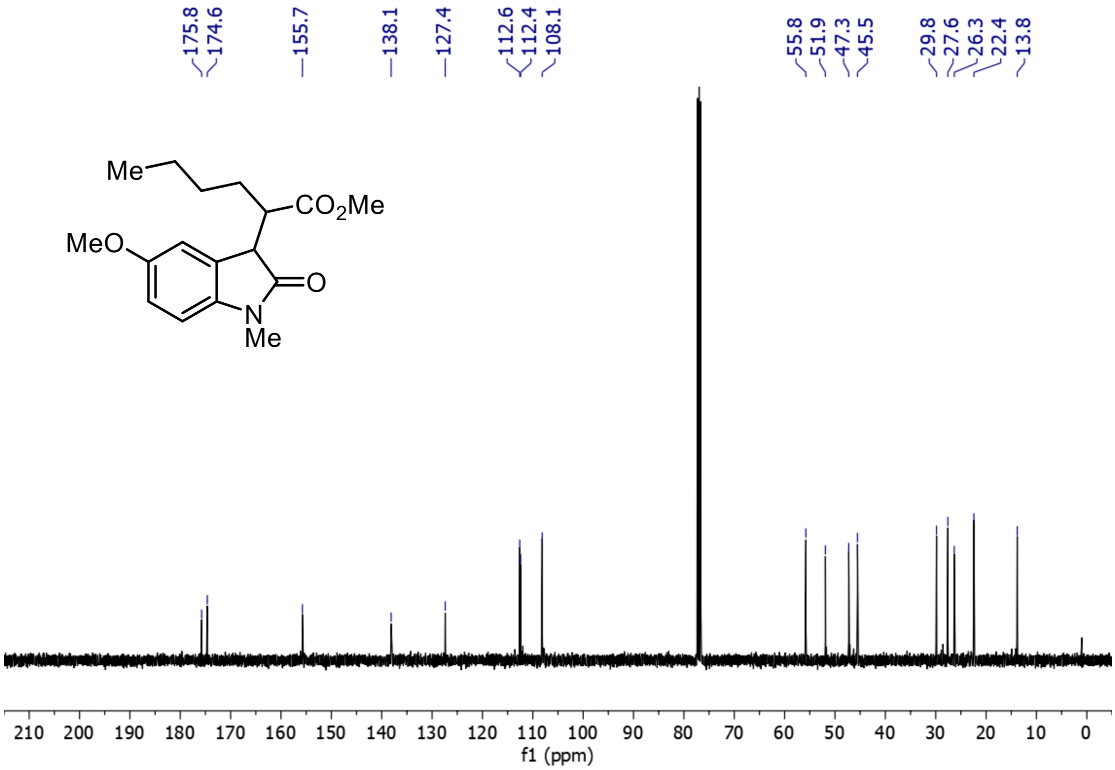

Copies of selected HRMS spectra

HRMS (APCI) of 3aa

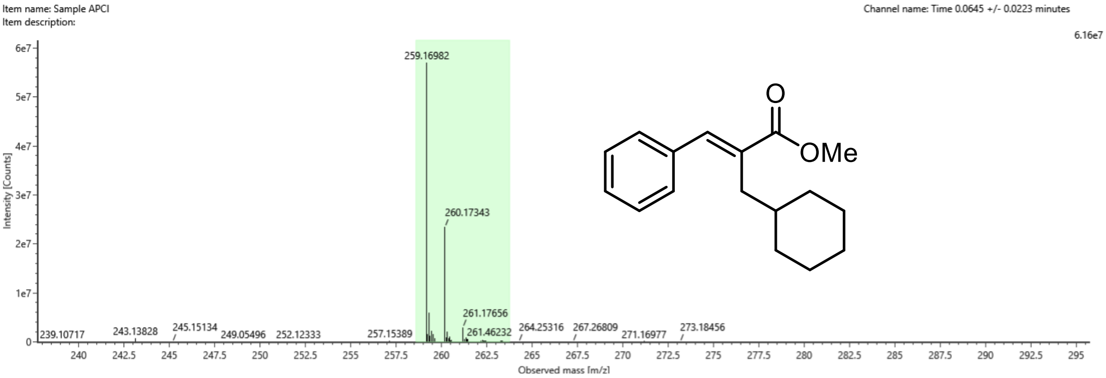

HRMS (APCI) of 3ca

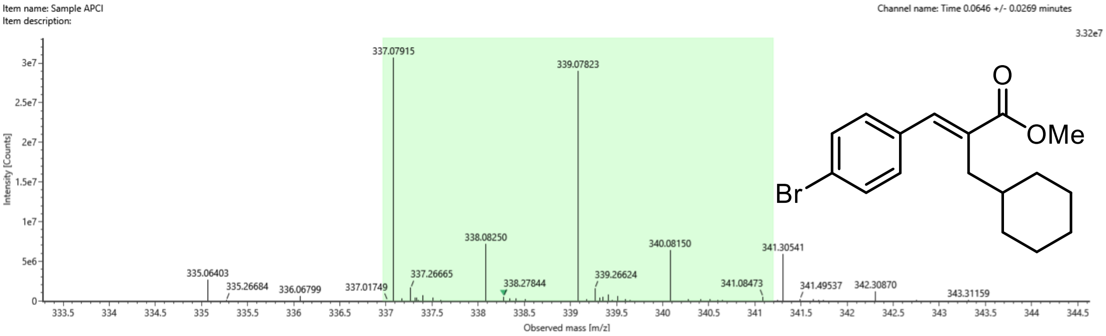

HRMS (APCI) of 3da

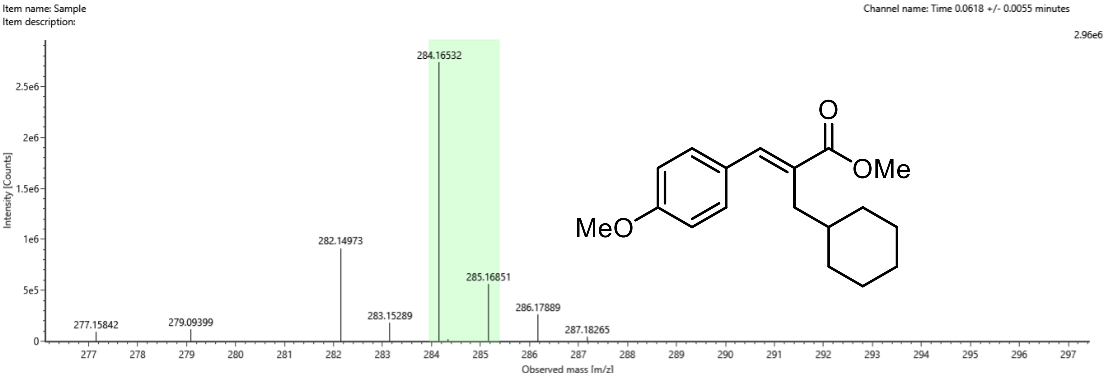

HRMS (APCI) of 3ea

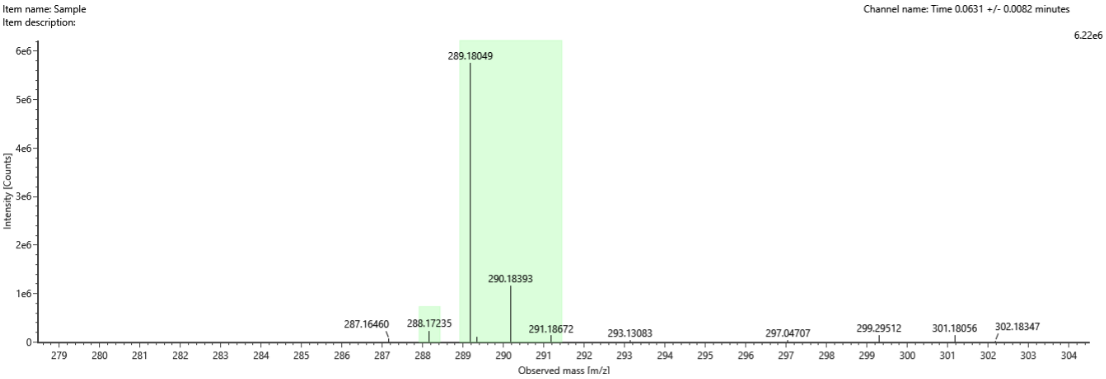

HRMS (APCI) of 3fa

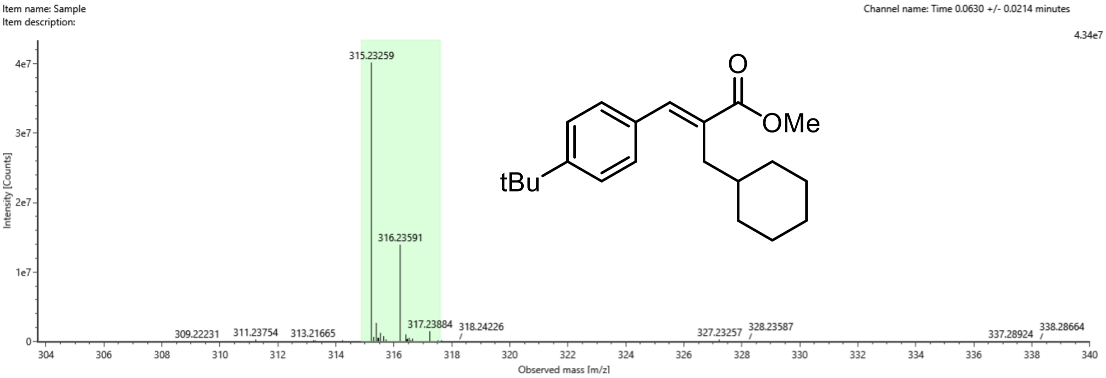

HRMS (APCI) of 3ga

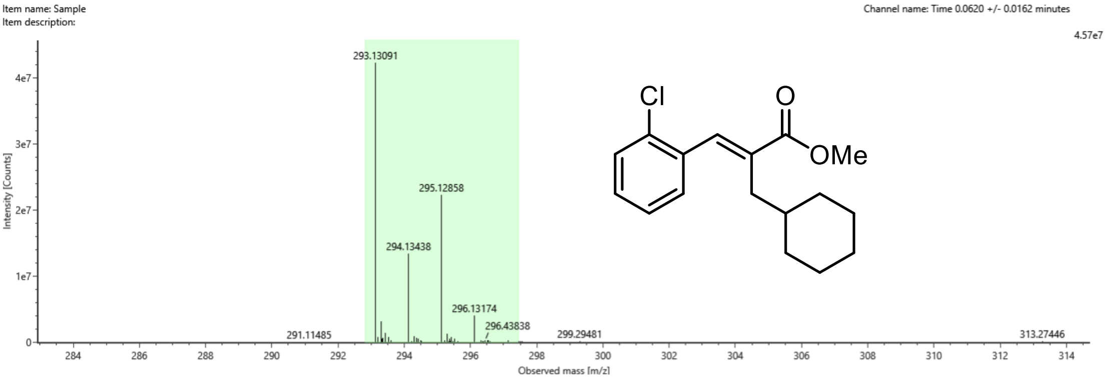

HRMS (APCI) of 3ha

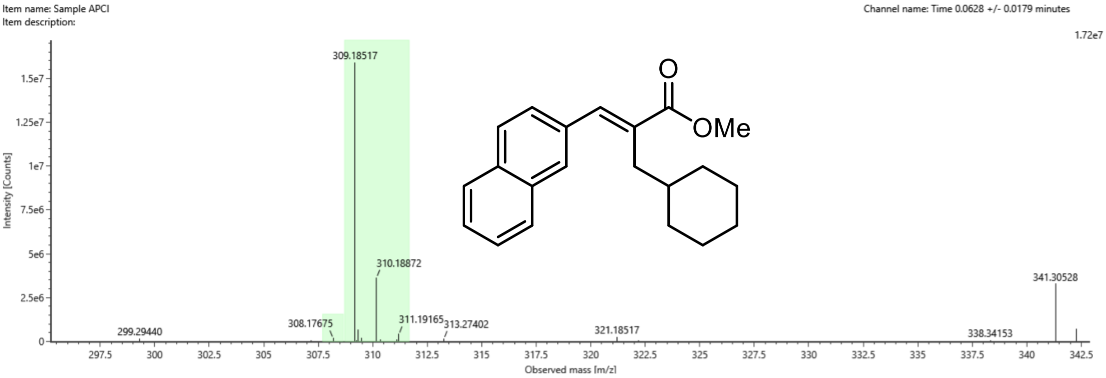

HRMS (APCI) of 3ia

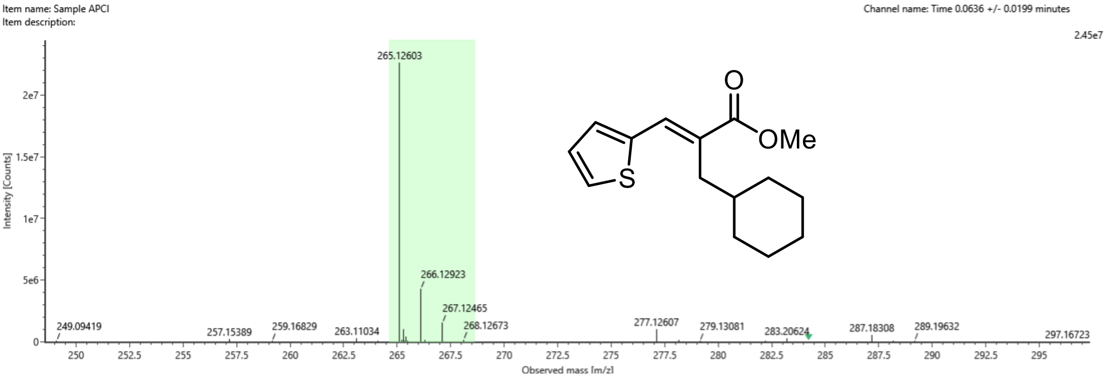

HRMS (APCI) of 3ja'

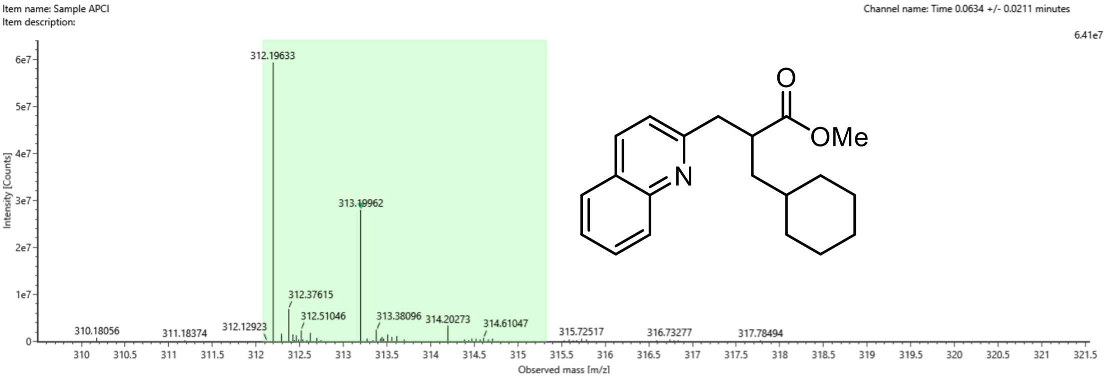

HRMS (APCI) of 3ka

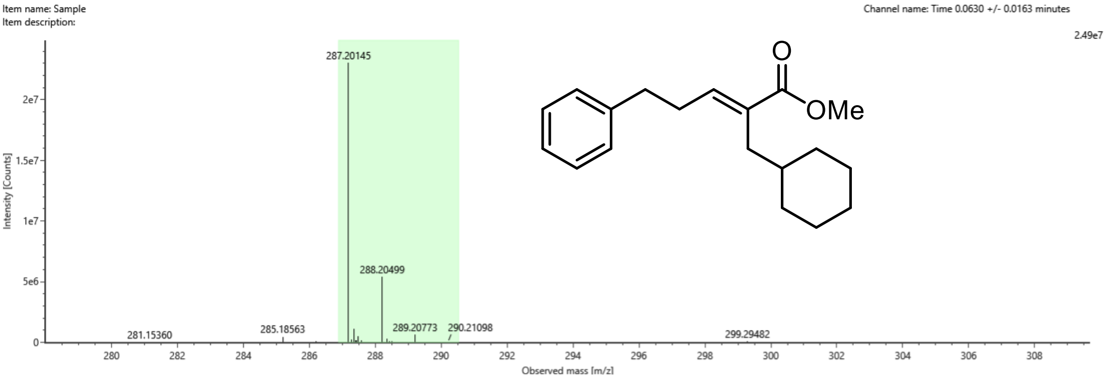

HRMS (APCI) of 3ma

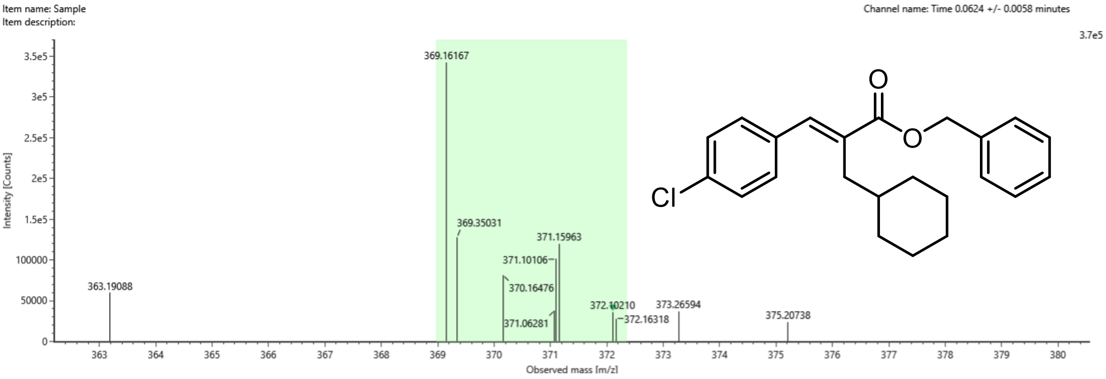

HRMS (APCI) of 3na

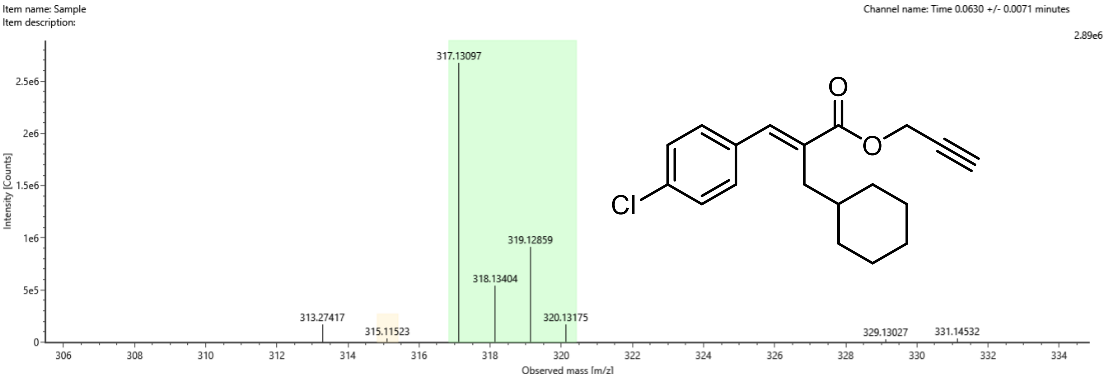

HRMS (APCI) of 3oa

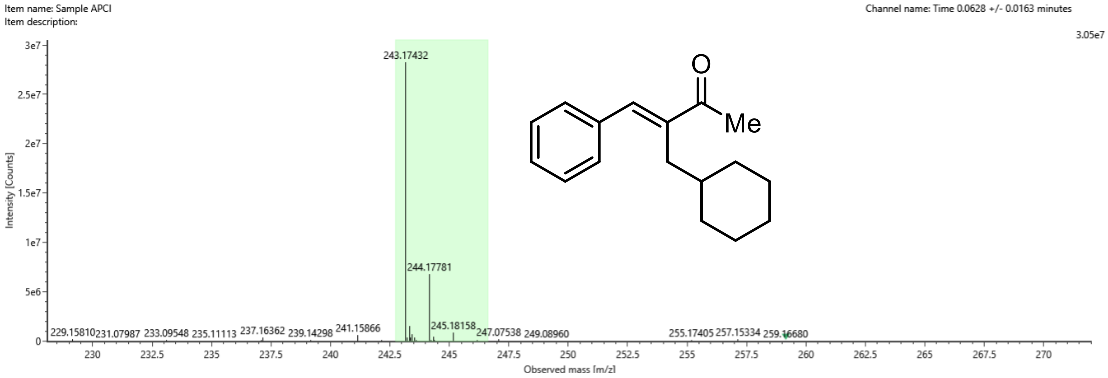

HRMS (APCI) of 3ab

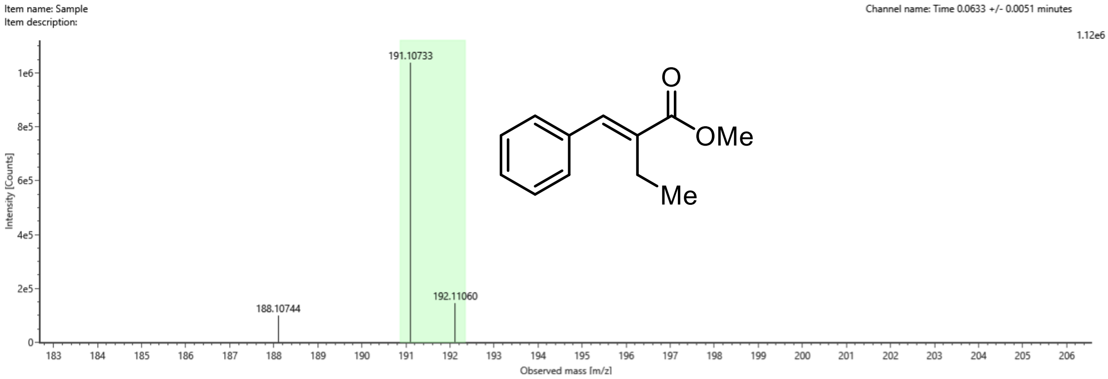

HRMS (APCI) of 3ac

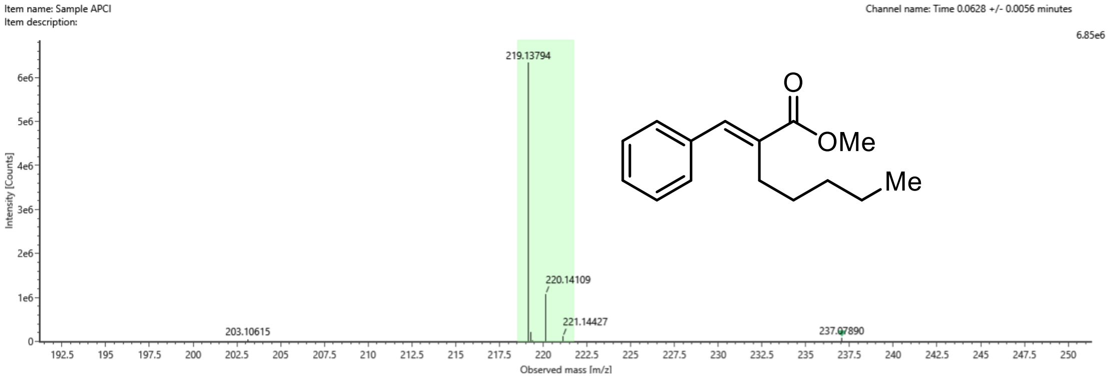

HRMS (APCI) of 3ad

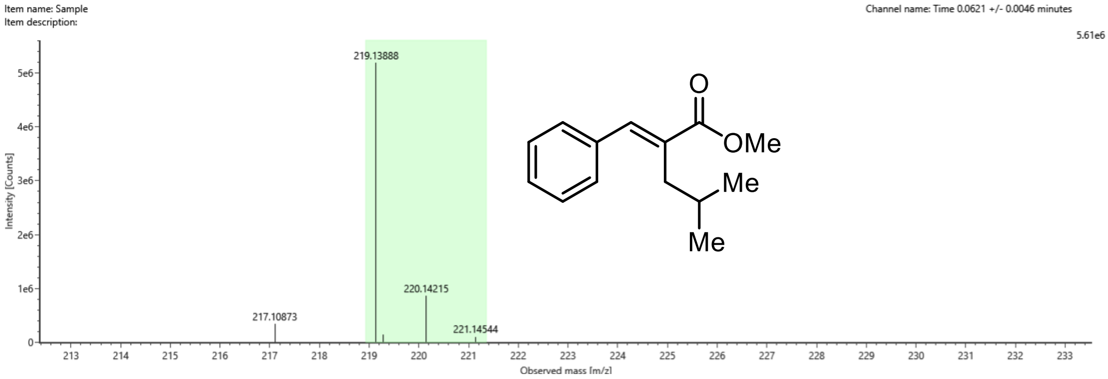

HRMS (APCI) of 3ae

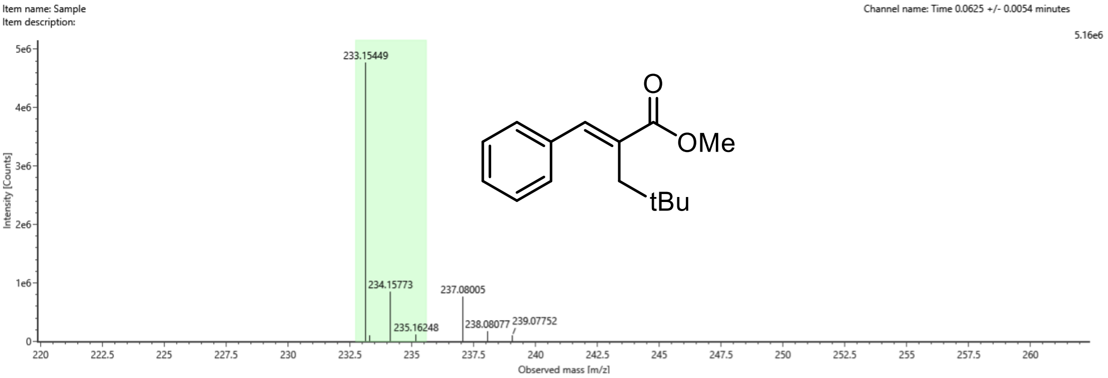

HRMS (APCI) of 3af

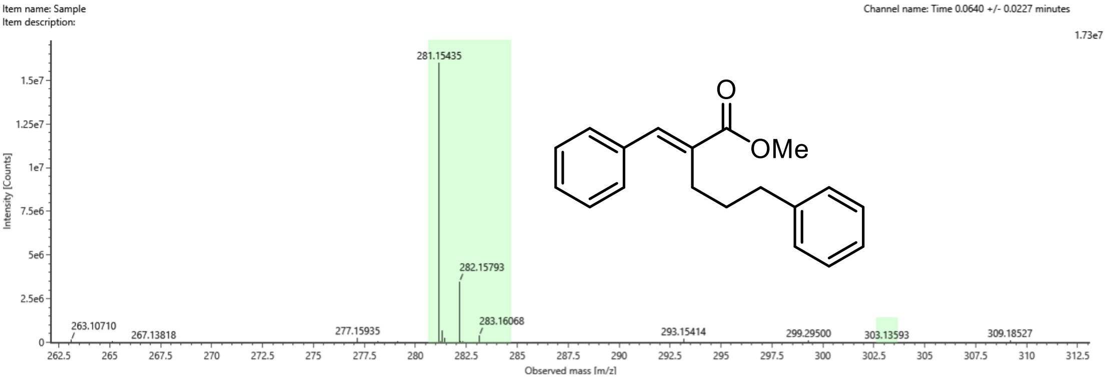

HRMS (APCI) of 3ag

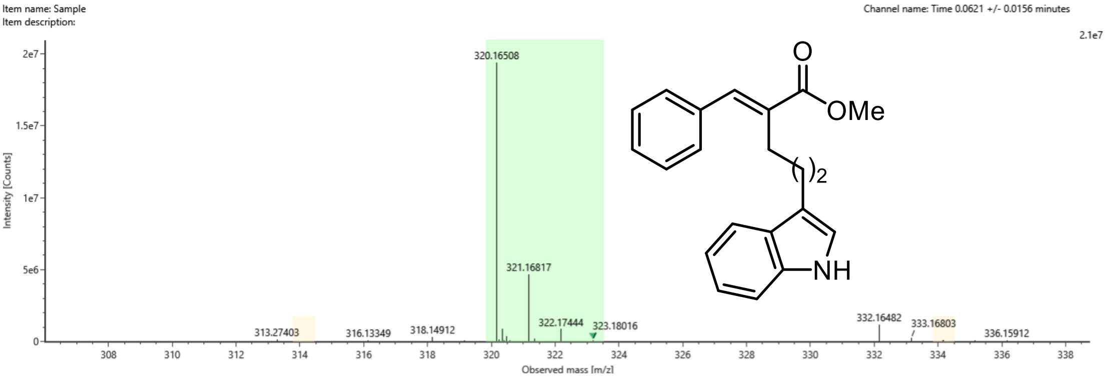

HRMS (APCI) of 6aa

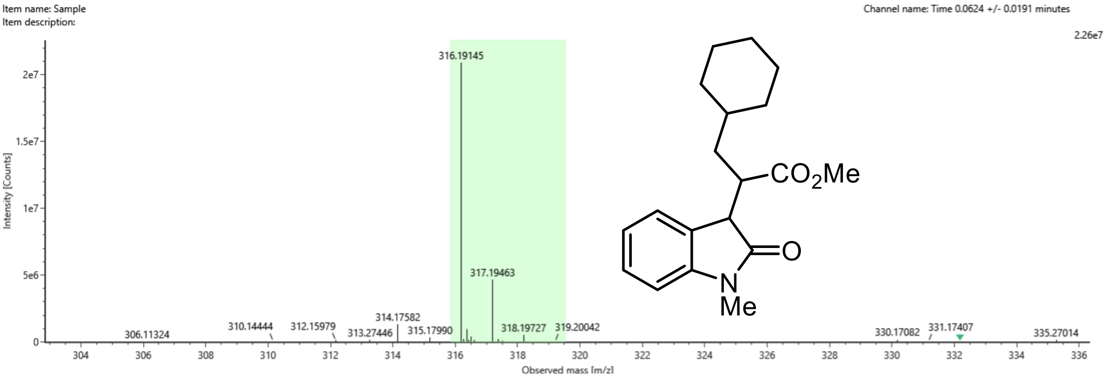

HRMS (APCI) of 6ac

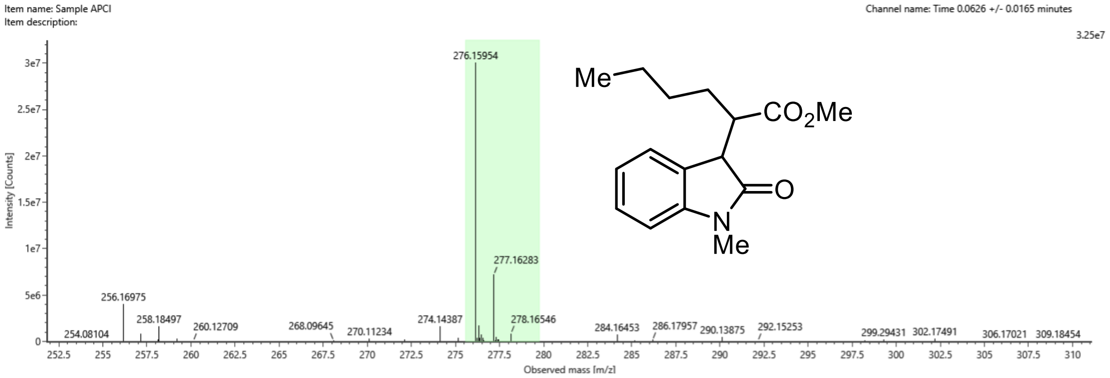

HRMS (APCI) of 6ba

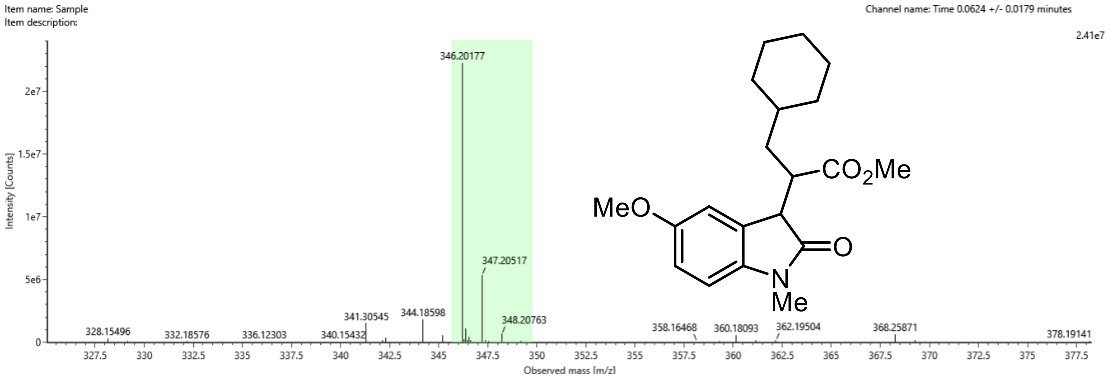

HRMS (APCI) of 6bc

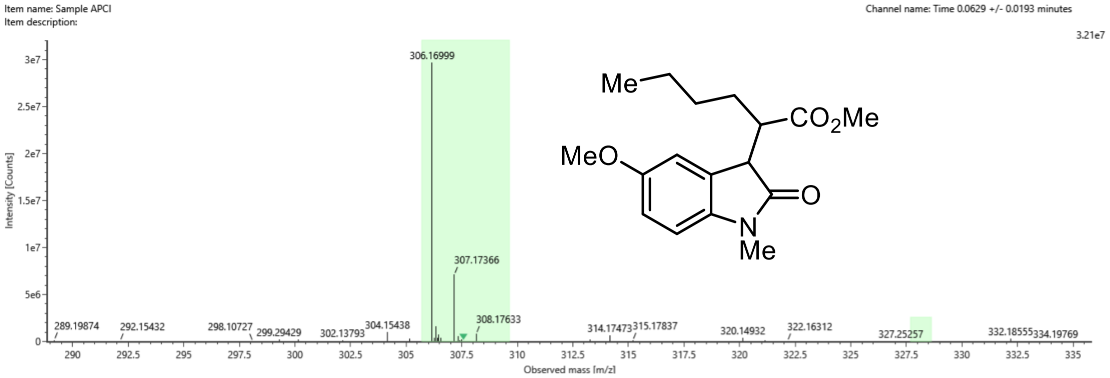

References

[1] Batchu, H.; Bhattacharyya, S.; Batra, S. Iodine-Mediated Intramolecular Electrophilic Aromatic Cyclization in Allylamines: A General Route to Synthesis of Quinolines, Pyrazolo[4,3-*b*]pyridines, and Thieno[3,2-*b*]pyridines, *Org. Lett.* **2012**, *14*, 6330-6333 and references therein (see Supporting Information).

[2] Chen, X.; Luo, X.; Peng X.; Guo, J.; Zai, J.; Wang, P. Catalyst-Free Decarboxylation of Carboxylic Acids and Deoxygenation of Alcohols by Electro-Induced Radical Formation**,** *Chem. Eur. J.* **2020**, *26*, 3226-3230 and references therein (see Supporting Information).

[3] Cheng, W.-M.; Shang, R.; Fu, M.-C.; Fu, Y. Photoredox-Catalysed Decarboxylative Alkylation of N-Heteroarenes with *N*-(Acyloxy)phthalimides, *Chem. Eur. J.* **2017**, *23*, 2537-2541.

[4] Li, J.; Tan, S. S.; Kyne, S. H.; Chan, P. W. H. Minisci-Type Alkylation of *N* -Heteroarenes by *N* -(Acyloxy)phthalimide Esters Mediated by a Hantzsch Ester And Blue LED Light, *Adv. Synth. Catal.* **2021**, *364*, 802-810.

[5] Shen, M.-L.; Shen, Y.; Wang, P.-S. Merging Visible-Light Photoredox and Chiral Phosphate Catalysis for Asymmetric Friedel–Crafts Reaction with in Situ Generation of *N*-Acyl Imines, *Org. Lett.* **2019**, *21*, 2993-2997.

[6] Ye, H.; Zhao, H.; Ren, S.; Ye, H.; Cheng, D.; Li, X.; Xu, X. The coupling of alkylboronic acids and esters with Baylis–Hillman derivatives by Lewis base/photoredox dual catalysis, *Tetrahedron Lett.* **2019**, *60*, 1302-1305.
